# Macrophages Switch to an Osteo‐Modulatory Profile Upon RANKL Induction in a Medaka (*Oryzias latipes*) Osteoporosis Model

**DOI:** 10.1002/jbm4.10409

**Published:** 2020-10-01

**Authors:** Quang Tien Phan, Ranran Liu, Wen Hui Tan, Nurgul Imangali, Benedict Cheong, Manfred Schartl, Christoph Winkler

**Affiliations:** ^1^ Department of Biological Sciences and Centre for Bioimaging Sciences National University of Singapore Singapore Singapore; ^2^ Department of Developmental Biochemistry, Biocenter University of Würzburg Würzburg Germany; ^3^ The Xiphophorus Genetic Stock Center Texas State University San Marcos Texas USA

**Keywords:** MACROPHAGES, OSTEOCLASTS, RANKL, TNFα, BONE RESORPTION, BONE HOMEOSTASIS

## Abstract

In mammals, osteoclasts differentiate from macrophages in the monocyte lineage. Although many factors driving osteoclast formation are known, the detailed processes underlying precursor recruitment, differentiation, and interaction of macrophages with other cell types involved in bone remodeling are poorly understood. Using live imaging in a transgenic medaka osteoporosis model, where ectopic osteoclasts are induced by RANKL expression, we show that a subset of macrophages is recruited to bone matrix to physically interact with bone‐forming osteoblast progenitors. These macrophages subsequently differentiate into *cathepsin K‐* (*ctsk‐*) positive osteoclasts. One day later, other macrophages are recruited to clear dying osteoclasts from resorbed bone by phagocytosis. To better understand the molecular changes underlying these dynamic processes, we performed transcriptome profiling of activated macrophages upon RANKL induction. This revealed an upregulation of several bone‐related transcripts. Besides osteoclast markers, we unexpectedly also found expression of osteoblast‐promoting signals in activated macrophages, suggesting a possible non‐cell autonomous role in osteogenesis. Finally, we show that macrophage differentiation into osteoclasts is dependent on inflammatory signals. Medaka deficient for TNFα or treated with the TNFα‐inhibitor pentoxifylline exhibited impaired macrophage recruitment and osteoclast differentiation. These results show the involvement of inflammatory signals and the dynamics of a distinct subset of macrophages during osteoclast formation. © 2020 The Authors. *JBMR Plus* published by Wiley Periodicals LLC on behalf of American Society for Bone and Mineral Research.

## Graphical abstract

Using live imaging in a medaka fish osteoporosis model, we report the dynamic recruitment of macrophages to bone matrix, their close interaction with osteoblasts and subsequent differentiation into osteoclasts. These processes require TNFa and involve significant transcriptomic changes as activated macrophages switch from an immune to a bone‐modulatory signature. Based on this signature, we propose a non‐cell autonomous role of RANKL‐activated macrophages in promoting cartilage and bone formation to achieve bone homeostasis.

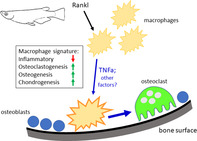

## Introduction

Since their initial description in 1883,^(^ ^1^ ^)^ macrophages have been intensively studied for their involvement in various physiological processes including embryogenesis, infection, tissue regeneration, and bone homeostasis (reviewed in Wynn and colleagues^(^ ^2^ ^)^). Bone marrow‐derived monocytes and macrophages have been identified as precursors of *cathepsin K‐* (*ctsk‐*) positive osteoclasts, which digest mineralized matrix and thereby function in bone resorption.^(^ ^3^, ^4^ ^)^ On the other hand, osteal macrophages, a subpopulation of specialized bone‐homing macrophages, were shown to have essential roles in bone formation during regeneration and fracture healing.^(^ ^5^, ^6^ ^)^ This cell type also modulates cytokine expression and performs clearance of apoptotic cells in a process known as efferocytosis.^(^ ^7^ ^)^ During an inflammation episode, the activation of macrophages often includes dynamic regulation of proinflammatory cytokines, especially IL1β and TNFα. These cytokines trigger an immune response and recruit other immune cells to inflammation sites.^(^ ^7^, ^8^ ^)^ TNFα, a member of the tumor necrosis factor superfamily, is produced by different cell types such as monocytes, neutrophils, and adipocytes.^(^ ^9^, ^10^ ^)^ It plays important roles in cell proliferation, wound healing, and cancer progression.^(^ ^8^, ^11^ ^)^ TNFα is also found predominantly expressed in activated macrophages in inflammatory diseases such as rheumatoid arthritis.^(^ ^12^ ^)^ Inhibition of this cytokine has been an effective therapy to alleviate bone inflammatory symptoms.^(^ ^13^ ^)^ Patients with osteoporosis, who also have elevated serum levels of IL1β and TNFα, often experience severe bone loss and fractures that result from hyperactivity of osteoclasts.^(^ ^14^ ^)^ Several studies have shown that in the presence of macrophage colony stimulating factor (M‐CSF), TNFα and RANKL synergistically or independently stimulate osteoclast formation via their respective receptors.^(^ ^15^, ^16^, ^17^ ^)^ However, how these cytokines control the dynamics of osteoclast precursor recruitment and differentiation in vivo remains unclear, mostly because commonly used animal models have limited accessibility for live imaging.

In this study, we used a transgenic medaka osteoporosis model, where excessive osteoclast formation is triggered by inducible RANKL expression.^(^ ^18^ ^)^ Upon RANKL induction, macrophages migrate to the vertebral column where they eventually mature into *ctsk*‐positive osteoclasts.^(^ ^19^ ^)^ In the present study, we describe the dynamics of macrophage migration and their close interaction with bone‐lining osteoblasts. We report that *tnfa* is upregulated in activated macrophages during recruitment and that deletion of *tnfa* or treatment with the *tnfa* transcription inhibitor pentoxifylline (PTX) blocks macrophage recruitment and osteoclast differentiation. Transcriptome profiling further revealed that the cytokine receptor gene *il22ra2b* was downregulated when macrophages differentiate into osteoclasts. Together, these findings suggest that inflammatory signals are initially required for macrophage recruitment towards bone matrix under osteoporotic conditions. Subsequently, these signals are downregulated to allow differentiation of recruited cells into osteoclasts. Interestingly, we also find that upon RANKL induction, macrophages upregulate genes that are known to control chondrocyte and osteoblast differentiation in a non‐cell autonomous manner. This suggests an involvement of activated macrophages in bone remodeling and homeostasis under pathological conditions.

## Materials and Methods

All experiments were performed according to protocols approved by the Institutional Animal Care and Use Committee (IACUC) of the National University of Singapore (NUS; protocol numbers R14‐293, R18‐0562, and BR15‐0119).

### Transgenic and mutant medaka lines

Transgenic *rankl:HSE:cfp/ctsk:GFP* medaka embryos were grown and subjected to RANKL induction by heat‐shock as previously described.^(^ ^18^ ^)^ Generation of the *mpeg1:mCherry‐F* macrophage reporter line was described before.^(^ ^19^ ^)^ Briefly, a 2.1‐kb sequence of the *mpeg1* promoter including the endogenous ATG was amplified from genomic DNA of WT medaka using primers mpeg1.2F and mpeg1.2R (Supplementary Table S1). The PCR product was digested with *NotI* and *BamHI* and then ligated in‐frame to a farnesylated mCherry in a pI‐*SceI* plasmid. The plasmid was microinjected into one‐cell stage medaka embryos together with meganuclease *I‐SceI* enzyme as previously described.^(^ ^20^ ^)^ Injected embryos were screened for mCherry‐F transgene expression in macrophages, and stable lines were established and verified by in situ hybridization. For the generation of *tnfa* mutants, guide RNAs (gRNAs) targeting exons 1, 3, and 4 of *tnfa* were designed using CCTop^(^ ^21^ ^)^ and synthesized by IDT (Singapore; Fig. S8; Supplementary Table S1). The three gRNAs were mixed with tracrRNA and HiFi Cas9 protein (IDT) and injected into one‐cell stage medaka embryos. Injected fish and their offspring were genotyped using primers listed in Supplementary Table S1, and stable mutant lines were established.

### Macrophage depletion and drug treatment

For macrophage depletion, *rankl:HSE:cfp/mpeg1:mCherry‐F/ctsk:GFP* embryos at 9 days post fertilization (dpf) were injected intravenously with 15‐nL lipo‐clodronate (5 mg/mL) (Lipo‐Clo) or Lipo‐PBS (Encapsula NanoSciences, Brentwood, TN, USA). Macrophage‐ablated embryos were selected at 10 dpf based on the reduced fluorescence signal of mCherry‐F reporter. For inhibition of *tnfa* expression, RANKL‐induced embryos after heat‐shock were immediately transferred to fish medium (30% Danieau's solution containing 19.3mM NaCl, 0.23mM KCl, 0.13mM MgSO_4_, 0.2mM Ca(NO_3_)_2_, 1.7mM HEPES, pH 7.0) supplemented with 200μM PTX (Sigma‐Aldrich, St. Louis, MO, USA) or 0.05% DMSO as control. The medium was changed daily with fresh drug added for the whole course of the experiments.

### In situ hybridization

Sense and antisense riboprobes spanning 790 nucleotides of the *mpeg1* cDNA (nt1029‐nt1818; ENSORLT00000006101.2; the Ensembl database project, http://www.ensembl.org/) were synthesized using a DIG RNA labeling kit (Roche Diagnostics, Mannheim, Germany). In situ hybridization was performed as previously described.^(^ ^18^ ^)^

### Bone staining

Alizarin Red staining of mineralized bone matrix in fixed embryos was performed as previously described.^(^ ^22^ ^)^ Briefly, larvae at 3 days post heat‐shock (dphs) were fixed with 4% paraformaldehyde, washed three times with PBS solution plus Tween‐20 (PBST), incubated for 15 min in 0.5% potassium hydroxide (KOH), then transferred to 0.001% Alizarin Red solution in 0.5% KOH for at least 4 hours with agitation. Samples were washed for 2 hours with 0.5% KOH, followed by depigmentation with 2% H_2_O_2_ in 0.5% KOH for 1 hour. Embryos were washed two times with 0.5% KOH and mounted in 100% glycerol for imaging. For live bone staining, embryos were incubated in 0.01% calcein (Sigma‐Aldrich) and kept in the dark for at least 1 hour at 30°C. Embryos were washed twice with fish medium and mounted in low‐melting agarose for imaging.

### Cryosectioning and immunostaining

*rankl:HSE:cfp/ctsk:GFP* transgenic embryos were fixed in 4% paraformaldehyde, washed with PBST, and mounted in 1.5% low‐melting agarose in 5% sucrose. The set agar was kept overnight at 4°C in 30% sucrose before cryosectioning using a Cryostat 1850 (Leica, Wetzlar, Germany). Sections of 20 μm were collected on SuperFrost Plus slides (Thermo Fisher Scientific, Waltham, MA, USA) and dried at room temperature. For immunostaining, sections were incubated in 10% goat serum in PBST (3.2mM Na_2_HPO_4_, 0.5mM KH_2_PO_4_, 1.3mM KCl, 135mM NaCl, and 0.05% Tween 20) for 1 hour at room temperature and incubated overnight at 4°C with a 1:100 dilution of cleaved‐caspase‐3 antibody (Asp175; Cell Signaling Technology, Beverly, MA, USA) in blocking buffer. Alexa Fluor 633 IgG goat anti‐rabbit antibody (Invitrogen, Carlsbad, CA, USA) was used as secondary antibody at a 1:500 dilution. Samples were washed with PBS, stained with 4,6‐diamidino‐2‐phenylindole, and kept in Mowiol (Sigma‐Aldrich) until imaging.

### Imaging of live and fixed samples

For live imaging, embryos were anesthetized in 0.016% tricaine (MS‐222; Sigma‐Aldrich) and embedded in 1.2% low melting agarose (Bio‐Rad, USA) in 35 mm glass‐bottom dishes (Eppendorf, Hamburg, Germany), and covered with 2‐mL fish medium containing tricaine. A Nikon SMZ18 epi‐fluorescence microscope was used for capturing whole‐embryo images. Z‐stack images and time‐lapse movies were recorded using a Nikon FV3000 confocal microscope (Nikon, Minato City, Tokyo, Japan) equipped with a ×30/1.05, WD 0.8 silicon oil objective. Time‐lapse files were processed using Imaris (Bitplane AG, Zurich, Switzerland) and Fiji (ImageJ; NIH, Bethesda, MD, USA; https://imagej.nih.gov/ij/) software to generate movies. Z‐stack images were processed into maximum intensity projections and channels merged using Fiji. Alizarin Red‐stained bone samples were imaged using a Nikon Eclipse 90i upright microscope equipped with NIS‐Elements version BR 3.0.

### Cell quantification and statistics

Fluorescent images of whole embryos and confocal z‐stacks were used for the quantification of macrophage and osteoclast numbers as previously described^(^ ^23^ ^)^ with slight modifications. Using Fiji, fluorescent images were converted to greyscale, thresholded, and the area of a cell (averaged from 15 cells from three embryos), as well as the total area of cells in a selected region of interest, were derived. Cell numbers were determined by dividing the total area of cells with the average cell area. For statistical analysis, Student's *t* test (two‐tailed, unpaired) was performed using GraphPad Prism 8.0 (GraphPad Software, La Jolla, CA, USA).

### Fluorescence‐activated cell sorting, RNA sequencing, and bioinformatics analysis

FACS and RNA extraction were performed using previously described protocols^(^ ^24^ ^)^ with some modifications. RNA library construction, Illumina sequencing, and bioinformatics analysis were performed by Novogene (Singapore) following company procedures. Briefly, RANKL expression was induced in *rankl:HSE:cfp/mpeg1:mCherry‐F/ctsk:GFP* transgenic medaka embryos at 9 dpf. At 1 day post heat shock (dphs), 23 larvae per sample were dissociated in 500 μL of 0.25% trypsin EDTA (HyClone; HyClone Laboratories, South Logan, UT, USA) plus collagenase (0.2 mg/mL) at 30°C and pipetted every 10 min for 40 min. The digestion was stopped by adding 50‐μL sterile‐filtered FBS. Cells were washed with PBS supplemented with 2% FBS and resuspended in 400‐μL Leibovitz's L‐15 medium (Gibco; Thermo Fisher Scientific) for FACS using a BD FACSAria II platform (BD Biosciences, San Jose, CA, USA). Fluorescent macrophages and osteoclasts were separated and collected into 400‐μL TRIzol (Thermo Fisher Scientific). RNA extraction was done from three independent biological replicates using PureLink RNA Micro kit (Thermo Fisher Scientific). The RNA quality was determined with an Agilent Bioanalyzer 2100 using Agilent RNA 6000 Pico kit (Agilent Technologies, Santa Clara, CA, USA). Samples with RNA integrity number (RIN) values >7 were submitted for sequencing. Clean reads were aligned to the reference genome for Japanese medaka HdrR, version ASM223467v1 (http://ensembl.org/) using TopHat v2.0.12. Differential expression analysis of two biological replicates per condition was performed using Deseq R package (1.18.0). *P* values were adjusted using Benjamini and Hochberg's approach and genes with *P* values <0.05 were assigned as differentially expressed. Gene ontology (GO) enrichment of differentially expressed genes was analyzed using GOseq R package and only GO terms with a *P* value <0.05 were considered significantly enriched. ShinyGO V0.61 was used to analyze networks of functional groups of up‐ and downregulated genes. Input data were matched to human genes, the *P‐*value cutoff was 0.05, and the 30 most significant terms were used (http://bioinformatics.sdstate.edu/go/).

### Phylogeny and synteny analysis

Amino acid sequences were retrieved from the National Center for Biotechnology Information (NCBI; www.ncbi.nlm.nih.gov/) and ENSEMBL (www.ensembl.org/Multi/Tools/Blast) databases using BLAST searches with default parameters. Sequences were aligned using Clustal W^(^ ^25^ ^)^ and trees were constructed using the maximum likelihood method in MEGA version X^(^ ^26^ ^)^ with the JTT model for aa substitutions and 1000 bootstrap iterations. Conserved synteny analysis was done using GENOMICUS version 100.01 (https://www.genomicus.biologie.ens.fr/genomicus-100.01/cgi-bin/search.pl).^(^ ^27^ ^)^

### Quantitative polymerase chain reaction

For whole embryos, RNA was isolated from 10 embryos per sample using NucleoSpin RNA kit (Macherey‐Nagel, Duren, Germany), and total mRNA was reverse transcribed using the RevertAid First Strand cDNA Synthesis kit (Thermo Fisher Scientific). For RNA isolation from FAC‐sorted macrophages and osteoclasts, the NucleoSpin RNA XS kit (Macherey‐Nagel) was used. RNA isolated from three independent biological samples was converted into cDNA and preamplified using Fluidigm Reverse Transcription Master Mix and Preamp Master Mix, respectively (Fluidigm, South San Francisco, CA, USA). All steps were performed following the manufacturers' standard protocols. PowerUp SYBR Green Master Mix (Applied Biosystems, Foster City, CA, USA) was used for qPCR conducted in a CFX96 Touch system (Bio‐Rad Laboratories, Hercules, CA, USA). Data analysis was performed using Bio‐Rad's CFX Maestro 1.0 software with β‐actin as the loading control for normalization. Two‐tailed Student's *t* tests were performed for statistical analysis.

## Results

### RANKL triggers macrophage recruitment, proliferation, and differentiation at bone matrix

**Fig 1:**
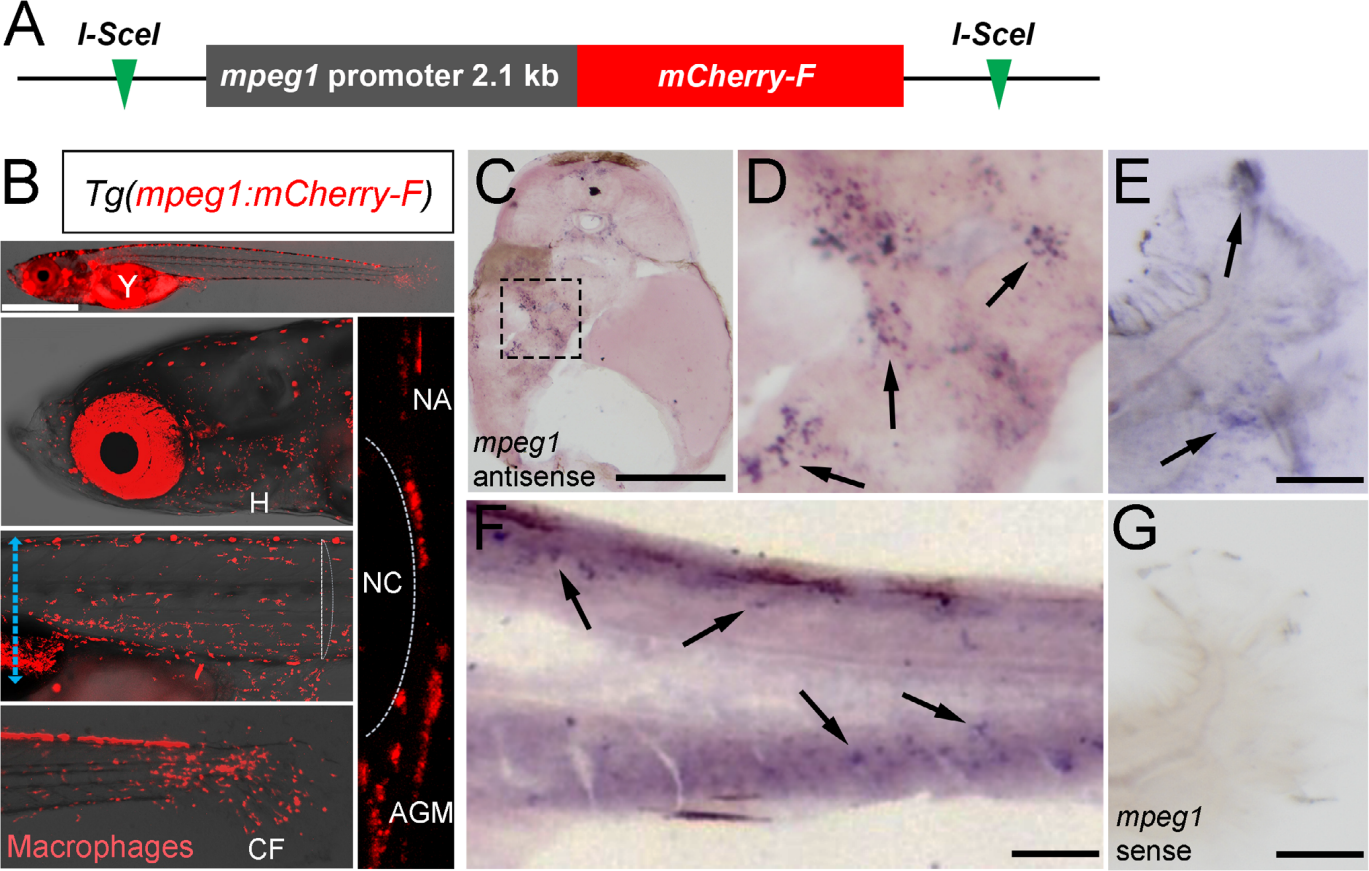
Generation of the *mpeg1:mCherry‐F* reporter line. (*A*) A farnesylated mCherry reporter is driven by a 2.1 kb *mpeg1* promoter and flanked by *I‐SceI* sites. (*B*) At 10 days postfertilization (dpf), *mCherry‐F–*labeled macrophages are distributed throughout the body except for the notochord and spinal cord. (*C–F*) In situ hybridizations of 10 dpf embryos with a *mpeg1* antisense probe on the transverse section of the anterior region (blue dotted line in B) (*C*), and a longitudinal section around the AGM‐CHT area (*F*). D shows high magnification of liver tissue boxed in C, whole‐mount fin region (*E*) arrows point to Mpeg1 positive signals; patterns are similar to the mCherry reporter signals. (*G*) In situ hybridizations with a *mpeg1* sense probe on a control whole‐mount fin. NA = neural arch; NC = notochord; AGM = aorta–gonad–mesonephros; CHT = caudal hematopoietic tissue; CF = caudal fin; H = heart. Scale bar: 1 mm (in *B*); 200 μm (in *C*); 100 μm (in *E–G*).

**Fig 2:**
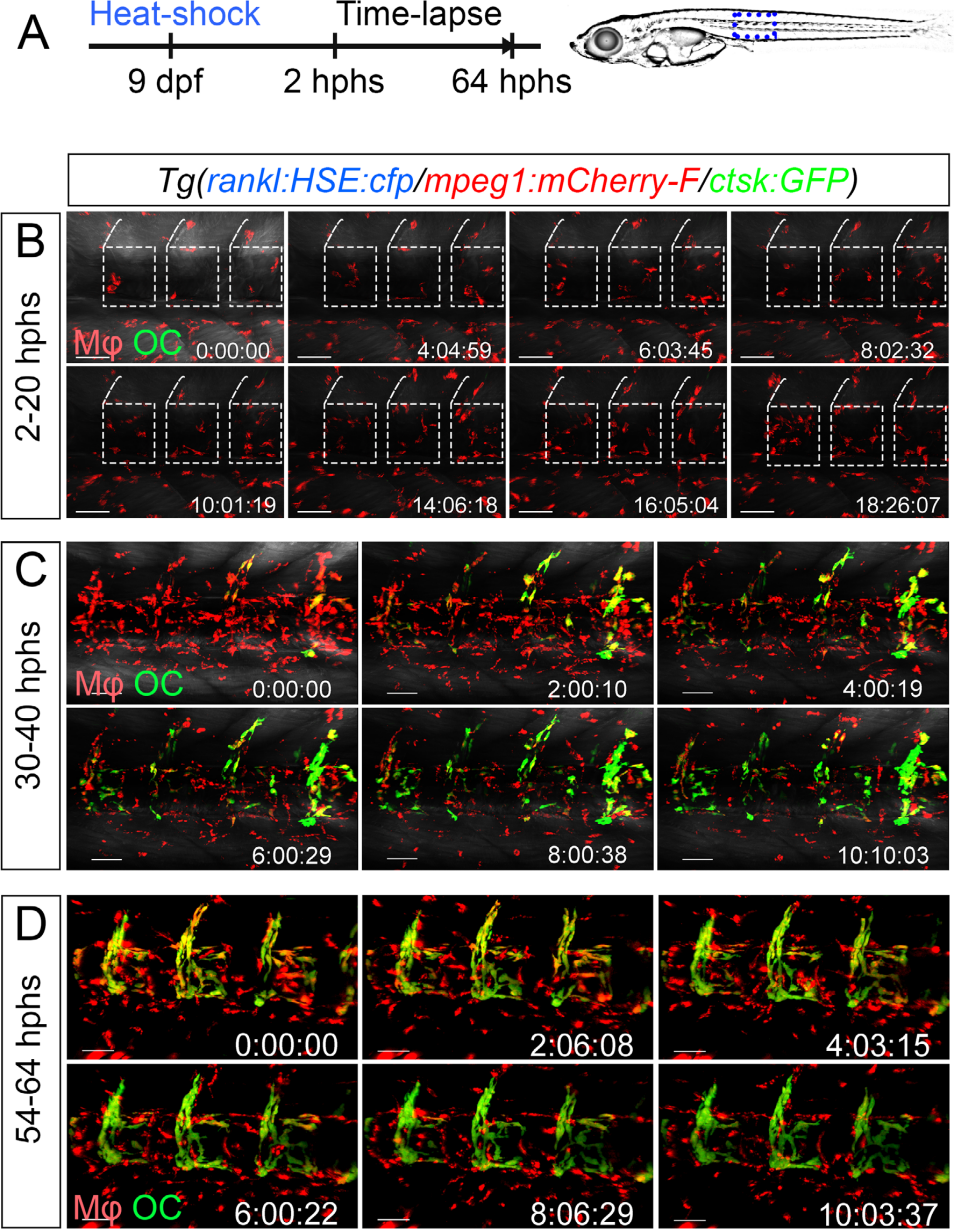
Macrophage recruitment and differentiation into osteoclasts in the medaka vertebral column. (*A*) Experimental scheme. *rankl:HSE:cfp/mpeg1:mCherry‐F/ctsk:GFP* transgenic medaka embryos were heat‐shocked to induce RANKL expression and subjected to confocal time‐lapse imaging. (*B–D*) Still images extracted from movies recorded during the indicated time frame (hours post heat‐shock, hphs). Macrophages are recruited towards the vertebral column as early as 2 hphs and begin to differentiate into *ctsk* osteoclasts around 18 hphs, starting at the anterior region of the vertebral column. At 40 hphs, most of the recruited macrophages have differentiated into osteoclasts, while later recruited macrophages are located around the vertebral column to perform phagocytosis of dying cells. Blue dotted box in (*A*) shows imaged area shown in (*B*–*D*); white dotted boxes in (*B*) depict vertebral bodies. Mφ = macrophage; OC = osteoclast. Time: hh:mm:ss. Scale bar: 50 μm.

To study the dynamic behavior of macrophages by live imaging in medaka, we generated a reporter line expressing farnesylated mCherry under control of the medaka *mpeg1* promoter (Fig. 1*A*). At 10 dpf, *mpeg1:mCherry‐F* positive macrophages were found throughout the body with increased numbers in heart, liver, aorta‐gonad‐mesonephros (AGM) and caudal fin (Fig. 1*B*). The *mpeg1* transgenic line faithfully recapitulated endogenous *mpeg1* transcription as confirmed by RNA in situ hybridization (Fig. 1*C*–*G*). Next, *mpeg1:mCherry‐F* reporter fish were crossed with *rankl:HSE:cfp/ctsk:GFP* transgenic fish, which harbor a RANKL transgene under control of a bidirectional heat‐shock promoter that also drives cyan fluorescent protein (CFP) expression, and express green fluorescent protein (GFP) in osteoclasts under control of the *ctsk* promoter (for details, see To and colleagues^(^ ^18^ ^)^). Confocal time‐lapse imaging showed that in the absence of ectopic RANKL expression (–RANKL), the majority of *mpeg1:mCherry‐F*‐positive macrophages reside within the AGM with only few migratory cells found in other regions of the body, including the vertebral column (Supplementary Fig. S1 *A*; Movie S1). Shortly after RANKL induction (4 hours post heat‐shock [hphs]), *mpeg1*‐positive macrophages started to accumulate in the vertebral column (Fig. 2*A*,*B*). Time‐lapse analysis revealed that the majority of *mpeg1* cells in the vertebral column originated from the AGM, but also cells from other regions were recruited (Supplementary Fig. S1). Macrophages were almost exclusively found at the mineralized matrix of vertebral bodies, but not in the nonmineralized intervertebral discs (Fig. 2*B*; Supplementary Fig. S1 *A*; Movie S2 **)**. In vertebral bodies, macrophages were in close contact with osteoblasts and osteoblast progenitors, which line the bone surface (Supplementary Movie S7). At 30 to 64 hphs, the recruited macrophages gradually differentiated into *ctsk*‐positive osteoclasts (Fig. 2*C*,*D*; Supplementary Movies S3 and S4).

Starting from 35 hphs, the first osteoclasts started to undergo cell death and became cleared by newly recruited phagocytic macrophages (Supplementary Fig. S2; Movies S3 and S4). Quantification of *mpeg1:mCherry‐F* and *ctsk:GFP* cells showed high numbers of recruited macrophages and ectopic osteoclasts along the vertebral column of RANKL‐induced embryos compared with controls (Supplementary Fig. S3). Osteoclast numbers gradually declined after 2 dphs, while new macrophages continued to migrate towards the vertebral column over the course of 1 week (data not shown). The total number of macrophages increased partly by steady‐state hematopoiesis, but also because of cell proliferation that occurred both in the AGM and around the vertebral column (Supplementary Fig. S3; Movie S7). Our findings suggest that in medaka ectopic RANKL expression triggers the activation of macrophages, stimulates their recruitment towards mineralized matrix in a directed manner, and promotes their differentiation into osteoclasts.

### Macrophages reduce their dynamics during osteoclast maturation

To quantitate the dynamics of macrophages during osteoclast differentiation, we analyzed time‐lapse movies of RANKL‐induced macrophages and osteoclasts recorded before and after differentiation. In an early phase at 4 hphs, macrophages in RANKL‐induced embryos exhibited higher speed and displacement when compared with –RANKL controls. A more angular and less spherical cell morphology was observed in +RANKL macrophages, indicating their motile state (Supplementary Fig. S1 *A*). However, at 34 hphs, the motility of differentiating macrophages became reduced as they started to express *ctsk:GFP*, while undifferentiated macrophages remained highly mobile (Supplementary Fig. S1 *B*; Movie S4). This suggests that the reduction of macrophage dynamics is a prerequisite for osteoclast differentiation, possibly to allow tighter attachment of forming osteoclasts to bone matrix for subsequent cell fusion to generate multinucleated osteoclasts.

### Depletion of macrophages prevents osteoclast formation

**Fig 3:**
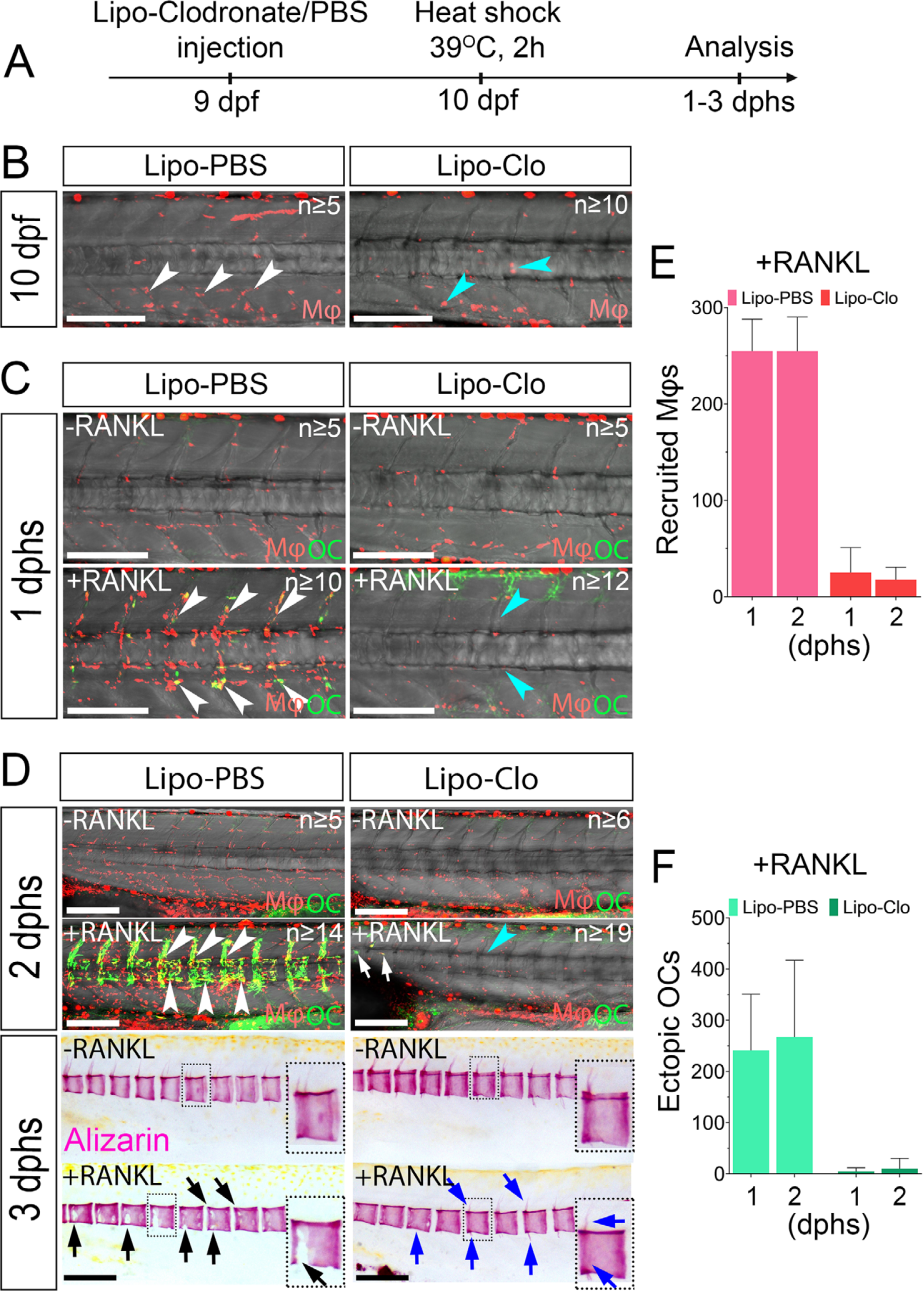
Macrophage depletion prevents osteoclast formation. (*A*) Experimental scheme. *rankl:HSE:cfp/mpeg1:mCherry‐F/ctsk:GFP* transgenic embryos are injected with lipo‐clodronate or Lipo‐PBS at 9 days postfertilization (dpf). At 10 dpf, embryos with efficient macrophage depletion were selected based on the quantity and morphology of mCherry signals, and then subjected to RANKL induction. (*B*) Lipo‐clodronate (Lipo‐Clo) injection causes macrophage death at 1 day after injection (round cells pointed by cyan arrowheads), while Lipo‐PBS injected embryos show normal macrophage morphology and distribution (white arrowheads). (*C*) Lipo‐PBS injected control embryos show normal macrophage recruitment and osteoclast differentiation at 1 day post heat‐shock (dphs; white arrowheads). In macrophage‐ablated embryos, osteoclast formation along the trunk is notably inhibited (cyan arrowheads). (*D*) At 2 dphs, abundant osteoclasts have formed in Lipo‐PBS–injected embryos (white arrowheads), while only single osteoclasts are seen in the anterior region of Lipo‐Clo–injected embryos (white arrows). Alizarin Red staining of mineralized bone matrix at 3 dphs reveals normal bone development in both Lipo‐PBS and Lipo‐Clo–treated embryos in the absence of RANKL induction. Upon RANKL induction, control larvae have severe lesions in the mineralized matrix (black arrows), while Lipo‐Clo–injected embryos show minor or no defects (blue arrows). Insets show individual vertebral bodies at higher magnification. (*E*,*F*) Quantification of cell numbers shows efficient depletion of macrophages and inhibition of osteoclast formation in Lipo‐Clo–treated embryos. N_Lipo‐PBS larvae_ = 5–14, N_Lipo‐Clo larvae_ = 6–19, from three independent experiments. Mφ = macrophage; OC = osteoclast. Scale bar: 200 μm.

As in mammals^(^ ^3^ ^)^, medaka macrophages also differentiate into osteoclasts. We next determined whether osteoclasts also form in the absence of macrophages, indicating possible alternative cellular sources. We depleted macrophages by Lipo‐Clo injection into medaka embryos at 9 dpf and induced RANKL expression by heat‐shock at 10 dpf. In the absence of RANKL induction, Lipo‐Clo treatment did not cause any obvious changes to bone development (Fig. 3*A*–*D*). After RANKL induction, at 1 and 2 dphs, Lipo‐PBS control‐injected embryos showed abundant macrophage recruitment and osteoclast formation along the vertebral column as expected (Fig. 3*C*–*F*). In contrast, Lipo‐Clo treatment resulted in a 75% ablation of macrophages (Fig. 3*C*–*E*). Only a few macrophages were recruited to the vertebral column, and osteoclast formation was strongly impaired (Fig. 3*C*–*F*; Supplementary Movies S5 and S6). The effect of macrophage depletion on bone protection was then assessed using Alizarin Red staining at 3 dphs. After RANKL induction, the bone matrix of Lipo‐Clo–injected embryos was efficiently protected with only few minor defects, which was in stark contrast to the severe lesions formed along the vertebral columns of Lipo‐PBS–injected embryos (Fig. 3*D*). This observation indicates that the integrity of a macrophage population is important for osteoclast formation.

### Transcriptome profiling reveals similarities between medaka and human osteoclasts

The behavior and dynamics of macrophages change rapidly during differentiation into osteoclasts ([Link], [Link]). To identify molecular signatures underlying these changes, we performed RNAseq analysis and compared transcriptome profiles of FACS‐purified macrophages (*mpeg1* ^*+*^/*ctsk* ^*−*^cells) with those of osteoclasts (*mpeg* ^*+*^/*ctsk* ^*+*^ double‐positive cells) at 1 dphs. Analysis of differentially expressed genes (DEGs) revealed gene sets that are similarly regulated in human and mouse osteoclastogenesis. Conventional markers of osteoclastogenesis in mammals such as *trap*, *ctsk*, *siglec15*, *nfatc1*, *tgfb1*, and *dap12* were highly upregulated in *mpeg* ^*+*^/*ctsk* ^*+*^ double‐positive medaka cells, similar to the situation in human and mouse osteoclasts (Table 1). Our RNAseq datasets also indicated that genes encoding the medaka chemokine/cytokine receptors Cx3cr1, Il22ra2a, and Il22ra2b were downregulated in medaka osteoclasts, but significance could only be validated by qPCR analysis for *il22ra2b* (Supplementary Figs. [Link], [Link]; Table 1). The downregulation of cytokine receptor genes in our medaka model suggests a possible reduction of the motility of RANKL‐induced macrophages as they differentiate into osteoclasts.

**Table 1:** Selected Regulated Medaka Genes With Orthologs Involved in Mammalian Osteoporosis and Bone Loss

Gene	Description	Reads Mφ	Reads OC	Log2 FC	*p* adj value	Known function in mammalian bone	Ref
*trap*	Acid phosphatase 5a, tartrate resistant	1766	62181	5.1	0.000	Modulation of osteoclast adhesion and resorption	^(^ ^65^ ^)^
*siglec15*	Sialic acid binding Ig like lectin 15	131	3833	4.9	0.000	Osteoclast differentiation	^(^ ^66^ ^)^
*nfatc1*	Nuclear factor of activated T cells 1	139	3218	4.5	0.001	Osteoclast fusion	^(^ ^67^, ^68^ ^)^
*a‐sdf1a/cxcl12*	Stromal cell‐derived factor 1a	61	1233	4.3	0.001	Osteoclast precursor recruitment	^(^ ^69^ ^)^
*mcoln3a*	Mucolipin 3a	72	1276	4.2	0.050	Triggering Ca^2+^ release and influx during osteoclastogenesis and bone remodeling	^(^ ^70^ ^)^
*angptl7*	Angiopoietin‐like 7	176	3074	4.1	0.000	Osteoclast stimulation.	^(^ ^71^, ^72^ ^)^
*tspan5*	Tetraspanin 5	137	1213	3.2	0.043	Control of osteoclast fusion	^(^ ^73^ ^)^
*dap12/ tyrobp*	dap12/TYRO protein tyrosine kinase binding protein	642	4642	2.9	0.000	Osteoclast differentiation and function	^(^ ^74^ ^)^
*LOC101158504*	C‐X‐C motif chemokine 9	520	2681	2.3	0.01	Produced by osteoblasts and osteoclasts; induces osteoclast migration and adhesion.	^(^ ^75^ ^)^
*ctsk*	Cathepsin K	53392	164567	1.6	0.000	Protease for osteoclast resorptive activity	^(^ ^76^ ^)^
*ckba*	Creatine kinase B	4260	10737	1.3	0.025	Osteoclast resorption; crucial for Actin ring formation and bone resorption.	^(^ ^77^ ^)^
*gsna*	Gelsolin a	7076	16649	1.2	0.016	Assembly/disassembly of Actin filaments in osteoclast podosomes; cell migration	^(^ ^78^ ^)^
*LOC101163603*	C‐C chemokine receptor type 1	341	0	inf	0.020	Osteoclast recruitment, cell motility	^(^ ^79^ ^)^
*socs1b*	Suppressor of cytokine signaling 1b	348	1	−9.4	0.020	Osteoclast formation by cytokine modulation	^(^ ^80^ ^)^
*sh2dp1*	SH2 domain‐containing protein 1B	511	3	−7.4	0.010	Control of osteoclast resorptive activity and fusion	^(^ ^81^ ^)^
*fes*	FES proto‐oncogene, tyrosine kinase	823	20	−5.4	0.000	Osteoclast differentiation	^(^ ^82^ ^)^
*LOC105355996*	Interleukin‐20 receptor subunit alpha	3043	205	−3.9	0.000	Osteoclast differentiation	^(^ ^28^ ^)^
*sbno2*	Strawberry notch homolog 2	1559	188	−3.1	0.020	Osteoclast fusion	^(^ ^83^ ^)^
*pip5k1ba*	Phosphatidylinositol‐4‐phosphate 5‐kinase, type I, beta a	2591	427	−2.6	0.000	Modulation of osteoblast and osteoclast differentiation	^(^ ^84^ ^)^
*grna*	Granulin a	17770	7107	−1.3	0.010	Osteoclast formation	^(^ ^85^ ^)^
*coro1a*	Coronin, Actin binding protein, 1A	29530	13890	−1.1	0.020	Regulation of lysosomal secretion of cathepsin K	^(^ ^86^ ^)^

Abbreviations: Mφ: macrophage, OC: osteoclast, FC: fold change.

### RANKL‐induced macrophages acquire a bone‐remodeling signature

**Fig 4:**
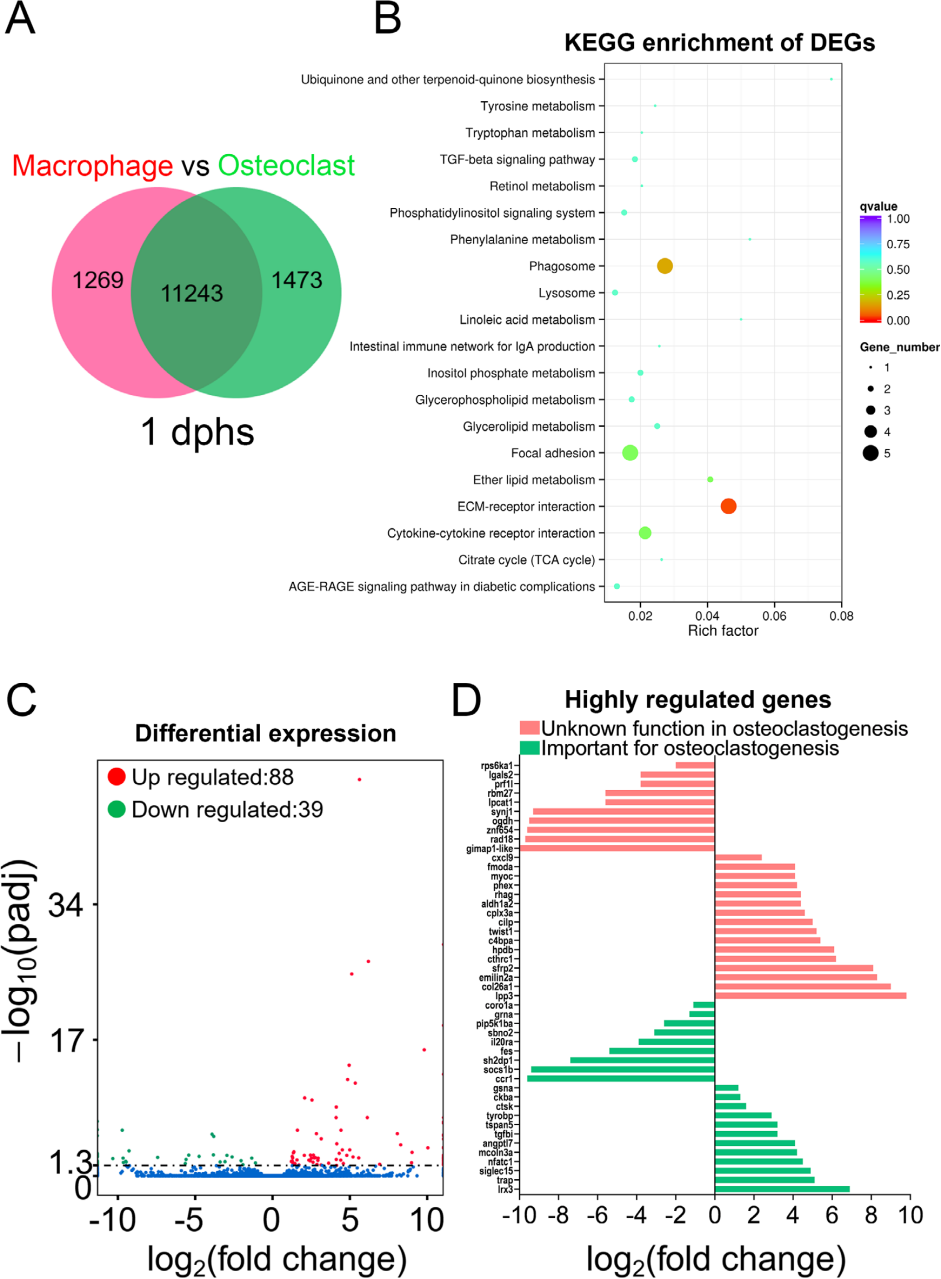
Transcriptome profiles of medaka macrophages and osteoclasts. (*A*) Venn diagram showing the total number of genes expressed in macrophages and osteoclasts at 1 day post heat‐shock (dphs), with the overlapping region showing commonly expressed genes. (*B*) KEGG pathway enrichment analysis of differentially expressed genes (DEGs) showing significantly enriched categories including phagosome, focal adhesion, cytokine–cytokine receptor interaction, and extracellular matrix‐receptor interaction. (*C*) Volcano plot showing up‐ and downregulated genes in osteoclasts compared with macrophages with adjusted *p* values <0.05 (*p* adj value <0.05). Insignificantly regulated genes are shown as blue dots. (*D*) List of genes with the highest regulation indicated by log_2_‐fold change; all genes have adjusted *p* values <0.05.

Our DEG analysis identified more than 1,000 genes that were either commonly or uniquely expressed in macrophages before and after differentiation into osteoclasts (Fig. 4*A*). Of the commonly expressed genes, 88 genes were significantly upregulated and 39 genes downregulated with adjusted *P* values of <0.05 (Fig. 4*A*,*C*). A KEGG analysis of DEGs revealed an enrichment of the terms phagosome, focal adhesion, extracellular membrane‐receptor interaction, and cytokine‐cytokine receptor interaction (Fig. 4*B*). Apart from genes for which the corresponding human orthologs have important functions in osteoclastogenesis, such as *fes*, *sh2dp1*, *socs1b*, *tspan5*, and *mcoln3a* (Fig. 4*D*; Table 1), we observed other highly regulated genes with so far uncharacterized functions in osteoclastogenesis. This includes upregulation of *emilin2a*, which is known to block cancer cell proliferation;^(^ ^29^ ^)^ and *sfrp2*, which controls normal osteoblast differentiation and exerts an antiapoptotic effect.^(^ ^30^, ^31^ ^)^ Among downregulated genes, we identified *gimap1‐like*, which is essential for development of T and B lymphocytes; *rad18*, which is a key player in controlling DNA damage tolerance^(^ ^32^, ^33^ ^)^; and *ogdh*, which is involved in glucose oxidation and cancer cell viability^(^ ^34^, ^35^ ^)^ (Fig. 4*D*). Next, we performed a GO analysis using ShinyGO v0.61. Individual lists of up‐ and downregulated genes were converted into human gene IDs and UniProtKB IDs using BioMart (Ensembl.org). UniProtKB IDs were later used as input for the analysis.

The analysis of upregulated genes surprisingly showed a strong enrichment of pathways involved in chondrocyte and cartilage development, bone remodeling, and osteoblast differentiation. This included the collagen‐coding genes *col6a2*, *col12a1*, as well as the secreted osteoblast differentiation inducers *tgfb1* and *sfrp2* (Supplementary Fig. S4, Table 2).^(^ ^36^ ^)^ Consistent also with earlier studies that reported expression of collagen genes in macrophages,^(^ ^37^, ^38^, ^39^ ^)^ qPCR analysis confirmed the upregulation of *col6a2* and *col12a1*, as well as integrin subunit beta 3 (*itgb3*) in RANKL‐induced macrophages and validated our RNAseq analysis. Interestingly, also genes encoding the respective complexing subunits, ie, *col6a1*, *col6a3*, *itga5*, and *itga2.2*, were upregulated (Fig. S5 *B*).

**Table 2:** Upregulation of Osteoblast/Chondrocyte Promoting Genes in Medaka Osteoclasts

Gene	Description	Reads Mφ	Reads OC	Log2 FC	*p* adj value	Protein localization	Role in bone/cartilage formation	Ref #
*itgb3*	Integrin subunit beta 3	0	3016	inf^a^	0.000	Transmembrane	Corticalization for bone growth	^(^ ^87^ ^)^
*col6a2*	Collagen alpha‐2(VI) chain	0	880	inf	0.000	Secreted	Chondrocyte proliferation and cartilage generation	^(^ ^88^ ^)^
*foxc1*	Forkhead box C1‐A	0	362	inf	0.007	Nuclear	Osteoprogenitor proliferation; osteogenesis	^(^ ^89^ ^)^
*sfrp2*	Secreted frizzled related protein 2	3	854	8	0.000	Secreted	Osteogenic differentiation	^(^ ^90^ ^)^
*cthrc1*	Collagen triple helix repeat containing 1	90	6684	6.21	0.000	Secreted	Osteoblast proliferation, bone cell coupling	^(^ ^91^ ^)^
*myoc*	Myocilin	61	1070	4.14	0.009	Secreted	Osteoblast differentiation; bone remodeling	^(^ ^92^ ^)^
*col12a1*	Collagen alpha‐1(XII) chain	83	1038	3.64	0.027	Secreted	Osteoblast polarity, bone matrix	^(^ ^93^ ^)^
*tgfb1*	Transforming growth factor beta 1	367	3291	3.16	0.000	Secreted	Mesenchymal progenitor differentiation; chondrogenesis	^(^ ^94^ ^)^
*thbs1*	Thrombospondin 1	426	2689	2.66	0.003	Secreted	Mesenchymal progenitor differentiation; chondrogenesis	^(^ ^95^ ^)^
*ccn1*	Cellular communication network factor 1	2434	14444	2.57	0.000	Secreted	Chondrocyte maturation	^(^ ^96^ ^)^
*cldn11*	Claudin‐11	440	2538	2.53	0.006	Transmembrane	Osteoblast differentiation via Notch	^(^ ^97^ ^)^

^a^ Infinite.

A larger fraction of downregulated genes was immune‐related and is known to be involved in the activation of the immune response of macrophages (Supplementary Fig. S4 *A*). Our data suggest that macrophages are highly dynamic in regulating their gene expression, not only to favor bone‐resorbing activity, but also potentially to promote bone formation by secreting factors that trigger osteoblast differentiation.

### Macrophage recruitment and differentiation are impaired in *tnfa* mutants

**Fig 5:**
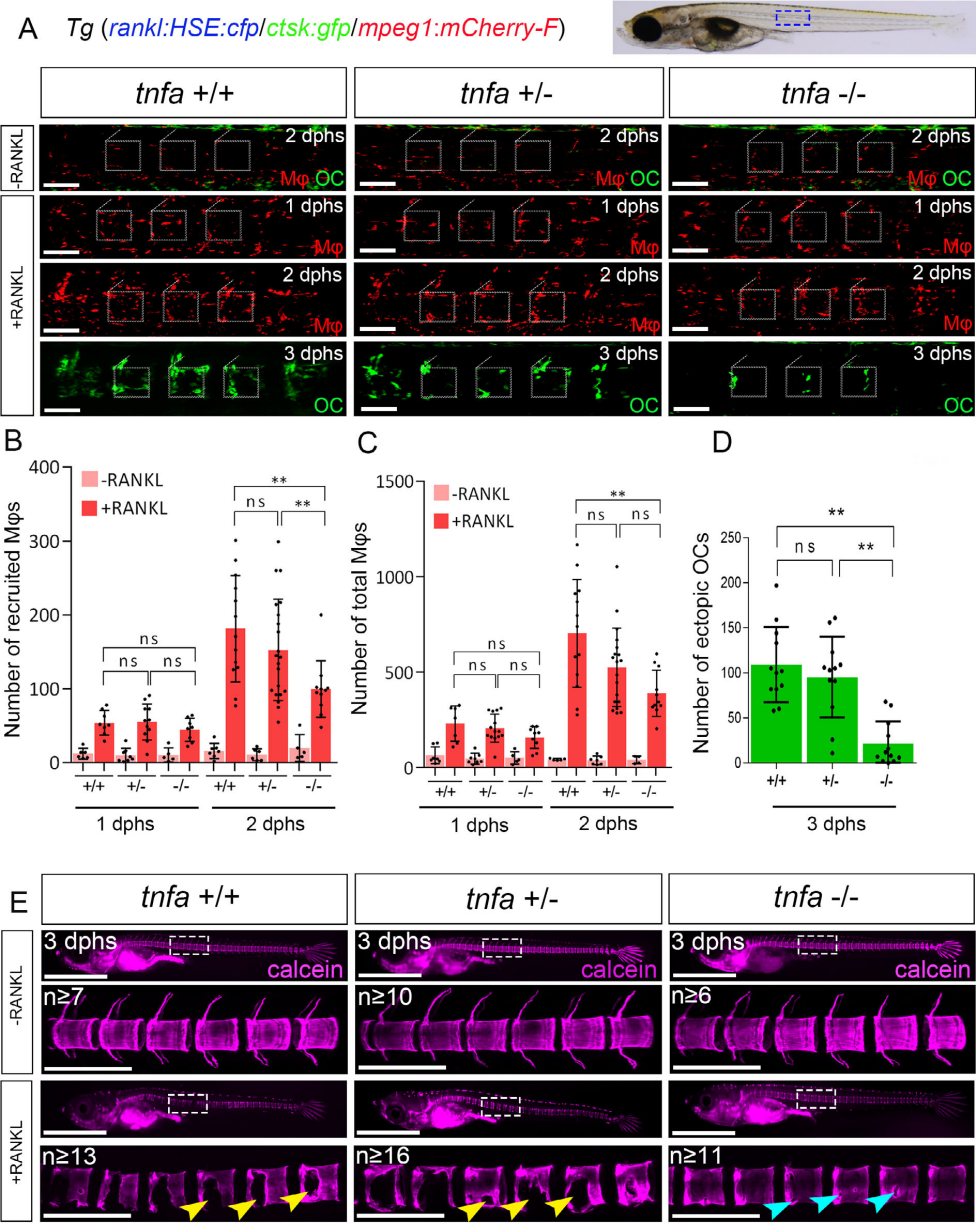
Macrophage recruitment and differentiation are impaired in *tnfa* mutants. (*A*) WT siblings, heterozygous and homozygous *tnfa* mutants with *rankl:HSE:cfp*/*mpeg1:mCherry‐F*/*ctsk:GFP* transgenic background were heat‐shocked at 9 days postfertilization (dpf) for 2 hours. Embryos without *rankl:HSE:cfp* transgene were used as controls (–RANKL). Blue box on bright field image depicts area imaged. White boxes encircle individual vertebrae. In –RANKL embryos, the distribution of macrophages in *tnfa* mutants is not different from that in WT siblings (*tnfa* ^*+/+*^). Upon RANKL induction (+RANKL), there are less macrophages recruited in *tnfa* ^*−/−*^ embryos compared with the heterozygous or WT siblings. Also, numbers of total macrophages and ectopic osteoclasts in homozygous mutants are significantly less than in WT embryos. (*B*–*D*) Quantification of recruited macrophages, total macrophages, and ectopic osteoclasts, respectively. ns = Not significant. Error bars indicate mean numbers ± SD, **p < 0.01. Student's *t* test (unpaired, two‐tailed). (*E*) Calcein staining shows normal mineralization in *tnfa* ^+/−^ and *tnfa* ^−/−^ mutants and WT siblings in the absence of RANKL induction. After RANKL induction, *tnfa* ^−/−^ mutants exhibit less bone resorption with minor lesions (cyan arrowheads), compared with the severe defects in heterozygous and WT embryos (yellow arrowheads). dphs = days post heat‐shock. Scale bars: 100 μm (in *A*), 1 mm (in *E*, low magnification), 200 μm (in *E*, high magnification).

Treatment of mouse bone marrow‐derived macrophages with the proinflammatory cytokine TNFα in the presence of M‐CSF and RANKL stimulates osteoclast differentiation in vitro.^(^ ^15^, ^16^, ^17^ ^)^ We therefore investigated the requirement of this proinflammatory cytokine for macrophage recruitment and osteoclast differentiation in vivo in the medaka osteoporosis model. First, we performed quantitative PCR analysis to test whether *tnfa* transcription is affected by heat‐shock induction and RANKL overexpression. Compared with non‐heat shocked controls, heat shocked −RANKL embryos showed a significant reduction of *tnfa* expression at 1, 2, and 3 dphs (Supplementary Fig. S8 *A*). This is consistent with a temperature‐induced reduction of *Tnfa* transcription in mouse macrophages.^(^ ^40^ ^)^ Importantly, however, RANKL induction (+RANKL) resulted in a significant upregulation of *tnfa* transcription suggesting that *tnfa* is involved in the osteoporotic response. To test this, we generated a *tnfa* medaka mutant using CRISPR/Cas9 (Supplementary Fig. S8 *B*–*D*). Similar to *Tnfa* mouse mutants, which show normal development,^(^ ^41^ ^)^ stable CRISPR/Cas9 medaka *tnfa* mutants were viable and developed normally with no obvious alterations (data not shown). Mutants were crossed with *rankl:HSE:cfp/mpeg1:mCherry‐F/ctsk:GFP* transgenic fish, and macrophage behavior and osteoclast formation were tracked by live imaging as described above. In the absence of RANKL induction (–RANKL), *tnfa* mutants exhibited normal distribution and density of macrophages when compared with nonmutant siblings (Fig. 5*A*–*C*). Upon RANKL induction (+RANKL), *tnfa* ^−/−^ mutant macrophages were recruited towards the vertebral column at 1 dphs, similar to WT macrophages. Also, the total number of macrophages was initially similar in both WT and mutant embryos at 1 dphs. At 2 dphs, however, both total and recruited macrophage numbers were significantly reduced in homozygous mutants compared with that of WT siblings (Fig. 5*B*,*C*). Furthermore, *tnfa* ^−/−^ mutants exhibited significantly reduced osteoclast numbers, while osteoclasts were abundant in RANKL‐induced nonmutant siblings, (Fig. 5*A*,*D*). Next, to investigate whether TNFα inhibition is sufficient to protect bone matrix from osteoporotic insult, RANKL‐induced larvae were stained with calcein at 3 dphs to visualize mineralized matrix in the vertebral column. Upon RANKL induction, heterozygous *tnfa* ^*+/−*^ carriers showed similar bone defects as nonmutant siblings, which included bone loss in the neural arches and large lesions in the vertebral centra (Fig. 5*E*). Homozygous *tnfa* ^*−/−*^ embryos, in contrast, had fewer and smaller lesions in the centra indicative of improved bone integrity. This suggests that TNFα is required for macrophage activation and osteoclast formation after RANKL induction in vivo.

**Fig 6:**
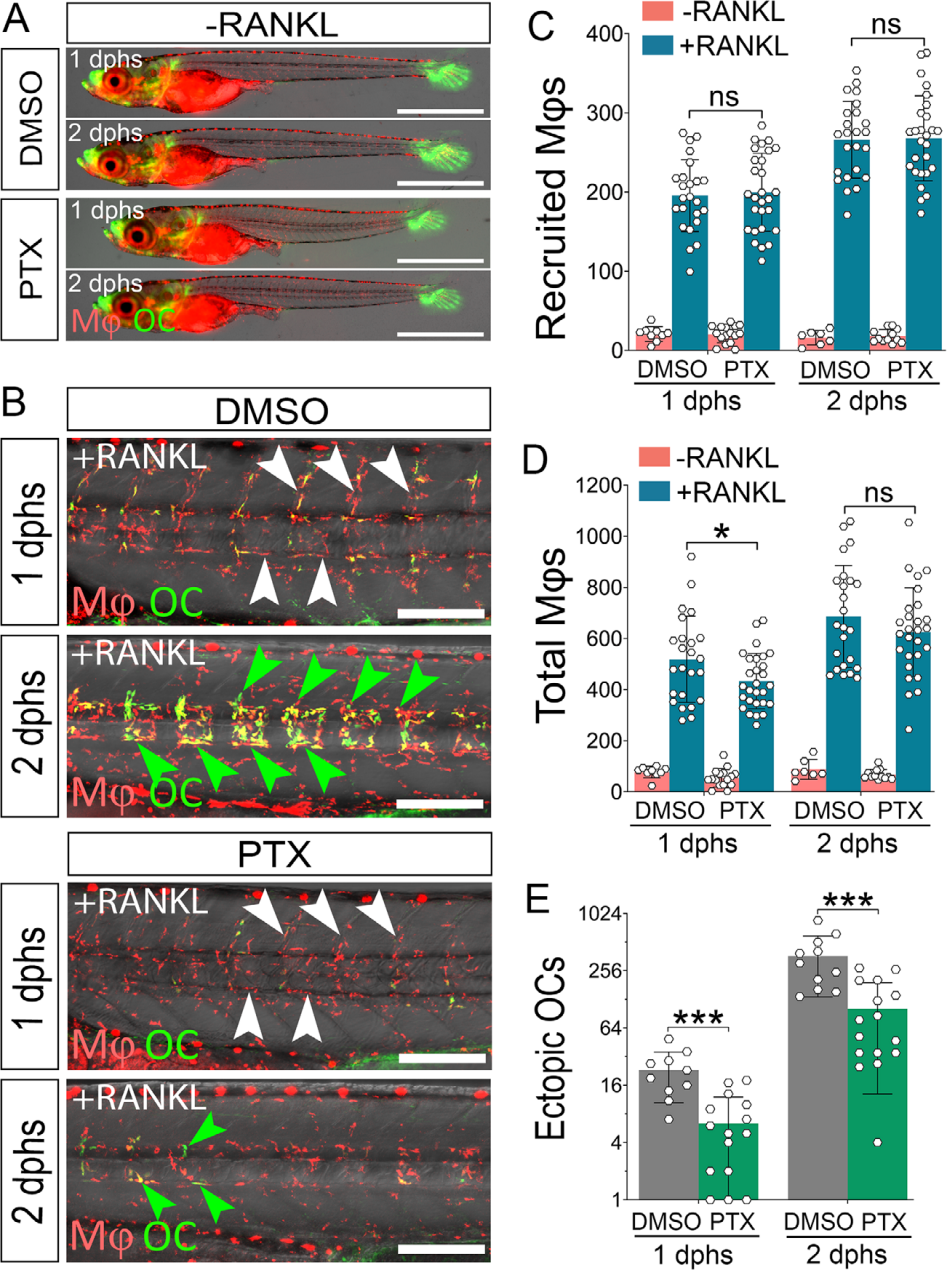
Chemical inhibition of *tnfa* impedes osteoclast formation. (*A*) Heat‐induced RANKL negative *mpeg1:mCherry‐F/ctsk:GFP* transgenic embryos treated with DMSO or pentoxifylline (PTX) showed normal macrophage density and distribution. (*B*) *rankl:HSE:cfp/mpeg1:mCherry‐F/ctsk:GFP* transgenic embryos were heat‐shocked for RANKL induction and immediately treated with PTX or DMSO. Upon RANKL induction, macrophage recruitment was not significantly different in DMSO‐ and PTX‐treated larvae (white arrowheads), but osteoclast formation was strongly impaired in PTX‐treated embryos at 1 and 2 days post heat‐shock (dphs; green arrowheads). (*C*,*D*) Quantification showed that the total number of macrophages at 1 dphs was slightly higher in DMSO control than in PTX treated embryos, but macrophage recruitment was not significantly different in both samples. (*E*) Quantification of ectopic osteoclasts shows significantly less osteoclast formation in PTX treated embryos at 1 and 2 dphs. Quantifications in (*C*–*E*) show mean number of macrophages and osteoclasts ± SD, Student's *t* test (two‐tailed, unpaired), 7≤N_DMSO_RANKL−_≤9, 22≤N_DMSO_RANKL+_≤24, 12≤N_PTX_RANKL−_≤16, 27≤N_PTX_RANKL+_≤28, *p ˂ 0.05, ***p ˂ 0.001, data from three independent experiments. Mφ = macrophage; ns = nonsignificant; OC = osteoclast. Scale bar: 200 μm (in *B*), 1 mm (in *A*).

To confirm this, we finally treated nonmutant embryos with the chemical inhibitor PTX, which inhibits TNFα synthesis. Importantly, in the absence of ectopic RANKL, PTX treatment did not cause any obvious changes to the distribution and density of macrophages (Fig. 6*A*). Moreover, upon RANKL induction, macrophages were recruited to the vertebral column to a similar extent in DMSO‐ and PTX‐treated embryos, as quantitated at 1 and 2 dphs (Fig. 6*B*,*C*; white arrowheads). The total number of macrophages was slightly lower in PTX‐treated embryos at 1 dphs but increased to control levels at 2 dphs (Fig. 6*D*). Thus, other than in *tnfa* ^*−/−*^ mutants, macrophage recruitment was not affected by PTX, which we attribute to a lower efficacy of the drug compared with the genetic deletion. Importantly, however, osteoclast differentiation was strongly impaired in PTX‐treated embryos when compared with DMSO controls at both 1 and 2 dphs (Fig. 6*B*,*E*, green arrowheads). We then analyzed the resorptive activity of RANKL‐induced osteoclasts in control and PTX‐treated embryos using Alizarin Red staining. In the absence of ectopic RANKL, both PTX‐ and DMSO‐treated embryos showed normal mineralization with intact neural arches and vertebral bodies (Supplementary Fig. S9). Upon RANKL induction, DMSO control embryos exhibited resorption of neural arches and severe lesions in the centra. In contrast, PTX treatment resulted in considerable bone protection (Supplementary Fig. S9). Together, our data show that in medaka TNFα is required for macrophage activation and osteoclast formation.

## Discussion

Small teleost fish, such as zebrafish and medaka, are widely used to model human bone diseases given their unique experimental advantages, as well as genetic and cellular similarities of medaka and human bone.^(^ ^42^ ^)^ In the present study, we used live imaging to demonstrate that in medaka, similar to mammals,^(^ ^3^ ^)^ osteoclasts are derived from the monocyte/macrophage lineage in RANKL‐induced conditions. Our macrophage depletion data suggest that in medaka no alternative cellular sources exist for osteoclasts, and that the formation of osteoclasts requires a high integrity of the macrophage population. Transcriptome profiling of medaka macrophages and osteoclasts identified sets of genes that are similarly regulated in human and mouse osteoclastogenesis. It also revealed the induction of genes in differentiated macrophages that are known to be involved in osteoblast formation and bone remodeling. We show that TNFα is essential for macrophage activation and osteoclast differentiation. Together, these results strengthen the medaka as a unique in vivo model accessible to live imaging and suitable for osteoporosis‐related studies.

### Targeted migration and bone cell interaction of macrophages in medaka

Macrophages express membrane‐bound receptors that are needed for their recruitment to target sites in response to pathogens or instructive signals coming from other cell types. After RANKL induction in medaka, macrophages are recruited towards bone matrix, where they interact with osteoblasts and osteoblast progenitors and differentiate into osteoclasts (Fig. 2). Importantly, although massively recruited to the vertebral column, macrophages only accumulate at mineralized matrix of vertebral bodies, but not at the nonmineralized intervertebral discs. This suggests that signals triggering macrophage recruitment could be released either by resident cells lining the bone surface, ie, osteoblasts and osteoblast progenitors, or the matrix itself. Such signals appear to be absent from intervertebral discs or alternatively are distinct from those that induce chronic macrophage recruitment seen in intervertebral disc‐degeneration diseases.^(^ ^43^ ^)^ Previous studies indicated that osteoclasts can form from mouse spleen or bone marrow cells in the absence of bone matrix when cocultured with osteoblasts and supplemented with calcitriol (1 α,25(OH)2 vitamin D3).^(^ ^44^ ^)^ This suggests that ossified matrix is dispensable for osteoclast formation and that osteoblasts instead produce the necessary differentiation signals. However, whether osteoblasts also release signals to recruit macrophages is still unknown. In mammals, osteoblasts express RANKL, which is important for osteoclast differentiation. In the medaka model, however, heat‐shock induced RANKL is ubiquitous, yet only mineralized tissue attracts macrophages. This suggests that ectopic RANKL potentially acts directly on osteoblasts to trigger a release of attractants needed for macrophage recruitment. Consistent with this idea, we recently reported that medaka osteoblasts express the chemokine ligand Cxcl9l, which controls migration of macrophages towards bone matrix.^(^ ^19^ ^)^ Future characterization of osteoblasts is likely to identify additional factors controlling macrophage recruitment.

### Macrophages are the exclusive source for osteoclasts in medaka

Macrophages are highly dynamic and have a variety of functions ranging from the clearance of foreign effectors to mediating communication across different cell types under developmental and pathological conditions.^(^ ^45^, ^46^ ^)^ By time‐lapse analysis, we found that all ectopically induced *ctsk*‐expressing osteoclasts in the vertebral column originate from recruited *mpeg1* macrophages, suggesting that these macrophages are the exclusive cellular source for osteoclasts. Consistently, the transient depletion of *mpeg1* cells by Lipo‐Clo significantly reduced osteoclast numbers upon RANKL induction (Fig. 3). The few remaining osteoclasts that escaped Lipo‐Clo inhibition also originated from macrophages, but not any other cell type. These results appear in contrast to earlier studies that reported persistent osteoclast formation in macrophage‐depleted mice.^(^ ^47^ ^)^ This difference may imply that mice potentially have osteoclast precursors other than macrophages, and that these precursors are absent in medaka. The other possibility, however, is that the macrophage ablation in mice obtained by a tamoxifen‐inducible Cre‐lox apoptosis system was incomplete, allowing remaining macrophages to differentiate into osteoclasts, similar to our findings in medaka. Consistent with our findings, *Csf1r* mouse mutants exhibit a significant reduction of macrophage numbers in different tissues and also a decrease in osteoclast formation.^(^ ^48^, ^49^ ^)^ Thus, other more efficient ablation techniques are needed to confirm that remaining osteoclasts in mice are differentiated from nondepleted macrophages or other cellular sources. In medaka, however, our live‐imaging approach confirmed that macrophages are the exclusive source for RANKL‐induced osteoclasts.

### TNFα is critical for macrophage recruitment and differentiation

Earlier cell culture studies showed that TNFα induces osteoclast formation from mouse bone marrow‐derived macrophages.^(^ ^15^, ^17^ ^)^ Serum levels of TNFa are also increased in humans with low thyroid‐stimulating hormone (TSH) levels and in mice carrying mutations in TSH receptor (*Tshr* ^*−/−*^ *)*.^(^ ^50^, ^51^ ^)^ Both experience low BMD and an increased risk of bone fractures. Interestingly, in *Tshr* ^*−/−*^ mouse mutants, deletion of *Tnfa* partially rescued the low bone mass phenotype.^(^ ^52^ ^)^ Also, blocking TNFα by injecting a binding protein or deletion of *Tnfa* inhibited bone loss induced by ovariectomy.^(^ ^53^, ^54^ ^)^ Conversely, TNFα addition promoted osteoclast survival and prevented apoptosis.^(^ ^55^ ^)^ Thus, these findings in mice strongly suggest an osteoclast‐promoting effect of TNFα. Consistent with this, we showed that in the medaka osteoporosis model, genetic or chemical inhibition of *tnfa* expression reduced osteoclast formation upon RANKL induction (Figs. 5 and 6). In vivo imaging further revealed that in the absence of ectopic RANKL, TNFα deficiency did not cause any obvious effects on macrophage populations. It was only after RANKL induction that *tnfa* mutants exhibited significantly reduced macrophage numbers overall and in the vertebral column. This strongly suggests that TNFα selectively contributes to osteoclast differentiation under osteoporotic conditions, but not in normal development. Consistently, our qPCR analysis revealed that *tnfa* transcription reached a maximum at 1 day after RANKL induction and was then reduced at 2 and 3 dphs (Supplementary Fig. S8). This implies that TNFα may participate in the initial phase of macrophage recruitment. Its high levels at the beginning appear critical for macrophage differentiation into osteoclasts. Blocking TNFα has been an efficient treatment for rheumatoid arthritis, which is characterized by strong inflammation mediated by macrophages.^(^ ^13^ ^)^ Our results suggest that targeting TNFα signaling might be a promising therapeutic strategy for osteoporosis.

### Dynamic transcriptome changes in activated macrophages suggest multiple functions in bone remodeling

A dynamic regulation of cytokines and their receptors is critical for immune cells to function accurately in response to foreign stimuli or microenvironmental changes.^(^ ^56^ ^)^ Uncontrolled overproduction of cytokines can lead to destructive chronic inflammation, which results in severe tissue defects.^(^ ^57^ ^)^ Certain cytokines receptors, such as IL22Ra and IL20R, have been shown to function as drivers for the migration and recruitment of different immune cell types including T and B cells, dendritic cells, and neutrophils.^(^ ^58^, ^59^ ^)^ The expression of these genes is also found upregulated in patients suffering from bone loss caused by rheumatoid arthritis and osteoporosis.^(^ ^60^, ^61^, ^62^ ^)^ Interestingly, our transcriptome profiling of FAC‐sorted cells showed that these genes were downregulated in macrophages undergoing osteoclast differentiation (Supplementary Fig. S5). The reduced cytokine receptor expression coincided with a reduction of macrophage dynamics after they reached the bone matrix and started differentiating. We speculate that it is critical for macrophages to reduce motility before they attach tightly to bone matrix and form actin rings to facilitate bone resorption. Although cytokine receptor expression was downregulated under osteoporotic conditions, pathways involved in chondrocyte and osteoblast differentiation surprisingly were upregulated in RANKL‐induced macrophages (Table 2 and Supplementary Fig. S5). This could reflect a critical role for activated macrophages in bone cell‐coupling: by promoting formation of chondrocytes and osteoblasts to achieve bone homeostasis. Our findings of upregulated *col6a1*, *col6a2*, *col6a3*, and *col12a1b* expression in RANKL‐induced macrophages are suggestive for a role in osteogenesis. Such a role was earlier proposed by Izu and colleagues, who found that collagens VI and XII form a complex to mediate osteoblast interaction during osteogenesis.^(^ ^63^ ^)^ In the medaka model, we also found upregulation of different integrin subunits and other adhesion molecules (Supplementary Fig. S5). This is in line with our observation that RANKL‐induced macrophages interact dynamically with osteoblasts at the bone surface when maturing into osteoclasts (see Supplementary Movie S7). We speculate that these matrix and adhesion proteins are critical for the tight interaction of preosteoclasts with osteoblasts and facilitate osteoclast differentiation. In an alternative scenario, our observation of upregulated osteogenic transcripts in osteoclasts could reflect the uptake of osteoblast‐released exosomes, a process that was recently shown to occur in regenerating zebrafish scales.^(^ ^64^ ^)^ These osteoblast‐derived exosomes could not only contain factors driving osteoclast differentiation, as reported by Kobayashi‐Sun and colleagues,^(^ ^64^ ^)^ but also osteogenic mRNAs. However, we observed that the majority of upregulated osteogenic genes (i) is already expressed at basal levels in noninduced macrophages, and (ii) encode secreted or transmembrane proteins. This observation favors the idea of a non‐cell autonomous role for activated macrophages in chondro‐ and osteogenesis. Clearly, further studies are needed to investigate the complex roles of macrophages in bone remodeling. Together, our study highlights the dynamic nature of macrophages and the complex intercellular interactions implicated in bone homeostasis.

## Disclosure

The authors declare that they do not have any conflict of interest. All data presented in this article will be shared upon request.

## AUTHOR CONTRIBUTIONS

**Quang Tien Phan**: Conceptualized study; performed experiments; analyzed data; prepared figures, wrote and edited manuscript. **Ranran Liu**: Designed and performed experiments; analyzed data; prepared figures, wrote and edited manuscript. **Tan Wen Hui**: Performed experiments; analyzed data; prepared figures and edited manuscript. **Nurgul Imangali**: Planned and performed apoptosis experiments; analyzed data and prepared figures. **Benedict Cheong**: Performed expression analysis; analyzed data. **Manfred Schartl**: Performed phylogenetic and synteny data analysis; wrote and edited manuscript. **Christoph Winkler**: Conceptualized study; analyzed data; prepared figures; wrote and edited manuscript.

### PEER REVIEW

The peer review history for this article is available at https://publons.com/publon/10.1002/jbm4.10409.

## Supporting information


**Supplementary Figure S1** Macrophages become less motile after differentiation into osteoclasts. (*A*) Cell tracking showed that most macrophages remained in the AGM of ‐RANKL embryos. On the contrary, many macrophages were directionally recruited towards bone matrix of the vertebral column upon RANKL induction. The recruited cells clustered mainly at neural arches and vertebral bodies but not in the intervertebral discs. Macrophage dynamics was determined at 4 hphs. (*B*). At 1–2 dphs, macrophages that differentiated into osteoclasts (*mpeg+/ctsk+*) have significantly reduced motility and are confined to bone matrix. Macrophages that were recruited later stayed undifferentiated and remained highly motile. As the cells were more dynamic, they had angular shapes with lower sphericity. In contrast, mature osteoclasts became more static with rounded shapes (high sphericity) after fusion. Macrophages dynamics was determined at 34 hphs. Student's *t* test was used to determine the significance of difference between groups, error bars show standard deviation. AGM: aorta gonad mesonephros; V: vertebral column; iv: intervertebral disc; vb: vertebral body; na: neural arch; Mφ: macrophage; OC: osteoclast. Scale bar: 100 μm.

**Supplementary Figure S2** Macrophages clear apoptotic osteoclasts. (*A*) Still images from a time‐lapse movie recorded at 30 hphs. Circles show that apoptotic osteoclasts (green cells) at the neural arch are engulfed and digested by conventional phagocytosing macrophages. Time:hh:mm. (*B*) Immunostaining of cleaved‐Caspase‐3 on cryosections revealed apoptosis (white arrowheads) of osteoclasts in RANKL‐induced embryo. Scale bar: 20 μm (in *A*), 40 μm (in *B*).

**Supplementary Figure S3** Quantification of macrophage and osteoclast numbers. (*A*) *rankl:HSE:cfp/mpeg1:mCherry‐F/ctsk:GFP* and *mpeg1:mCherry‐F/ctsk:GFP* transgenic embryos were heat‐shocked and imaged repeatedly at 1, 2 and 3 dphs. In the absence of ectopic RANKL expression, macrophages were distributed at different positions along the body, predominantly in the AGM (white arrowheads). There were no ectopic osteoclasts in the trunk (magenta arrowheads). Upon RANKL induction, macrophages were directionally recruited towards the vertebral column, particularly to the neural arches and the vertebral bodies (cyan arrowheads). Osteoclasts were later formed by differentiation of recruited macrophages along the trunk (yellow arrowheads). The ectopic osteoclasts started to vanish at 3 dphs while new macrophages continued to be recruited. (*B*) Quantification showed mean numbers of recruited macrophages, total macrophages and ectopic osteoclasts ± SD; Student's *t* test (two‐tailed, unpaired), **p˂0.01, ****p˂0.0001, ns: non‐significant, 10 ≤ N_Larvae_ ≤ 19, data from three independent experiments. Mφ: macrophage; OC: osteoclast. Scale bar: 1 mm.

**Supplementary Figure S4** Gene Ontology analysis of up‐ and downregulated genes in osteoclast compared with macrophage. (*A*,*B*) Functional groups in down‐ and upregulated genes, respectively. Listed genes were used as input for GO terms analysis using ShinyGO. Upregulated genes involved in osteoblast differentiation and bone remodeling are labeled in blue, and genes related to adhesion were underlined (*B*).

**Supplementary Figure S5** qPCR validation of gene expression in FAC‐sorted macrophages and osteoclasts. Gene expression was determined at 1 dphs. (*A*) Among downregulated genes, *il22ra2a*, *il22ra2b* and *pip5k1ba* showed trends of downregulation in osteoclasts compared with macrophages, consistent with RNAseq data. In contrast, *cx3cr1* and *fes* show upregulation, while *socs1b*, *sh2dp1* and *sbno2* were not regulated. (*B*) Among upregulated genes, genes encoding collagen VI, collagen XII and integrin subunit beta 3 and their binding subunits were significantly higher expressed in osteoclasts, consistent with RNAseq data. Similarly, expression of osteoclast makers *nfatc1*, *trap* and *ctsk* were also upregulated. Error bars indicate mean fold change ± SD, *p < 0.05, **p < 0.01, ***p < 0.001, ns: non‐significant, Student's *t* test, data were obtained from three biological replicates with three technical repeats each and β‐actin was used as loading control.

**Supplementary Figure S6** Phylogeny analysis of CC chemokine receptors. A molecular phylogeny was computed for all so far identified medaka Ccr receptors as well as human CCRs 1–9, human CX3R and known orthologs from the clawed frog *Xenopus tropicalis*, spotted gar *Lepisosteus oculatus* and zebrafish *Danio rerio*. In the resulting tree, the medaka receptor ENSORLP00000035998 (red arrow) identified in this study is nested together with the human CX3C chemokine receptor (blue arrow). This orthology assignment is confirmed by conserved synteny with *ccr8* and *entpd3* in medaka and human, and also in spotted gar and zebrafish. We therefore refer to the encoding gene as medaka *cx3cr‐like*. Interestingly, this gene underwent independent lineage specific local gene duplications in zebrafish and medaka, while the other chemokine receptors have no such paralogs.

**Supplementary Figure S7** Phylogeny analysis of interleukin receptors. A molecular phylogeny was computed for proteins annotated as Il22ra2 and Il20ra in ENSEMBL (ENSORLP00000023762, ENSORLP00000033651; red arrows), respectively. This showed that both proteins cluster together with human IL22RA2 (blue arrow) and thus likely represent the product of a fish‐specific gene duplication. We therefore refer to the encoding genes as *il22ra2a* and *il22ra2b*. Conserved synteny with *lfngr1* was shown for *il22ra2a* that is present in human, zebrafish, spotted gar and medaka genomes. No synteny relation was detected for *il22ra2b* outside Perciformes, thus this gene represents a specific local gene duplication in this lineage.

**Supplementary Figure S8** *tnfa* expression and mutant establishment in medaka. (*A*) *tnfa* expression was analyzed by qPCR at 1, 2 and 3 dphs. Non‐heat shocked embryos were used as control. In embryos without RANKL transgene (‐RANKL), *tnfa* was downregulated after heat shock. In contrast, *tnfa* was upregulated in RANKL‐induced embryos (+RANKL) at 1 dphs, and significantly reduced at 2 and 3 dphs. Error bars indicate mean fold change ± SD, *0.01 < *p* < 0.05, **p < 0.01, ***p < 0.001, Student's *t* test, data from three sets of biological samples. (*B*) Design of guide RNAs for CRISPR/Cas9 to generate *tnfa* mutants. Three primers were used for genotyping. FP2 was used to differentiate between homozygous and heterozygous mutants. (*C*) Representative gel image showing PCR‐based genotyping. (*D*) 1010 bp deletion in *tnfa* leads to a predicted truncated protein.

**Supplementary Figure S9** Bone protection in PTX‐treated embryos during RANKL induction. Embryos were stained with Alizarin Red at 3 dphs. Both DMSO control and PTX‐treated larvae showed normal bone development in the absence of ectopic RANKL (blue arrowheads). Upon RANKL induction, control embryos exhibited severe bone loss in arches and vertebral bodies (black arrowheads). PTX‐treated embryos, on the other hand, showed only few mild lesions in arches (small black arrowheads) in otherwise normally mineralized centra (blue arrowheads). Scale bar: 500 μm.

**Movie S1** Macrophage behavior without RANKL overexpression. The 9 dpf *mpeg1:mCherry‐F/ctsk:GFP* transgenic embryo was incubated at 39°C for 2 hours and subjected to confocal imaging at 2 hphs. Tracks showed mpeg1*‐*positive macrophages actively patrolled around the body. Most macrophages were found in the AGM while only a few cells located proximal to the arches and vertebral bodies. AGM: aorta gonad mesonephros; VC: vertebral column; time: hh:mm:ss. Scale bar: 50 μm.

**Movie S2** Macrophage recruitment upon RANKL induction. Transgenic embryo *rankl:HSE:cfp/mpeg1:mCherry‐F/ctsk:GFP* was induced for RANKL overexpression by heat‐shock at 9 dpf and subjected to confocal imaging at 2 hphs. Mpeg1*‐*positive macrophages were massively recruited towards the vertebral column. The cells dynamically localized themselves particularly at the ossified matrix, neural arches, and vertebral bodies, but not at the intervertebral disc region. Boxes outline the vertebral bodies. AGM: aorta gonad mesonephros; V: vertebral body; na: neural arch; time: hh:mm:ss.

**Movie S3** Macrophage differentiation and clearance of apoptotic osteoclasts. RANKL‐induced *rankl:HSE:cfp/mpeg1:mCherry‐F/ctsk:GFP* embryo was subjected for confocal time‐lapse at 30 hphs. Arrowheads showed recruited macrophages along the trunk gradually differentiated into ctsk‐positive cells. Box highlighted the phagocytosis of apoptotic osteoclasts by macrophages. AGM: aorta gonad mesonephros; na: neural arch; time: hh:mm. Scale bar: 50 μm.

**Movie S4** Fusion of differentiating osteoclasts. A RANKL‐induced *rankl:HSE:cfp/mpeg1:mCherry‐F/ctsk:GFP* embryo was subjected to confocal time‐lapse at 54 hphs. During terminal differentiation, osteoclasts fused together into larger cells, both at the arches and the centra (blue boxes). White box showed an apoptosis event. AGM: aorta gonad mesonephros; time: hh:mm:ss. Scale bar: 50 μm.

**Movie S5** Normal osteoclast formation in Lipo‐PBS injected embryo. A transgenic *rankl:HSE:cfp/mpeg1:mCherry‐F/ctsk:GFP* embryo was injected with Lipo‐PBS, induced for RANKL and then subjected to confocal imaging at 1 dphs. Osteoclasts formed normally from the recruited macrophages (white arrowheads). AGM: aorta gonad mesonephros; VC: vertebral column; time: hh:mm. Scale bar: 50 μm.

**Movie S6** Absence of osteoclast formation in macrophage‐depleted embryo. A macrophage‐ablated transgenic *rankl:HSE:cfp/mpeg1:mCherry‐F/ctsk:GFP* embryo was induced for RANKL and subjected to confocal imaging at 1 dphs. Osteoclast formation was impaired after macrophages were ablated. AGM: aorta gonad mesonephros; VC: vertebral column; time: hh:mm. Scale bar: 50 μm.

**Movie S7** Multiplication of recruited macrophages and dynamic macrophage interaction with bone cells. A transgenic *rankl:HSE:cfp/col10a1:GFP/mpeg1:mCherry‐F* embryo at 9 dpf was RANKL‐induced at 39°C for 2 hours. Time‐lapse movie was started at 6 hphs. Arrows show macrophage multiplication upon recruitment to the vertebral column. White box highlights the dynamic interaction of macrophages with osteoblast precursors (*col10a1* cells). Time: hh:mm:ss. Scale bar: 50 μm.

**Supplementary Table S1**.
