# Supplementary figures and images for: Macrophages Switch to an Osteo‐Modulatory Profile Upon RANKL Induction in a Medaka (Oryzias latipes) Osteoporosis Model
Source: JBMR Plus. 2020 Oct 1;4(11):e10409. doi: 10.1002/jbm4.10409 (PMC7657398; doi:10.1002/jbm4.10409)

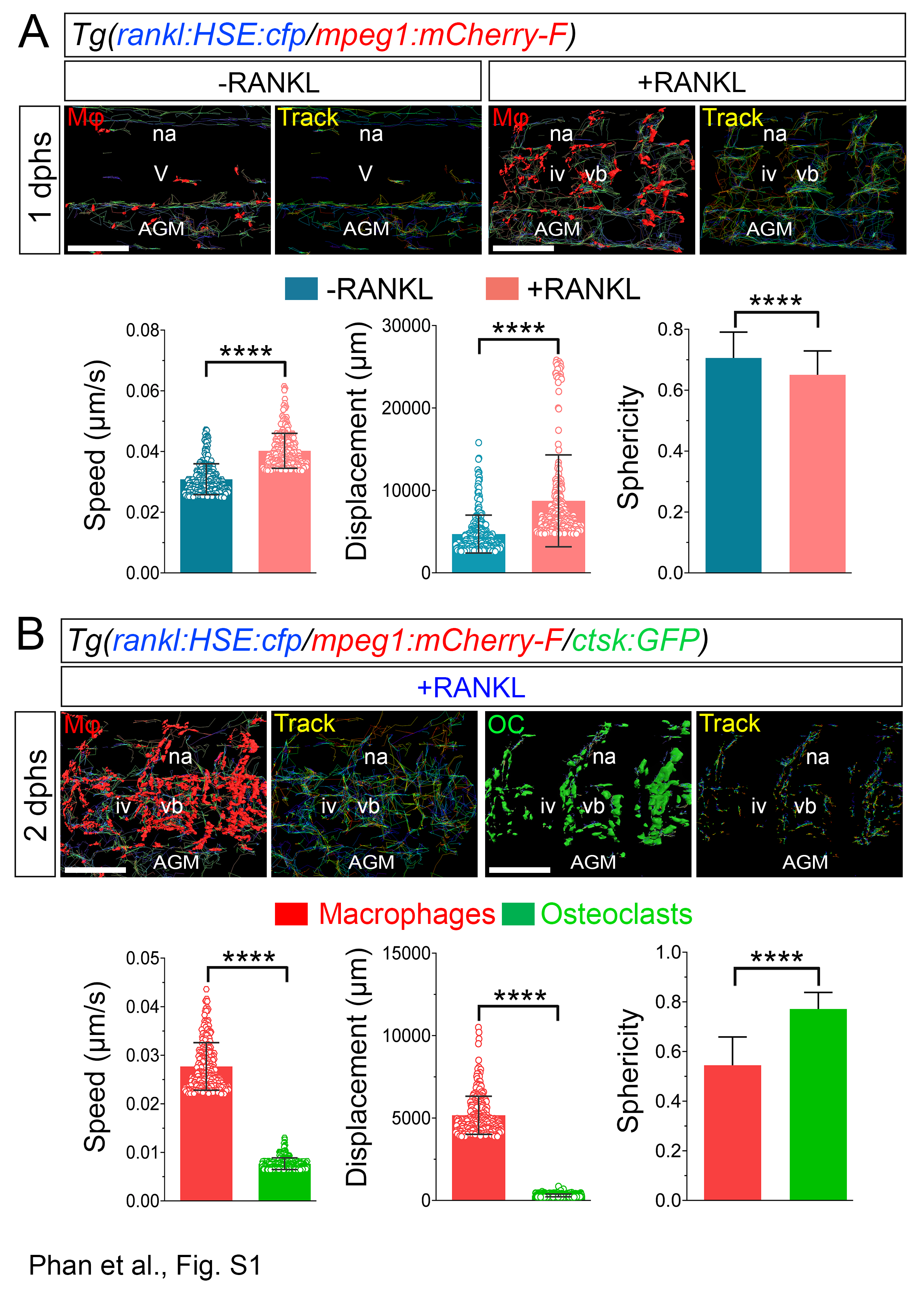

Supplement: Supplementary file 1 — Supplementary Figure S1 Macrophages become less motile after differentiation into osteoclasts. (A) Cell tracking showed that most macrophages remained in the AGM of ‐RANKL embryos. On the contrary, many macrophages were directionally recruited towards bone matrix of the vertebral column upon RANKL induction. The recruited cells clustered mainly at neural arches and vertebral bodies but not in the intervertebral discs. Macrophage dynamics was determined at 4 hphs. (B). At 1–2 dphs, macrophages that differentiated into osteoclasts (mpeg+/ctsk+) have significantly reduced motility and are confined to bone matrix. Macrophages that were recruited later stayed undifferentiated and remained highly motile. As the cells were more dynamic, they had angular shapes with lower sphericity. In contrast, mature osteoclasts became more static with rounded shapes (high sphericity) after fusion. Macrophages dynamics was determined at 34 hphs. Student's t test was used to determine the significance of difference between groups, error bars show standard deviation. AGM: aorta gonad mesonephros; V: vertebral column; iv: intervertebral disc; vb: vertebral body; na: neural arch; Mφ: macrophage; OC: osteoclast. Scale bar: 100 μm. [file JBM4-4-e10409-s001.tif]

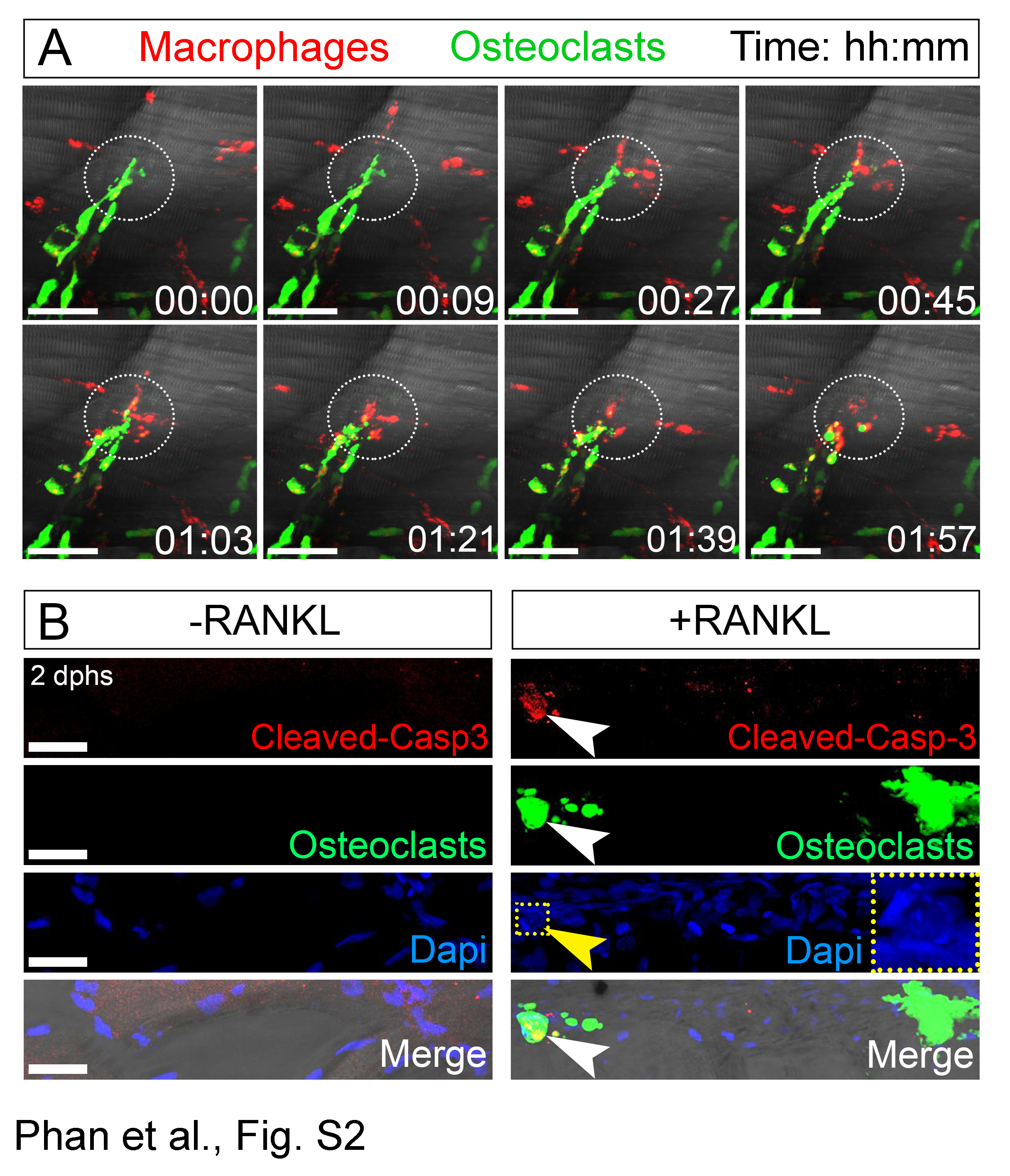

Supplement: Supplementary file 2 — Supplementary Figure S2 Macrophages clear apoptotic osteoclasts. (A) Still images from a time‐lapse movie recorded at 30 hphs. Circles show that apoptotic osteoclasts (green cells) at the neural arch are engulfed and digested by conventional phagocytosing macrophages. Time:hh:mm. (B) Immunostaining of cleaved‐Caspase‐3 on cryosections revealed apoptosis (white arrowheads) of osteoclasts in RANKL‐induced embryo. Scale bar: 20 μm (in A), 40 μm (in B). [file JBM4-4-e10409-s002.tif]

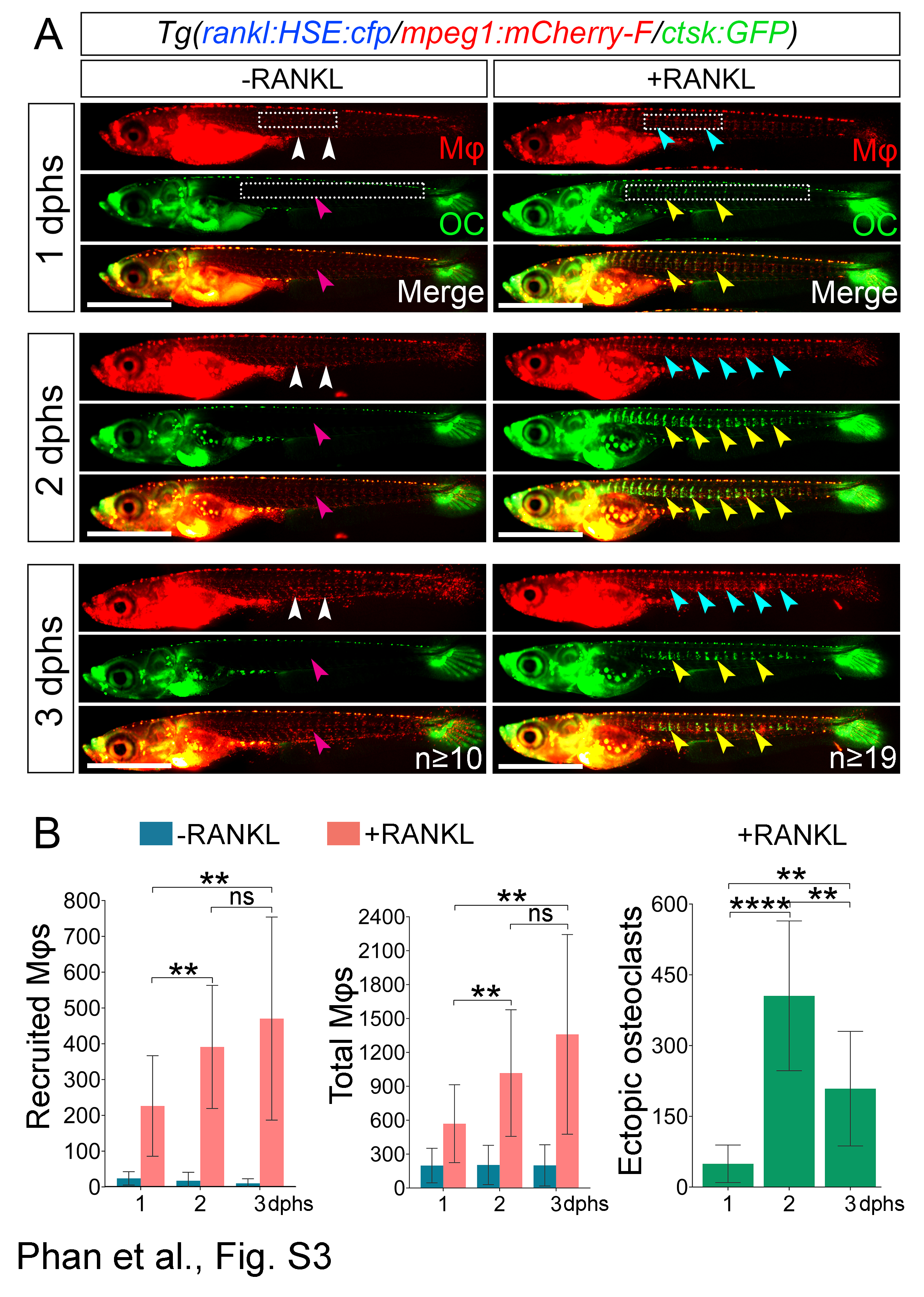

Supplement: Supplementary file 3 — Supplementary Figure S3 Quantification of macrophage and osteoclast numbers. (A) rankl:HSE:cfp/mpeg1:mCherry‐F/ctsk:GFP and mpeg1:mCherry‐F/ctsk:GFP transgenic embryos were heat‐shocked and imaged repeatedly at 1, 2 and 3 dphs. In the absence of ectopic RANKL expression, macrophages were distributed at different positions along the body, predominantly in the AGM (white arrowheads). There were no ectopic osteoclasts in the trunk (magenta arrowheads). Upon RANKL induction, macrophages were directionally recruited towards the vertebral column, particularly to the neural arches and the vertebral bodies (cyan arrowheads). Osteoclasts were later formed by differentiation of recruited macrophages along the trunk (yellow arrowheads). The ectopic osteoclasts started to vanish at 3 dphs while new macrophages continued to be recruited. (B) Quantification showed mean numbers of recruited macrophages, total macrophages and ectopic osteoclasts ± SD; Student's t test (two‐tailed, unpaired), **p˂0.01, ****p˂0.0001, ns: non‐significant, 10 ≤ NLarvae ≤ 19, data from three independent experiments. Mφ: macrophage; OC: osteoclast. Scale bar: 1 mm. [file JBM4-4-e10409-s003.tif]

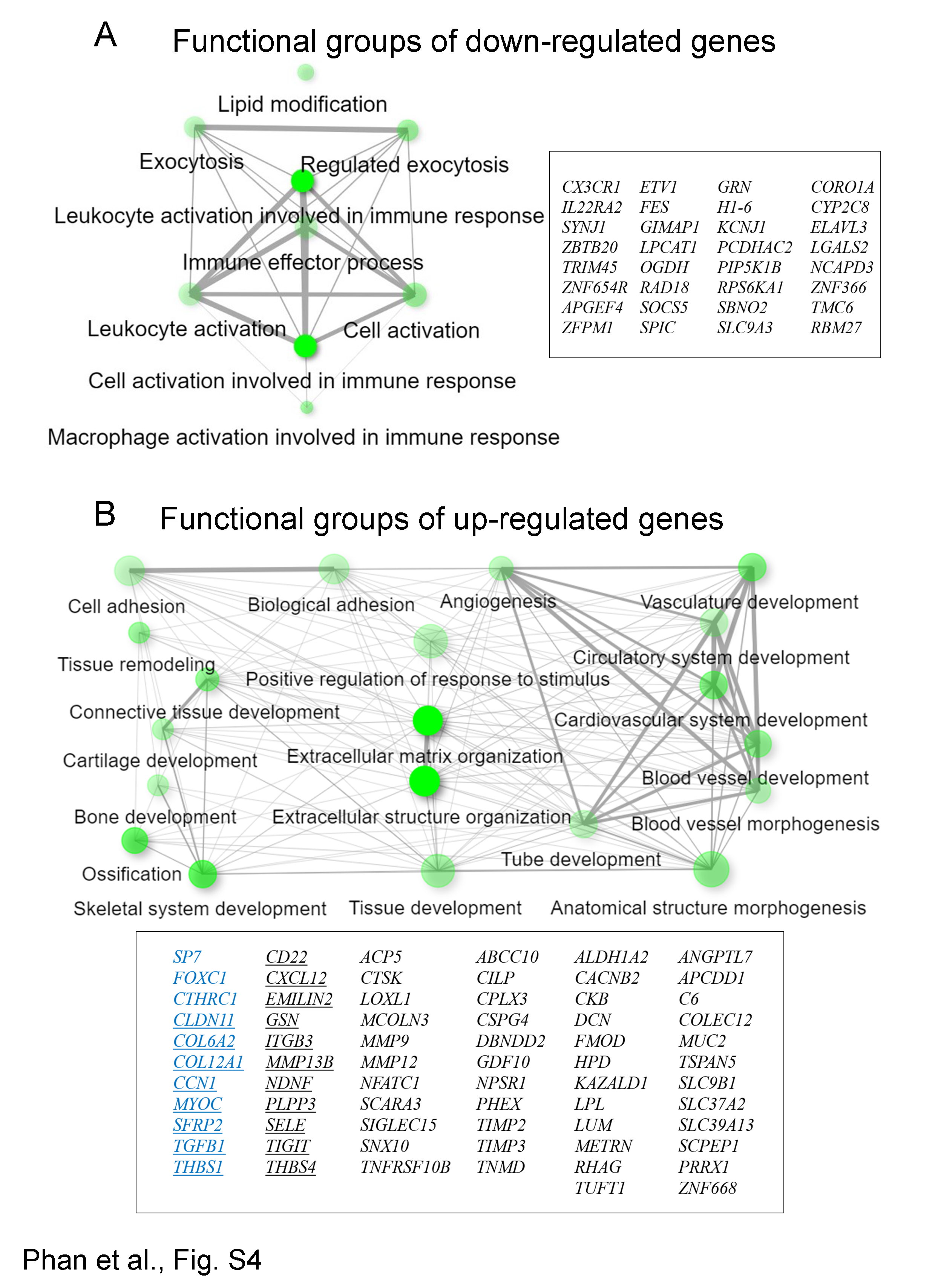

Supplement: Supplementary file 4 — Supplementary Figure S4 Gene Ontology analysis of up‐ and downregulated genes in osteoclast compared with macrophage. (A,B) Functional groups in down‐ and upregulated genes, respectively. Listed genes were used as input for GO terms analysis using ShinyGO. Upregulated genes involved in osteoblast differentiation and bone remodeling are labeled in blue, and genes related to adhesion were underlined (B). [file JBM4-4-e10409-s004.tif]

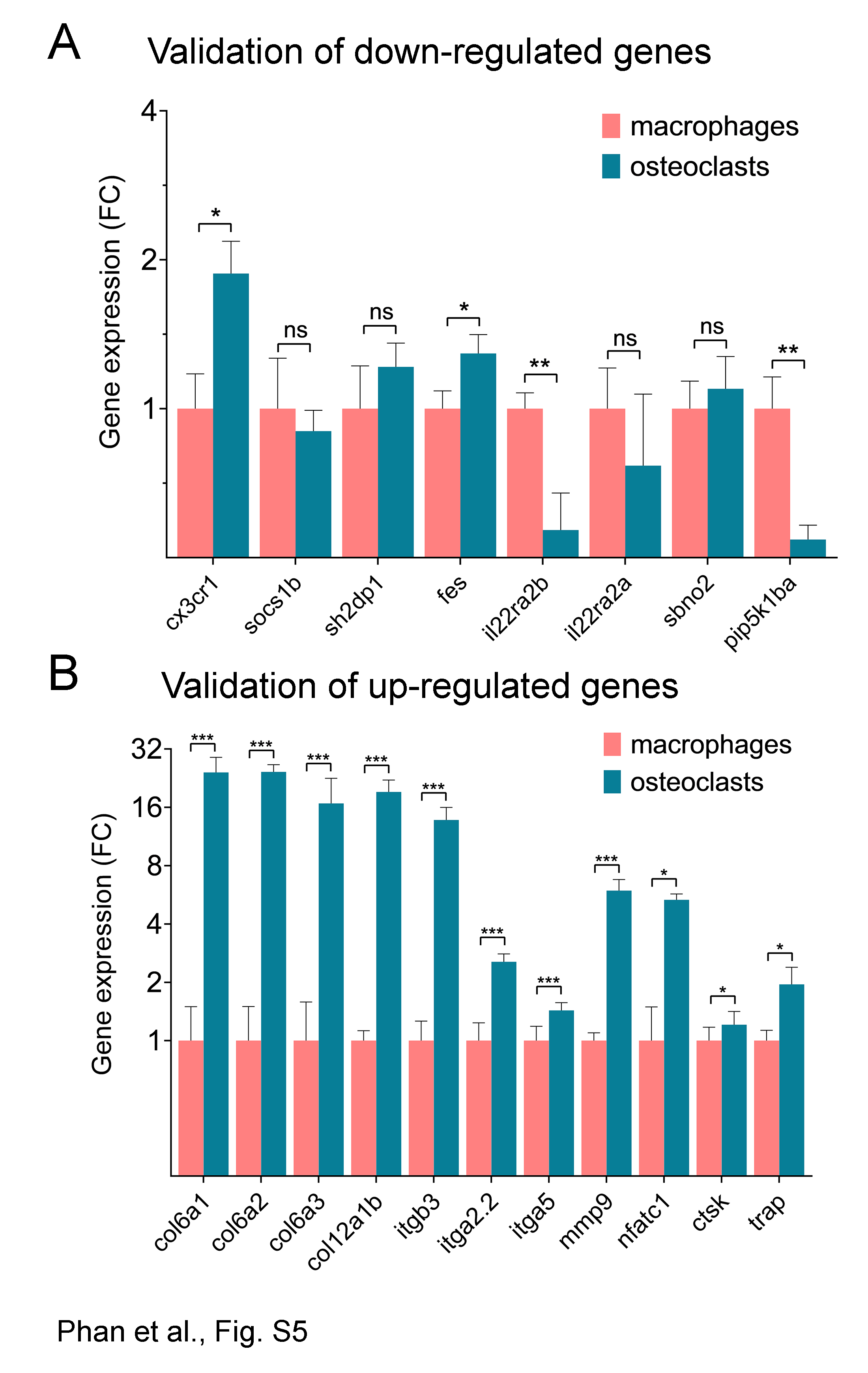

Supplement: Supplementary file 5 — Supplementary Figure S5 qPCR validation of gene expression in FAC‐sorted macrophages and osteoclasts. Gene expression was determined at 1 dphs. (A) Among downregulated genes, il22ra2a, il22ra2b and pip5k1ba showed trends of downregulation in osteoclasts compared with macrophages, consistent with RNAseq data. In contrast, cx3cr1 and fes show upregulation, while socs1b, sh2dp1 and sbno2 were not regulated. (B) Among upregulated genes, genes encoding collagen VI, collagen XII and integrin subunit beta 3 and their binding subunits were significantly higher expressed in osteoclasts, consistent with RNAseq data. Similarly, expression of osteoclast makers nfatc1, trap and ctsk were also upregulated. Error bars indicate mean fold change ± SD, *p < 0.05, **p < 0.01, ***p < 0.001, ns: non‐significant, Student's t test, data were obtained from three biological replicates with three technical repeats each and β‐actin was used as loading control. [file JBM4-4-e10409-s005.tif]

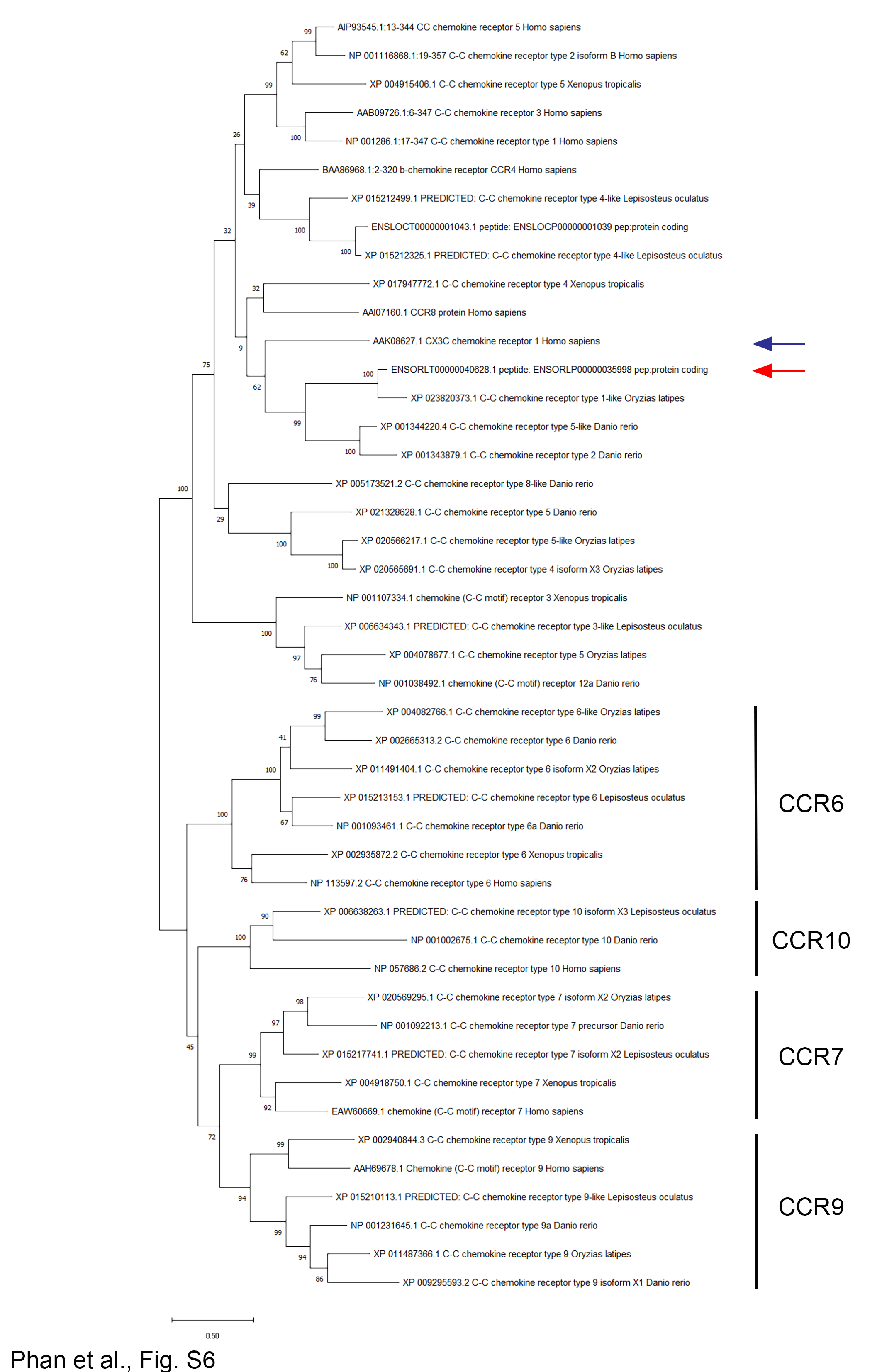

Supplement: Supplementary file 6 — Supplementary Figure S6 Phylogeny analysis of CC chemokine receptors. A molecular phylogeny was computed for all so far identified medaka Ccr receptors as well as human CCRs 1–9, human CX3R and known orthologs from the clawed frog Xenopus tropicalis, spotted gar Lepisosteus oculatus and zebrafish Danio rerio. In the resulting tree, the medaka receptor ENSORLP00000035998 (red arrow) identified in this study is nested together with the human CX3C chemokine receptor (blue arrow). This orthology assignment is confirmed by conserved synteny with ccr8 and entpd3 in medaka and human, and also in spotted gar and zebrafish. We therefore refer to the encoding gene as medaka cx3cr‐like. Interestingly, this gene underwent independent lineage specific local gene duplications in zebrafish and medaka, while the other chemokine receptors have no such paralogs. [file JBM4-4-e10409-s006.tif]

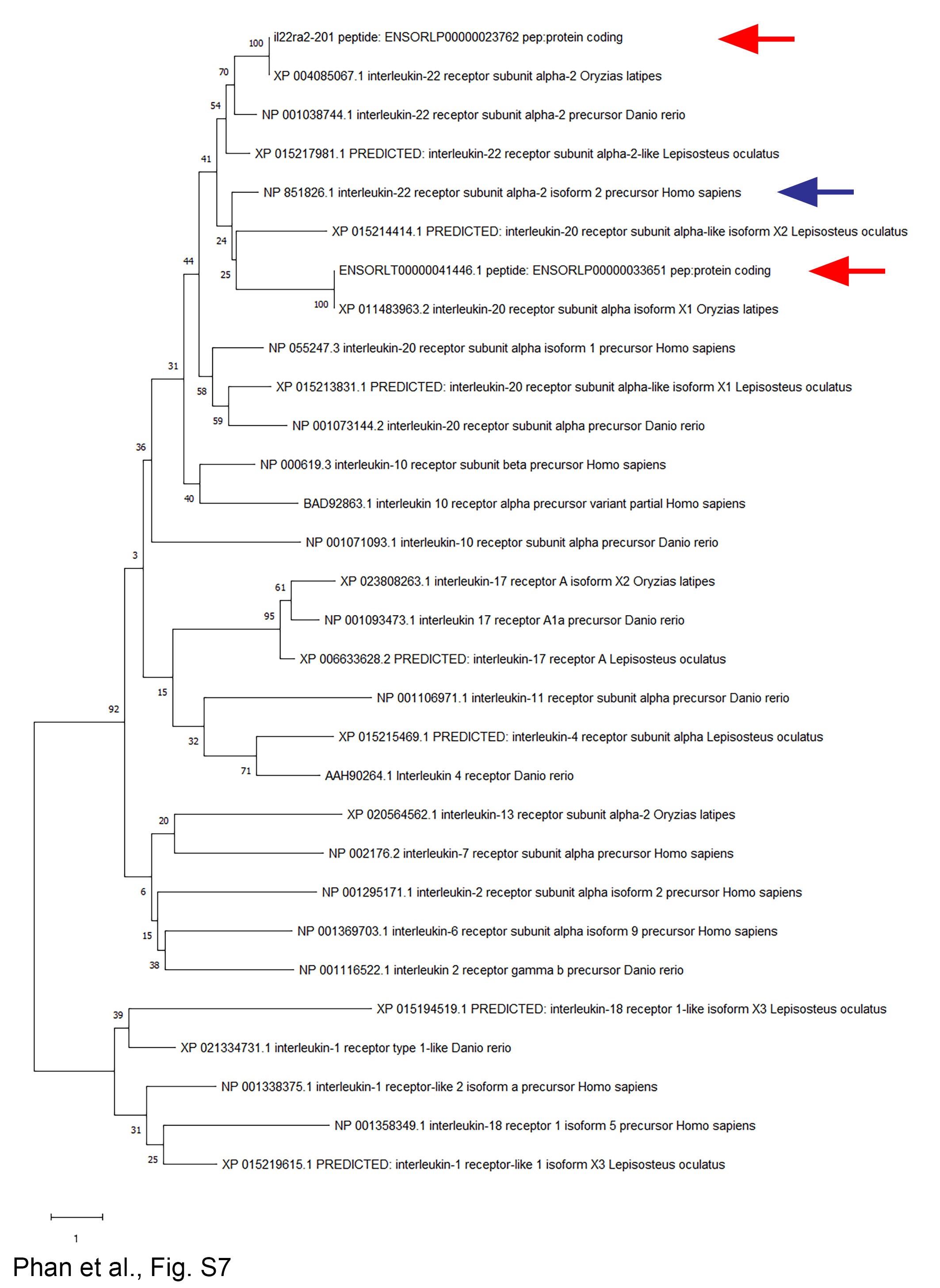

Supplement: Supplementary file 7 — Supplementary Figure S7 Phylogeny analysis of interleukin receptors. A molecular phylogeny was computed for proteins annotated as Il22ra2 and Il20ra in ENSEMBL (ENSORLP00000023762, ENSORLP00000033651; red arrows), respectively. This showed that both proteins cluster together with human IL22RA2 (blue arrow) and thus likely represent the product of a fish‐specific gene duplication. We therefore refer to the encoding genes as il22ra2a and il22ra2b. Conserved synteny with lfngr1 was shown for il22ra2a that is present in human, zebrafish, spotted gar and medaka genomes. No synteny relation was detected for il22ra2b outside Perciformes, thus this gene represents a specific local gene duplication in this lineage. [file JBM4-4-e10409-s007.tif]

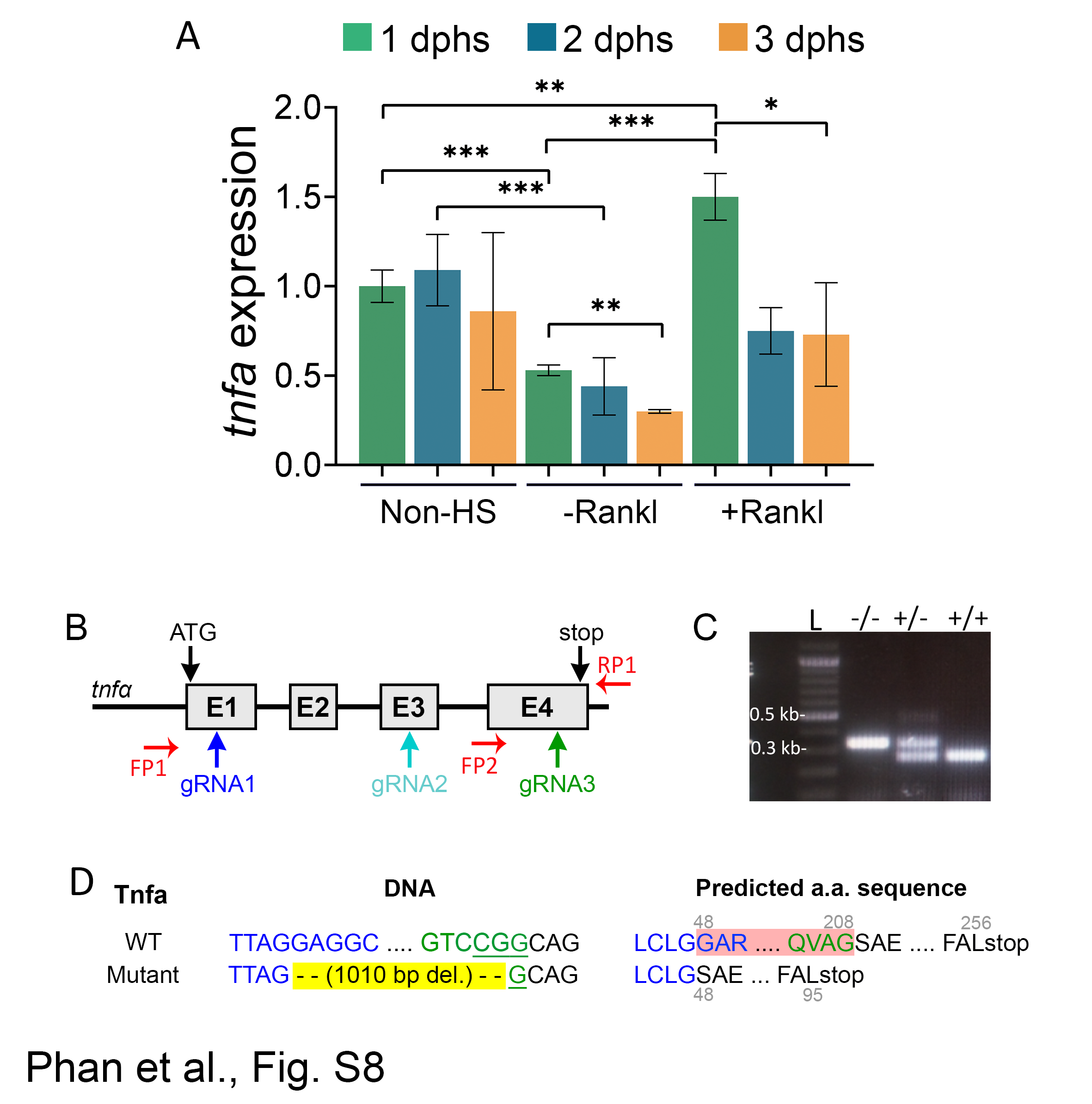

Supplement: Supplementary file 8 — Supplementary Figure S8 tnfa expression and mutant establishment in medaka. (A) tnfa expression was analyzed by qPCR at 1, 2 and 3 dphs. Non‐heat shocked embryos were used as control. In embryos without RANKL transgene (‐RANKL), tnfa was downregulated after heat shock. In contrast, tnfa was upregulated in RANKL‐induced embryos (+RANKL) at 1 dphs, and significantly reduced at 2 and 3 dphs. Error bars indicate mean fold change ± SD, *0.01 < p < 0.05, **p < 0.01, ***p < 0.001, Student's t test, data from three sets of biological samples. (B) Design of guide RNAs for CRISPR/Cas9 to generate tnfa mutants. Three primers were used for genotyping. FP2 was used to differentiate between homozygous and heterozygous mutants. (C) Representative gel image showing PCR‐based genotyping. (D) 1010 bp deletion in tnfa leads to a predicted truncated protein. [file JBM4-4-e10409-s008.tif]

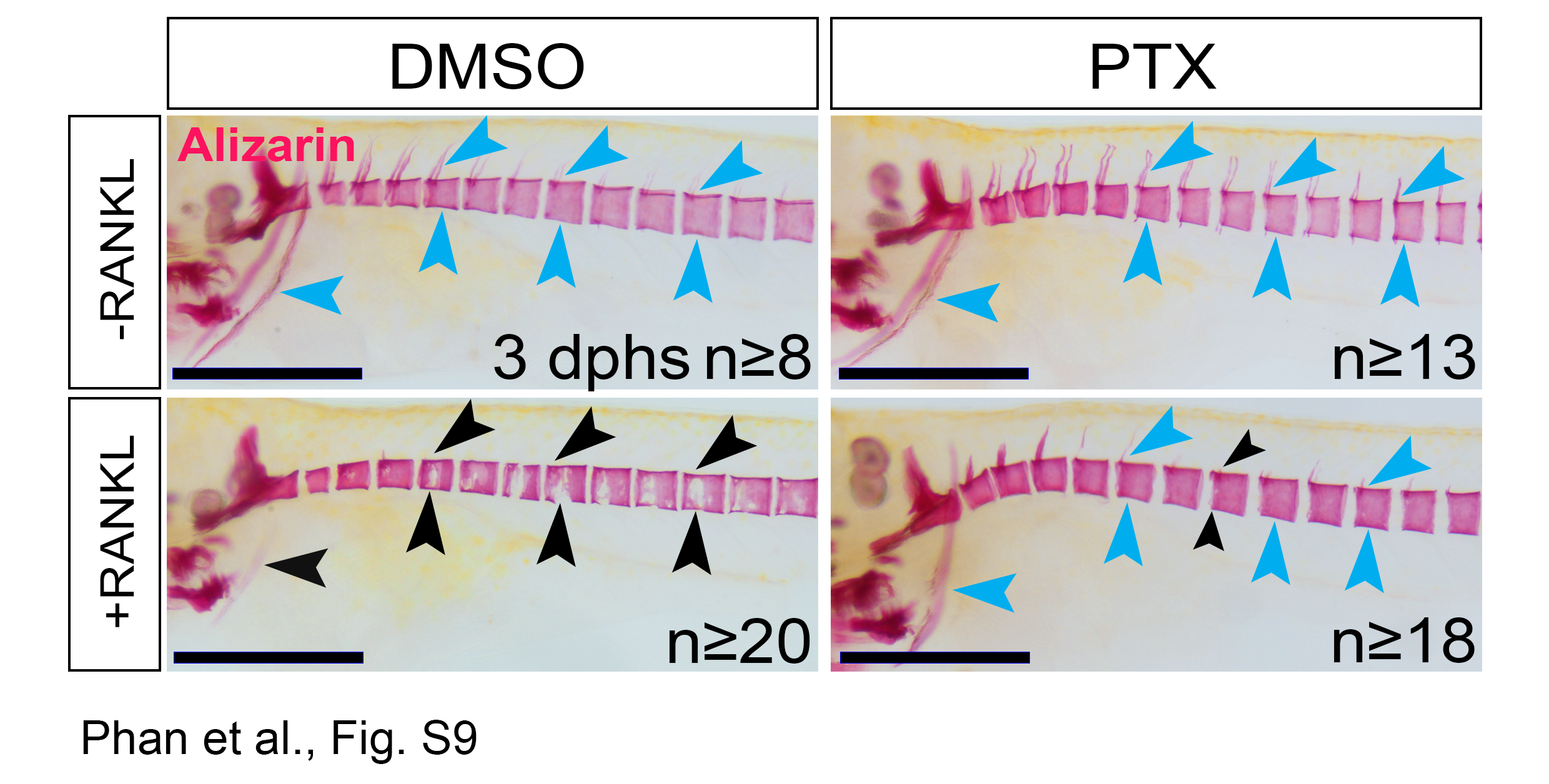

Supplement: Supplementary file 9 — Supplementary Figure S9 Bone protection in PTX‐treated embryos during RANKL induction. Embryos were stained with Alizarin Red at 3 dphs. Both DMSO control and PTX‐treated larvae showed normal bone development in the absence of ectopic RANKL (blue arrowheads). Upon RANKL induction, control embryos exhibited severe bone loss in arches and vertebral bodies (black arrowheads). PTX‐treated embryos, on the other hand, showed only few mild lesions in arches (small black arrowheads) in otherwise normally mineralized centra (blue arrowheads). Scale bar: 500 μm. [file JBM4-4-e10409-s009.tif]
