# Supplementary material for: Macrophages Switch to an Osteo‐Modulatory Profile Upon RANKL Induction in a Medaka (Oryzias latipes) Osteoporosis Model
Source: JBMR Plus. 2020 Oct 1;4(11):e10409. doi: 10.1002/jbm4.10409 (PMC7657398; doi:10.1002/jbm4.10409)
Supplement: Supplementary file 17 — Supplementary Table S1. [file JBM4-4-e10409-s017.docx]

**Macrophages switch to an osteo-modulatory profile upon Rankl induction in a medaka (*Oryzias latipes*) osteoporosis model**

Quang Tien Phan^1^, Ranran Liu^1^, Wen Hui Tan^1^, Nurgul Imangali^1^, Benedict Cheong^1^, Manfred Schartl^2,3^ and Christoph Winkler^1,#^

^1^Department of Biological Sciences and Centre for Bioimaging Sciences, National University of Singapore, Singapore 117543, Singapore

^2^Department of Developmental Biochemistry, Biocenter, University of Würzburg, Würzburg 97080, Germany

^3^The *Xiphophorus* Genetic Stock Center, Texas State University, San Marcos, Texas 78666-4684, USA.

**Running title:** Osteoclast differentiation in medaka

The authors declare that they do not have any conflict of interest. All data presented in this manuscript will be shared upon request.

^#^Corresponding author:

Christoph Winkler

Department of Biological Sciences, National University of Singapore

14 Science Drive 4, S1A-06-07, Singapore 117543

Phone: +65-6516 7376

dbswcw@nus.edu.sg

**Abstract**

In mammals, osteoclasts differentiate from macrophages in the monocyte lineage. Although many factors driving osteoclast formation are known, the detailed processes underlying precursor recruitment, differentiation and interaction of macrophages with other cell types involved in bone remodeling are poorly understood. Using live imaging in a transgenic medaka osteoporosis model where ectopic osteoclasts are induced by Receptor activator of nuclear factor-kappa Β ligand (Rankl) expression, we show that a subset of macrophages is recruited to bone matrix to physically interact with bone forming osteoblast progenitors. These macrophages subsequently differentiate into *cathepsin K* (*ctsk*)-positive osteoclasts. One day later, other macrophages are recruited to clear dying osteoclasts from resorbed bone by phagocytosis. To better understand the molecular changes underlying these dynamic processes, we performed transcriptome profiling of activated macrophages upon Rankl induction. This revealed an upregulation of several bone-related transcripts. Besides osteoclast markers, we unexpectedly also found expression of osteoblast-promoting signals in activated macrophages, suggesting a possible non-cell autonomous role in osteogenesis. Finally, we show that macrophage differentiation into osteoclasts is dependent on inflammatory signals. Medaka deficient for Tumor necrosis factor alpha (Tnfa) or treated with the Tnfa inhibitor pentoxifylline exhibited impaired macrophage recruitment and osteoclast differentiation. These results demonstrate the involvement of inflammatory signals and the dynamics of a distinct subset of macrophages during osteoclast formation.

**Keywords**: Macrophages; osteoclasts; Rankl; Tnfa; bone resorption; bone homeostasis

**INTRODUCTION**

Since their initial description in 1883 ^(1)^, macrophages have been intensively studied for their involvement in various physiological processes including embryogenesis, infection, tissue regeneration and bone homeostasis (reviewed in ^(2)^). Bone marrow-derived monocytes and macrophages have been identified as precursors of *cathepsin K* (*ctsk*)-positive osteoclasts, which digest mineralized matrix and thereby function in bone resorption ^(3,4)^. On the other hand, osteal macrophages, a subpopulation of specialized bone-homing macrophages, were shown to have essential roles in bone formation during regeneration and fracture healing ^(5,6)^. This cell type also modulates cytokine expression and performs clearance of apoptotic cells known as efferocytosis ^(7)^. During an inflammation episode, the activation of macrophages often includes dynamic regulation of pro-inflammatory cytokines, especially Interleukin-1b (Il1b) and Tumor necrosis factor-alpha (Tnfa). These cytokines trigger an immune response and recruit other immune cells to inflammation sites ^(7,8)^. Tnfa, a member of the tumor necrosis factor superfamily, is produced by different cell types such as monocytes, neutrophils and adipocytes ^(9,10)^. It plays important roles in cell proliferation, wound healing and cancer progression ^(8,11)^. Tnfa is also found predominantly expressed in activated macrophages in inflammatory diseases such as Rheumatoid Arthritis (RA) ^(12)^. Inhibition of this cytokine has been an effective therapy to alleviate bone inflammatory symptoms ^(13)^. Osteoporosis patients, who also have elevated serum levels of IL1β and TNFa, often experience severe bone loss and fractures that result from hyperactivity of osteoclasts ^(14)^. Several studies have shown that in the presence of macrophage colony stimulating factor (M-CSF), TNFa and RANKL synergistically or independently stimulate osteoclast formation via their respective receptors ^(15-17)^. However, how these cytokines control the dynamics of osteoclast precursor recruitment and differentiation *in vivo* remains unclear, mostly because commonly used animal models have limited accessibility for live imaging.

In this study, we used a transgenic medaka osteoporosis model, where excessive osteoclast formation is triggered by inducible Rankl expression ^(18)^. Upon Rankl induction, macrophages migrate to the vertebral column where they eventually mature into *ctsk*-positive osteoclasts ^(19)^. In the present study, we describe the dynamics of macrophage migration and their close interaction with bone-lining osteoblasts. We report that *tnfa* is upregulated in activated macrophages during recruitment and that deletion of *tnfa* or treatment with the *tnfa* transcription inhibitor pentoxifylline blocks macrophage recruitment and osteoclast differentiation. Transcriptome profiling further revealed that the cytokine receptor gene *il22ra2b* was downregulated when macrophages differentiate into osteoclasts. Together, these findings suggest that inflammatory signals are initially required for macrophage recruitment towards bone matrix under osteoporotic conditions. Subsequently, these signals are downregulated to allow differentiation of recruited cells into osteoclasts. Interestingly, we also find that upon Rankl induction, macrophages upregulate genes that are known to control chondrocyte and osteoblast differentiation in a non-cell autonomous manner. This suggests an involvement of activated macrophages in bone remodelling and homeostasis under pathological conditions.

**MATERIALS AND METHODS**

**Ethics statement**

All experiments were performed according to protocols approved by the Institutional Animal Care and Use Committee (IACUC) of the National University of Singapore (NUS; protocol numbers R14-293, R18-0562 and BR15-0119).

**Transgenic and mutant medaka lines**

Transgenic *rankl:HSE:cfp/ctsk:GFP* medaka embryos were grown and subjected to Rankl induction by heat-shock as previously described ^(18)^. For generating the macrophage reporter line, a 2.1-kb sequence of the mpeg1 promoter including the endogenous ATG was amplified from genomic DNA of wild-type medaka using primers mpeg1.2F and mpeg1.2R (**Supplementary Table 1**). The PCR product was digested with *NotI* and *BamHI* and then ligated in-frame to a farnesylated mCherry in a pI-*SceI* plasmid. The plasmid was microinjected into one-cell stage medaka embryos together with meganuclease *I-SceI* enzyme as described before ^(20)^. Injected embryos were screened for mCherry-F transgene expression in macrophages, and stable lines were established and verified by in situ hybridization. For the generation of *tnfa* mutants, guide RNAs (gRNAs) targeting exons 1, 3 and 4 of *tnfa* were designed using CCTop ^(21)^ and synthesized by IDT (Singapore) (**Fig. S8; Supplementary Table 1**). The three gRNAs were mixed with tracrRNA and HiFi Cas9 protein (IDT, Singapore) and injected into one-cell stage medaka embryos. Injected fish and their offspring were genotyped using primers listed in **Supplementary Table 1**, and stable mutant lines were established.

**Macrophage depletion and drug treatment**

For macrophage depletion, *rankl:HSE:cfp/mpeg1:mCherry-F/ctsk:GFP* embryos at nine days post fertilization (dpf) were injected intravenously with 15 nl Lipo-Clodronate (5 mg/ml) (Lipo-Clo) or Lipo-PBS (Encapsula, USA). Macrophage-ablated embryos were selected at 10 dpf based on the reduced fluorescence signal of mCherry-F reporter. For inhibition of *tnfa* expression, Rankl-induced embryos after heat-shock were immediately transferred to fish medium (30% Danieau’s solution containing 19.3 mM NaCl, 0.23 mM KCl, 0.13 mM MgSO4, 0.2 mM Ca(NO3)2, 1.7 mM HEPES, pH 7.0) supplemented with 200 μM pentoxifylline (Sigma-Aldrich; Germany) or 0.05% DMSO as control. The medium was changed daily with fresh drug added for the whole course of the experiments.

**In situ hybridization**

Sense and anti-sense riboprobes spanning 790 nucleotides of the *mpeg1* cDNA (nt1029-nt1818) (ENSORLT00000006101.2; Ensembl.org) were synthesized using a DIG RNA Labeling Kit (Roche, Switzerland). In situ hybridization was performed as described before ^(18)^.

**Bone staining**

Alizarin Red staining of mineralized bone matrix in fixed embryos was performed as previously described ^(22)^. Briefly, larvae at 3 days post heat-shock (dphs) were fixed with 4% PFA, washed three times with PBST, incubated 15 mins in 0.5% KOH, then transferred to 0.001% Alizarin Red solution in 0.5% KOH for at least 4 hours with agitation. Samples were washed for 2 hours with 0.5% KOH followed by depigmentation with 2% H_2_O_2_ in 0.5 % KOH for 1 hour. Embryos were washed two times with 0.5 % KOH and mounted in 100 % glycerol for imaging. For live bone staining, embryos were incubated in 0.01% Calcein (Sigma-Aldrich; Germany) and kept in the dark for at least one hour at 30^o^C. Embryos were washed twice with fish medium and mounted in low-melting agarose for imaging.

**Cryosectioning and immunostaining**

*rankl:HSE:cfp/ctsk:GFP* transgenic embryos were fixed in 4 % PFA, washed with PBST and mounted in 1.5 % low-melting agarose in 5 % sucrose. The set agar was kept overnight at 4°C in 30 % sucrose before cryosectioning using a Cryostat 1850 (Leica, Germany). Sections of 20 μm were collected on SuperFrost^TM^ Plus slides (Thermo Fisher Scientific, USA) and dried at room temperature (RT). For immunostaining, sections were incubated in 10% goat serum in PBST (3.2 mM Na_2_HPO_4_ , 0.5 mM KH_2_PO_4_ , 1.3 mM KCl, 135 mM NaCl, 0.05% Tween 20) (blocking buffer, BF) for 1 hour at RT and incubated overnight at 4°C with a 1:100 dilution of Cleaved-Caspase-3 antibody (Asp175, Cell Signaling Technology, USA) in BF. Alexa Fluor 633 IgG goat anti-rabbit (Invitrogen, USA) was used as secondary antibody at a 1:500 dilution. Samples were washed with PBS, stained with DAPI and kept in Mowiol (Sigma-Aldrich, Germany) until imaging.

**Imaging of live and fixed samples**

For live imaging, embryos were anesthetized in 0.016% Tricaine (MS-222, Sigma-Aldrich, Germany) and embedded in 1.2% low melting agarose (Bio-Rad, USA) in 35 mm glass-bottom dishes (Eppendorf, Germany), and covered with 2 ml fish medium containing Tricaine. A Nikon SMZ18 epi-fluorescence microscope was used for capturing whole-embryo images. Z-stack images and time-lapse movies were recorded using a Nikon FV3000 confocal microscope equipped with a 30X/1.05, WD 0.8 silicon oil objective. Time-lapse files were processed using Imaris (Bitplane) and Fiji (NIH) to generate movies. Z-stack images were processed into maximum intensity projections and channels merged using Fiji. Alizarin Red stained bone samples were imaged using a Nikon Eclipse 90i upright microscope equipped with NIS-Elements version BR 3.0.

**Cell quantification and statistics**

Fluorescent images of whole embryos and confocal z-stacks were used for the quantification of macrophage and osteoclast numbers as previously described ^(23)^ with slight modifications. Using Fiji, fluorescent images were converted to greyscale, thresholded, and the area of a cell (averaged from fifteen cells from three embryos), as well as the total area of cells in a selected region of interest, were derived. Cell numbers were determined by dividing the total area of cells with the average cell area. For statistical analysis, Student’s t-test (two-tailed, unpaired) was performed using GraphPad Prism 8.0 (GraphPad Software).

**Fluorescence-activated cell sorting, RNA sequencing and bioinformatics analysis**

Fluorescence-activated cell sorting (FACS) and RNA extraction were performed using previously described protocols ^(24)^ with some modifications. RNA library construction, Illumina sequencing, and bioinformatics analysis were performed by Novogene following company procedures (Novogene, Singapore). Briefly, Rankl expression was induced in *rankl:HSE:cfp/mpeg1:mCherry-F/ctsk:GFP* transgenic medaka embryos at 9 dpf. At 1 dphs, 23 larvae/sample were dissociated in 500 µl of 0.25% Trypsin EDTA (Hyclone^TM^, GE) plus collagenase (0.2 mg/ml) at 30^0^C and pipetted every 10 mins for 40 mins. The digestion was stopped by adding 50 µl sterile-filtered fetal bovine serum (FBS). Cells were washed with PBS supplemented with 2% FBS and resuspended in 400 µl Leibovitz’s L-15 medium (Gibco, Thermo Fisher Scientific, USA) for FACS using a BD FACSAria II platform (BD Biosciences, USA). Fluorescent macrophages and osteoclasts were separated and collected into 400 µl Trizol (Thermo Fisher Scientific, USA). RNA extraction was done from three independent biological replicates using PureLink RNA Micro Kit (Thermo Fisher Scientific, USA). The RNA quality was determined with an Agilent Bioanalyzer 2100 using Agilent RNA 6000 Pico Kit (Agilent Technologies, USA). Samples with RIN values >7 were submitted for sequencing. Clean reads were aligned to the reference genome for Japanese medaka HdrR, version ASM223467v1 (Ensmebl.org) using TopHat v2.0.12. Differential expression analysis of two biological replicates per condition was performed using Deseq R package (1.18.0). P-values were adjusted using Benjamini and Hochberg’s approach and genes with P-values less than 0.05 were assigned as differentially expressed. Gene Ontology (GO) enrichment of differentially expressed genes was analyzed using GOseq R package and only GO terms with a P-value less than 0.05 were considered significantly enriched. ShinyGO V0.61 was used to analyse networks of functional groups of up- and downregulated genes. Input data were matched to human genes, the P-value cut off was 0.05, and the 30 most significant terms were used (<http://bioinformatics.sdstate.edu/go/>).

**Phylogeny and synteny analysis**

Amino acid sequences were retrieved from the National Center for Biotechnology Information (NCBI; [www.ncbi.nlm.nih.gov/](http://www.ncbi.nlm.nih.gov/)) and ENSEMBL ([www.ensembl.org/Multi/Tools/Blast](http://www.ensembl.org/Multi/Tools/Blast)) databases using BLAST searches with default parameters. Sequences were aligned using Clustal W ^(25)^ and trees constructed using the Maximum Likelihood method in MEGA version X ^(26)^ with the JTT model for aa substitutions and 1000 bootstrap iterations. Conserved synteny analysis was done using GENOMICUS version 100.01 (<https://www.genomicus.biologie.ens.fr/genomicus-100.01/cgi-bin/search.pl>) ^(27)^.

**Quantitative PCR**

For whole embryos, RNA was isolated from ten embryos per sample using NucleoSpin RNA Kit (Macherey-Nagel, Germany), and total mRNA was reverse transcribed using the RevertAid First Strand cDNA Synthesis Kit (Thermo Fisher Scientific, USA). For RNA isolation from FAC-sorted macrophages and osteoclasts, the NucleoSpin RNA XS Kit (Macherey-Nagel, Germany) was used. RNA isolated from three independent biological samples was converted into cDNA and pre-amplified using Fluidigm Reverse Transcription Master Mix and Preamp Master Mix, respectively (Fluidigm, USA). All steps were performed following the manufacturers’ standard protocols. PowerUp SYBR Green Master Mix (Applied Biosystems, USA) was used for qPCR conducted in a CFX96 Touch system (Bio-Rad Laboratories, USA). Data analysis was performed using Bio-Rad’s CFX Maestro 1.0 software with β-actin as loading control for normalization. Two-tailed Student’s t-tests were performed for statistical analysis.

**RESULTS**

**Rankl triggers macrophage recruitment, proliferation, and differentiation at bone matrix**

To study the dynamic behavior of macrophages by live imaging in medaka, we generated a reporter line expressing farnesylated mCherry under control of the medaka *mpeg1* promoter (**Fig. 1A**). At 10 days post fertilization (dpf), *mpeg1:mCherry-F* positive macrophages were found throughout the body with increased numbers in heart, liver, aorta-gonad-mesonephros (AGM) and caudal fin (**Fig. 1B**). The *mpeg1* transgenic line faithfully recapitulated endogenous *mpeg1* transcription as confirmed by RNA in-situ hybridization (**Fig. 1C-G**). Next, *mpeg1:mCherry-F* reporter fish were crossed with *rankl:HSE:cfp/ctsk:GFP* transgenic fish, which harbor a Rankl transgene under control of a bidirectional heat-shock promoter that also drives CFP, and express GFP in osteoclasts under control of the *ctsk* promoter ^(^for details see ^18)^. Confocal time-lapse imaging showed that in the absence of ectopic Rankl expression (-Rankl), the majority of *mpeg1:mCherry-F* positive macrophages reside within the AGM with only few migratory cells found in other regions of the body including the vertebral column (**Supplementary** **Fig. S1A; Movie S1**). Shortly after Rankl induction (4 hours post heat-shock, hphs), *mpeg1*-positive macrophages started to accumulate in the vertebral column (**Fig. 2A, B**). Time-lapse analysis revealed that the majority of *mpeg1* cells in the vertebral column originated from the AGM but also cells from other regions were recruited (**Supplementary Fig. S1**). Macrophages were almost exclusively found at the mineralized matrix of vertebral bodies, but not in the non-mineralized intervertebral discs (**Fig. 2B; Supplementary Fig. S1A; Movie S2)**. In vertebral bodies, macrophages were in close contact with osteoblasts and osteoblast progenitors, which line the bone surface (**Movie S7**). At 30-64 hphs, the recruited macrophages gradually differentiated into *ctsk*-positive osteoclasts (**Fig. 2C-D; Movie S3-S4**).

Starting from 35 hphs, the first osteoclasts started to undergo cell death and became cleared by newly recruited phagocytic macrophages (**Supplementary Fig. S2; Movie S3-S4**). Quantification of *mpeg1:mCherry-F* and *ctsk:GFP* cells showed high numbers of recruited macrophages and ectopic osteoclasts along the vertebral column of Rankl-induced embryos compared to controls (**Supplementary Fig. S3**). Osteoclast numbers gradually declined after 2 dphs while new macrophages continued to migrate towards the vertebral column over the course of one week (data not shown). The total number of macrophages increased partly by steady-state hematopoiesis but also due to cell proliferation that occurred both in the AGM and around the vertebral column (**Supplementary Fig. S3; Movie S7**). Our findings suggest that in medaka ectopic Rankl expression triggers the activation of macrophages, stimulates their recruitment towards mineralized matrix in a directed manner and promotes their differentiation into osteoclasts.

**Macrophages reduce their dynamics during osteoclast maturation**

To quantitate the dynamics of macrophages during osteoclast differentiation, we analyzed time-lapse movies of Rankl-induced macrophages and osteoclasts recorded before and after differentiation. In an early phase at 4 hphs, macrophages in Rankl-induced embryos exhibited higher speed and displacement when compared to -Rankl controls. A more angular and less spherical cell morphology was observed in +Rankl macrophages, indicating their motile state (**Supplementary Fig. S1A**). However, at 34 hphs, the motility of differentiating macrophages became reduced as they started to express *ctsk:GFP* while undifferentiated macrophages remained highly mobile (**Supplementary Fig. S1B; Movie S4**). This suggests that the reduction of macrophage dynamics is a prerequisite for osteoclast differentiation, possibly to allow tighter attachment of forming osteoclasts to bone matrix for subsequent cell fusion to generate multinucleated osteoclasts.

**Depletion of macrophages prevents osteoclast formation**

As in mammals, also medaka macrophages differentiate into osteoclasts ^(3)^. We next determined whether osteoclasts also form in the absence of macrophages indicating possible alternative cellular sources. We depleted macrophages by Lipo-Clodronate (Lipo-Clo) injection into medaka embryos at 9 dpf and induced Rankl expression by heat shock at 10 dpf. In the absence of Rankl induction, Lipo-Clo treatment did not cause any obvious changes to bone development (**Fig. 3A-D**). After Rankl induction, at 1 and 2 dphs, Lipo-PBS control injected embryos showed abundant macrophage recruitment and osteoclast formation along the vertebral column as expected (**Fig. 3C-F**). In contrast, Lipo-Clo treatment resulted in a 75% ablation of macrophages (**Fig. 3C-E**). Only few macrophages were recruited to the vertebral column and osteoclast formation was strongly impaired (**Fig. 3C-F; Movies S5-S6**). The effect of macrophage depletion on bone protection was then assessed using Alizarin Red staining at 3 dphs. After Rankl induction, the bone matrix of Lipo-Clo injected embryos was efficiently protected with only few minor defects, which was in stark contrast to the severe lesions formed along the vertebral columns of Lipo-PBS injected embryos (**Fig. 3D**). This observation indicates that the integrity of a macrophage population is important for osteoclast formation.

**Transcriptome profiling reveals similarities between medaka and human osteoclasts**

The behavior and dynamics of macrophages change rapidly during differentiation into osteoclasts (**Movies S2-S4**). To identify molecular signatures underlying these changes, we performed RNAseq analysis and compared transcriptome profiles of FACS-purified macrophages (*mpeg1^+^*/*ctsk^-^*cells) with those of osteoclasts (*mpeg^+^*/*ctsk^+^* double positive cells) at 1 dphs. Analysis of differentially expressed genes (DEGs) revealed gene sets that are similarly regulated in human and mouse osteoclastogenesis. Conventional markers of osteoclastogenesis in mammals such as *trap, ctsk, siglec15, nfatc1, tgfb1, dap12* were highly upregulated in *mpeg^+^*/*ctsk^+^* double positive medaka cells, similar to the situation in human and mouse osteoclasts (**Table 1**). Our RNAseq datasets also indicated that genes encoding the medaka chemokine/cytokine receptors Cx3cr1, Il22ra2a and Il22ra2b were downregulated in medaka osteoclasts, but significance could only be validated by qPCR analysis for *il22ra2b* (**Supplementary Figs. S5-S7; Table 1**). The downregulation of cytokine receptor genes in our medaka model suggests a possible reduction of the motility of Rankl-induced macrophages as they differentiate into osteoclasts.

**Rankl-induced macrophages acquire a bone-remodeling signature**

Our DEG analysis identified more than one thousand genes that were either commonly or uniquely expressed in macrophages before and after differentiation into osteoclasts (**Fig. 4A**). Of the commonly expressed genes, 88 genes were significantly upregulated and 39 genes downregulated with adjusted P-values of < 0.05 (**Fig. 4A, C**). A KEGG analysis of DEGs revealed an enrichment of the terms phagosome, focal adhesion, extracellular membrane-receptor interaction, and cytokine-cytokine receptor interaction (**Fig. 4B**). Apart from genes for which the corresponding human orthologs have important functions in osteoclastogenesis, such as e.g. *fes*, *sh2dp1*, *socs1b*, *tspan5*, *mcoln3a* (**Fig. 4D; Table1**), we observed other highly regulated genes with so far uncharacterized functions in osteoclastogenesis. This includes upregulation of *emilin2a*, which is known to block cancer cell proliferation ^(29)^*,* and *sfrp2*, which functions to suppress normal osteoblast differentiation and exerts an anti-apoptotic effect ^(30,31)^. Among downregulated genes, we identified *gimap1-like*, which is essential for development of T- and B-lymphocytes , *rad18*, which is a key player in DNA damage tolerance mechanism ^(32,33)^, and *ogdh*, which is involved in glucose oxidation and cancer cells viability ^(34,35)^ (**Fig. 4D**). Next, we performed a Gene Ontology (GO) analysis using ShinyGO v0.61. Individual lists of up- and downregulated genes were converted into human gene IDs and UniProtKB IDs using BioMart (Ensembl.org). UniProtKB IDs were later used as input for the analysis.

The analysis of upregulated genes surprisingly showed a strong enrichment of pathways involved in chondrocyte and cartilage development, bone remodeling, and osteoblast differentiation. This included the collagen coding genes *col6a2*, *col12a1* as well as the secreted osteoblast differentiation inducers *tgfb1* and *sfrp2* (**Supplementary** **Fig. S4, Table 2**) ^(36)^. Consistent also with earlier studies that reported expression of collagen genes in macrophages ^(37-39)^, qPCR analysis confirmed the upregulation of *col6a2* and *col12a1* as well as integrin subunit beta 3 (*itgb3*) in Rankl-induced macrophages and validated our RNAseq analysis. Interestingly, also genes encoding the respective complexing subunits, i.e. *col6a1* and *col6a3* as well as *itga5* and *itga2.2*, were upregulated in osteoclasts (**Fig. S5B**).

A larger fraction of downregulated genes was immune-related and is known to be involved in the activation of the immune response of macrophages (**Supplementary** **Fig. S4A**). Our data suggest that macrophages are highly dynamic in regulating their gene expression, not only to favour bone resorbing activity, but also potentially to promote bone formation by secreting factors that trigger osteoblast differentiation.

**Macrophage recruitment and differentiation are impaired in *tnfa* mutants**

Treatment of mouse bone marrow-derived macrophages with the pro-inflammatory cytokine TNFa in the presence of M-CSF and RANKL stimulates osteoclast differentiation *in vitro* ^(15-17)^. We therefore investigated the requirement of this proinflammatory cytokine for macrophage recruitment and osteoclast differentiation *in vivo* in the medaka osteoporosis model. First, we performed quantitative PCR analysis to test whether *tnfa* transcription is affected by heat shock induction and Rankl overexpression. Compared to non-heat shocked controls, heat shocked -Rankl embryos showed a significant reduction of *tnfa* expression at 1, 2 and 3 days after heat-shock (**Supplementary Fig. S8A**). This is consistent with a temperature-induced reduction of *Tnfa* transcription in mouse macrophages ^(40)^. Importantly, however, Rankl induction (+Rankl) resulted in a significant upregulation of *tnfa* transcription suggesting that *tnfa* is involved in the osteoporotic response. To test this, we generated a *tnfa* medaka mutant using CRISPR/Cas9 (**Supplementary Fig. S8B-D**). Similar to *Tnfa* mouse mutants, which show normal development ^(41)^, stable CRISPR/Cas9 medaka *tnfa* mutants were viable and developed normally with no obvious alterations (data not shown). Mutants were crossed with *rankl:HSE:cfp/mpeg1:mCherry-F/ctsk:GFP* transgenic fish, and macrophage behaviour and osteoclast formation were tracked by live imaging as described above. In the absence of Rankl induction (-Rankl), *tnfa* mutants exhibited normal distribution and density of macrophages when compared to non-mutant siblings (**Fig. 5A-C**). Upon Rankl induction (+Rankl), *tnfa*^-/-^ mutant macrophages were recruited toward the vertebral column at 1 dphs, similarly to wild-type macrophages. Also, the total number of macrophages was initially similar in both wild-type and mutant embryos at 1 dphs. At 2 dphs, however, both total and recruited macrophage numbers were significantly reduced in homozygous mutants compared to that of wild-type siblings (**Fig. 5B, C**). Furthermore, *tnfa*^-/-^ mutants exhibited significantly reduced osteoclast numbers, while osteoclasts were abundant in Rankl-induced non-mutant siblings, (**Fig. 5A, D**). Next, to investigate whether Tnfa inhibition is sufficient to protect bone matrix from osteoporotic insult, Rankl-induced larvae were stained with calcein at 3 dphs to visualize mineralized matrix in the vertebral column. Upon Rankl induction, heterozygous *tnfa^+/-^* carriers showed similar bone defects as non-mutant siblings, which included bone loss in the neural arches and large lesions in the vertebral centra (**Fig. 5E**). Homozygous *tnfa^-/-^* embryos, in contrast, had fewer and smaller lesions in the centra indicative of improved bone integrity. This suggests that Tnfa is required for macrophage activation and osteoclast formation after Rankl induction *in vivo*.

To confirm this, we finally treated non-mutant embryos with the chemical inhibitor Pentoxifylline (PTX), which inhibits Tnfa synthesis. Importantly, in the absence of ectopic Rankl, PTX treatment did not cause any obvious changes to the distribution and density of macrophages (**Fig. 6A**). Moreover, upon Rankl induction, macrophages were recruited to the vertebral column to a similar extent in DMSO and PTX treated embryos, as quantitated at 1 and 2 dphs (**Fig. 6B, C**; white arrowheads). The total number of macrophages was slightly lower in PTX treated embryos at 1 dphs but increased to control levels at 2 dphs (**Fig. 6D**). Thus, other than in *tnfa^-/-^* mutants, macrophage recruitment was not affected by PTX, which we attribute to a lower efficacy of the drug compared to the genetic deletion. Importantly, however, osteoclast differentiation was strongly impaired in PTX treated embryos when compared to DMSO controls at both 1 and 2 dphs (**Fig. 6B, E**, green arrowheads). We then analyzed the resorptive activity of Rankl-induced osteoclasts in control and PTX treated embryos using Alizarin Red staining. In the absence of ectopic Rankl, both PTX and DMSO treated embryos showed normal mineralization with intact neural arches and vertebral bodies (**Supplementary** **Fig. S9**). Upon Rankl induction, DMSO control embryos exhibited resorption of neural arches and severe lesions in the centra. In contrast, PTX treatment resulted in considerable bone protection (**Supplementary** **Fig. S9**). Together, our data show that in medaka Tnfa is required for macrophage activation and osteoclast formation.

**DISCUSSION**

Small teleost fish, such as zebrafish and medaka, are widely used to model human bone diseases given their unique experimental advantages, as well as genetic and cellular similarities of medaka and human bone ^(42)^. In the present study, we used live imaging to demonstrate that in medaka, similar as in mammals ^(3)^, osteoclasts are derived from the monocyte/macrophage lineage in Rankl induced conditions. Our macrophage depletion data suggest that in medaka no alternative cellular sources exist for osteoclasts, and that the formation of osteoclasts requires a high integrity of the macrophage population. Transcriptome profiling of medaka macrophages and osteoclasts identified sets of genes that are similarly regulated in human and mouse osteoclastogenesis. It also revealed the induction of genes in differentiated macrophages that are known to be involved in osteoblast formation and bone remodeling. We show that Tnfa is essential for macrophage activation and osteoclast differentiation. Together, these results strengthen the medaka as a unique *in vivo* model accessible to live imaging and suitable for osteoporosis-related studies.

**Targeted migration and bone cell interaction of macrophages in medaka**

Macrophages express membrane-bound receptors that are needed for their recruitment to target sites in response to pathogens or instructive signals coming from other cell types. After Rankl induction in medaka, macrophages are recruited towards bone matrix, where they interact with osteoblasts and osteoblast progenitors and differentiate into osteoclasts (**Fig. 2**). Importantly, although massively recruited to the vertebral column, macrophages only accumulate at mineralized matrix of vertebral bodies but not at the non-mineralized intervertebral discs. This suggests that signals triggering macrophage recruitment could be released either by resident cells lining the bone surface, i.e. osteoblasts and osteoblast progenitors, or the matrix itself. Such signals appear to be absent from intervertebral discs or alternatively are distinct from those that induce chronic macrophage recruitment seen in intervertebral disc degeneration diseases ^(43)^. Previous studies indicated that osteoclasts can form from mouse spleen or bone marrow cells in the absence of bone matrix when co-cultured with osteoblasts and supplemented with calcitriol ^(^1 alpha, 25(OH)2 vitamin D3; ^44)^. This suggests that ossified matrix is dispensable for osteoclast formation and that osteoblasts instead produce the necessary differentiation signals. However, whether osteoblasts also release signals to recruit macrophages is still unknown. In mammals, osteoblasts express RANKL, which is important for osteoclast differentiation. In the medaka model, however, heat-shock induced Rankl is ubiquitous, yet only mineralized tissue attracts macrophages. This suggests that ectopic Rankl potentially acts directly on osteoblasts to trigger a release of attractants needed for macrophage recruitment. Consistent with this idea, we recently reported that medaka osteoblasts express the chemokine ligand Cxcl9l, which controls migration of macrophages towards bone matrix ^(45)^. Future characterization of osteoblasts is likely to identify additional factors controlling macrophage recruitment.

**Macrophages are the exclusive source for osteoclasts in medaka**

Macrophages are highly dynamic and have a variety of functions ranging from the clearance of foreign effectors to mediating communication across different cell types under developmental and pathological conditions ^(46,47)^. By time-lapse analysis, we found that all ectopically induced *ctsk*-expressing osteoclasts in the vertebral column originate from recruited *mpeg1* macrophages, suggesting that these macrophages are the exclusive cellular source for osteoclasts. Consistently, the transient depletion of *mpeg1* cells by Lipo-Clodronate significantly reduced osteoclast numbers upon Rankl induction (**Fig. 3**). The few remaining osteoclasts that escaped Lipo-Clodronate inhibition also originated from macrophages but not any other cell type. These results appear in contrast to earlier studies that reported persistent osteoclast formation in macrophage-depleted mice ^(48)^. This difference may imply that mice potentially have osteoclast precursors other than macrophages, and that these precursors are absent in medaka. The other possibility, however, is that the macrophage ablation in mice obtained by a Tamoxifen-inducible Cre-lox apoptosis system was incomplete allowing remaining macrophages to differentiate into osteoclasts, similar to our findings in medaka. Consistent with our findings, *Csf1r* mouse mutants exhibit a significant reduction of macrophage numbers in different tissues and also a decrease in osteoclast formation ^(49,50)^. Thus, other more efficient ablation techniques are needed to confirm that remaining osteoclasts in mice are differentiated from non-depleted macrophages or other cellular sources. In medaka, however, our live imaging approach confirmed that macrophages are the exclusive source for Rankl-induced osteoclasts.

**Tnfa is critical for macrophage recruitment and differentiation**

Earlier cell culture studies showed that TNFa induces osteoclast formation from mouse bone marrow-derived macrophages ^(15,17)^. Serum levels of TNFa are also increased in humans with low thyroid-stimulating hormone (TSH) levels and in mice carrying mutations in thyroid-stimulating hormone receptor (*Tshr^-/-^)^(51,52)^.* Both experience low bone mineral density and increased risk of bone fractures. Interestingly, in *Tshr^-/-^* mouse mutants, deletion of *Tnfa* partially rescued the low bone mass phenotype ^(53)^. Also, blocking TNFa by injecting a binding protein or deletion of *Tnfa* inhibited bone loss induced by ovariectomy ^(54,55)^. Conversely, TNFa addition promoted osteoclast survival and prevented apoptosis ^(56)^. Thus, these findings in mice strongly suggest an osteoclast promoting effect of TNFa. Consistent with this, we showed that also in the medaka osteoporosis model, genetic or chemical inhibition of *tnfa* expression reduced osteoclast formation upon Rankl induction (**Figs. 5 and 6**). *In vivo* imaging further revealed that in the absence of ectopic Rankl, Tnfa deficiency did not cause any obvious effects on macrophage populations. It was only after Rankl induction that *tnfa* mutants exhibited significantly reduced macrophage numbers overall and in the vertebral column. This strongly suggests that Tnfa selectively contributes to osteoclast differentiation under osteoporotic conditions but not in normal development. Consistently, our qPCR analysis revealed that *tnfa* transcription reached a maximum at 1 day after Rankl induction and was then reduced at 2 and 3 dphs (**Supplementary Fig. S8**). This implies that Tnfa may participate in the initial phase of macrophage recruitment. Its high levels at the beginning appear critical for macrophage differentiation into osteoclasts. Blocking TNFa has been an efficient treatment for rheumatoid arthritis, which is characterized by strong inflammation mediated by macrophages ^(13)^. Our results suggest that targeting TNFa signaling might possibly also be a promising therapeutic strategy for osteoporosis.

**Dynamic transcriptome changes in activated macrophages suggest multiple functions in bone remodeling**

A dynamic regulation of cytokines and their receptors is critical for immune cells to function accurately in response to foreign stimuli or micro-environmental changes ^(57)^. Uncontrolled overproduction of cytokines can lead to destructive chronic inflammation, which results in severe tissue defects ^(58)^. Certain cytokines receptors such as IL22Ra and Il20r have been shown to function as drivers for the migration and recruitment of different immune cell types including T and B cells, dendritic cells and neutrophils ^(59,60)^. The expression of these genes is also found upregulated in patients suffering from bone loss caused by rheumatoid arthritis and osteoporosis ^(61-63)^. Interestingly, our transcriptome profiling of FAC-sorted cells showed that these genes were downregulated in macrophages undergoing osteoclast differentiation (**Supplementary Fig. S5**). The reduced cytokine receptor expression coincided with a reduction of macrophage dynamics after they reached the bone matrix and started differentiating. We speculate that it is critical for macrophages to reduce motility before they attach tightly to bone matrix and form actin rings to facilitate bone resorption. While cytokine receptor expression was downregulated under osteoporotic conditions, pathways involved in chondrocyte and osteoblast differentiation surprisingly were upregulated in differentiated macrophages (**Table 2** and **Supplementary Fig. S5**). This could reflect a critical role of activated macrophages in bone cell-coupling, e.g. by promoting formation of chondrocytes and osteoblasts to achieve bone homeostasis. Our findings of upregulated *col6a1*, *col6a2*, *col6a3* and *col12a1b* expression in Rankl-induced macrophages are suggestive for a role in osteogenesis. Such a role was earlier proposed by Izu and colleagues, who described that collagens VI and XII form a complex to mediate osteoblast interaction during osteogenesis ^(64)^. In the medaka model, we also found upregulation of different integrin subunits and other adhesion molecules (**Supplementary Fig. S5**). This is in line with our observation that Rankl-induced macrophages interact dynamically with osteoblasts at the bone surface when maturing into osteoclasts (see Movie S7). We speculate that these matrix and adhesion proteins are critical for the tight interaction of pre-osteoclasts with osteoblasts and facilitate osteoclast differentiation. In an alternative scenario, our observation of upregulated osteogenic transcripts in osteoclasts could reflect the uptake of osteoblast-released exosomes, a process that was recently shown to occur in regenerating zebrafish scales ^(65)^. These osteoblast-derived exosomes could not only contain factors driving osteoclast differentiation, as demonstrated in ^(65)^, but also osteogenic mRNAs. However, we observed that the majority of upregulated osteogenic genes (i) is already expressed at basal levels in non-induced macrophages, and (ii) encode secreted or transmembrane proteins. This observation favours the idea of a non-cell autonomous role for activated macrophages in chondro- and osteogenesis. Clearly, further studies are needed to investigate the complex roles of macrophages in bone remodeling. Together, our study highlights the dynamic nature of macrophages and the complex intercellular interactions implicated in bone homeostasis.

**ACKNOWLEDGMENTS**

We thank Georges Lutfalla (Université Montpellier) for sharing the pTol2-mCherry-F plasmid. We also thank the CBIS confocal unit and the DBS fish facility for continued support. This project is supported by grants from the Singapore Ministry of Education (MOE2016-T2-2-086) and the National Research Foundation Singapore (NRF; NRF2017-NRF-ISF002-2671).

**AUTHOR CONTRIBUTIONS**

QTP, RL and CW designed the study, QTP, RL, WHT, NI, BC, MS and CW performed experiments, generated and analyzed data. QTP, RL and CW prepared the figures. QTP, RL, MS and CW drafted the manuscript, and QTP, MS and CW revised the manuscript text.

**REFERENCES**

1. Metchnikoff E. Untersuchungen ueber die mesodermalen Phagocyten einiger Wirbeltiere. Biologisches Centralblatt. 1883;3:560-5.

2. Wynn TA, Chawla A, Pollard JW. Macrophage biology in development, homeostasis and disease. Nature. Apr 25 2013;496(7446):445-55. Epub 2013/04/27.

3. Quinn JM, Neale S, Fujikawa Y, McGee JO, Athanasou NA. Human osteoclast formation from blood monocytes, peritoneal macrophages, and bone marrow cells. Calcif Tissue Int. Jun 1998;62(6):527-31. Epub 1998/06/20.

4. Jee WS, Nolan PD. ORIGIN OF OSTEOCLASTS FROM THE FUSION OF PHAGOCYTES. Nature. Oct 19 1963;200:225-6. Epub 1963/10/19.

5. Cho SW, Soki FN, Koh AJ, Eber MR, Entezami P, Park SI, et al. Osteal macrophages support physiologic skeletal remodeling and anabolic actions of parathyroid hormone in bone. Proc Natl Acad Sci U S A. Jan 28 2014;111(4):1545-50. Epub 2014/01/11.

6. Raggatt LJ, Wullschleger ME, Alexander KA, Wu AC, Millard SM, Kaur S, et al. Fracture healing via periosteal callus formation requires macrophages for both initiation and progression of early endochondral ossification. Am J Pathol. Dec 2014;184(12):3192-204. Epub 2014/10/07.

7. Sinder BP, Zweifler L, Koh AJ, Michalski MN, Hofbauer LC, Aguirre JI, et al. Bone Mass Is Compromised by the Chemotherapeutic Trabectedin in Association With Effects on Osteoblasts and Macrophage Efferocytosis. J Bone Miner Res. Oct 2017;32(10):2116-27. Epub 2017/06/11.

8. Nguyen-Chi M, Laplace-Builhe B, Travnickova J, Luz-Crawford P, Tejedor G, Lutfalla G, et al. TNF signaling and macrophages govern fin regeneration in zebrafish larvae. Cell Death Dis. Aug 10 2017;8(8):e2979. Epub 2017/08/11.

9. Sewter CP, Digby JE, Blows F, Prins J, O'Rahilly S. Regulation of tumour necrosis factor-alpha release from human adipose tissue in vitro. J Endocrinol. Oct 1999;163(1):33-8. Epub 1999/09/25.

10. Gideon HP, Phuah J, Junecko BA, Mattila JT. Neutrophils express pro- and anti-inflammatory cytokines in granulomas from Mycobacterium tuberculosis-infected cynomolgus macaques. Mucosal Immunology. 2019/11/01 2019;12(6):1370-81.

11. Charles KA, Kulbe H, Soper R, Escorcio-Correia M, Lawrence T, Schultheis A, et al. The tumor-promoting actions of TNF-alpha involve TNFR1 and IL-17 in ovarian cancer in mice and humans. The Journal of clinical investigation. 2009;119(10):3011-23. Epub 2009/09/08.

12. Kinne RW, Bräuer R, Stuhlmüller B, Palombo-Kinne E, Burmester GR. Macrophages in rheumatoid arthritis. Arthritis Res. 2000;2(3):189-202. Epub 2000/04/12.

13. Krieckaert CL, Nurmohamed MT, Wolbink G, Lems WF. Changes in bone mineral density during long-term treatment with adalimumab in patients with rheumatoid arthritis: a cohort study. Rheumatology (Oxford). Mar 2013;52(3):547-53. Epub 2012/12/12.

14. Al-Daghri NM, Aziz I, Yakout S, Aljohani NJ, Al-Saleh Y, Amer OE, et al. Inflammation as a contributing factor among postmenopausal Saudi women with osteoporosis. Medicine (Baltimore). Jan 2017;96(4):e5780. Epub 2017/01/26.

15. Lam J, Takeshita S, Barker JE, Kanagawa O, Ross FP, Teitelbaum SL. TNF-alpha induces osteoclastogenesis by direct stimulation of macrophages exposed to permissive levels of RANK ligand. J Clin Invest. Dec 2000;106(12):1481-8. Epub 2000/12/20.

16. Fuller K, Murphy C, Kirstein B, Fox SW, Chambers TJ. TNFalpha potently activates osteoclasts, through a direct action independent of and strongly synergistic with RANKL. Endocrinology. Mar 2002;143(3):1108-18. Epub 2002/02/28.

17. Kobayashi K, Takahashi N, Jimi E, Udagawa N, Takami M, Kotake S, et al. Tumor necrosis factor alpha stimulates osteoclast differentiation by a mechanism independent of the ODF/RANKL-RANK interaction. J Exp Med. Jan 17 2000;191(2):275-86. Epub 2000/01/19.

18. To TT, Witten PE, Renn J, Bhattacharya D, Huysseune A, Winkler C. Rankl-induced osteoclastogenesis leads to loss of mineralization in a medaka osteoporosis model. Development. Jan 2012;139(1):141-50.

19. Phan QT, Tan WH, Liu R, Sundaram S, Buettner A, Kneitz S, et al. Cxcl9l and Cxcr3.2 regulate recruitment of osteoclast progenitors to bone matrix in a medaka osteoporosis model. Proc Natl Acad Sci U S A. Jul 27 2020. Epub 2020/07/29.

20. Rembold M, Lahiri K, Foulkes NS, Wittbrodt J. Transgenesis in fish: efficient selection of transgenic fish by co-injection with a fluorescent reporter construct. Nat Protoc. 2006;1(3):1133-9.

21. Stemmer M, Thumberger T, Del Sol Keyer M, Wittbrodt J, Mateo JL. CCTop: An Intuitive, Flexible and Reliable CRISPR/Cas9 Target Prediction Tool. PLoS One. 2015;10(4):e0124633. Epub 2015/04/25.

22. Renn J, Winkler C. Osterix-mCherry transgenic medaka for in vivo imaging of bone formation. Dev Dyn. Jan 2009;238(1):241-8. Epub 2008/12/20.

23. Phan QT, Sipka T, Gonzalez C, Levraud JP, Lutfalla G, Nguyen-Chi M. Neutrophils use superoxide to control bacterial infection at a distance. PLoS Pathog. Jul 2018;14(7):e1007157. Epub 2018/07/18.

24. Buettner A, Sundaram S, Vyas H, Yu T, Mathavan S, Winkler C. Fluorescence-activated cell sorting (FACS) of osteoblasts and osteoclasts for RNA sequencing in a medaka, Oryzias latipes (Temming & Schlegel, 1846), osteoporosis model. Journal of Applied Ichthyology. 2018;34(2):481-8.

25. Larkin MA, Blackshields G, Brown NP, Chenna R, McGettigan PA, McWilliam H, et al. Clustal W and Clustal X version 2.0. Bioinformatics. Nov 1 2007;23(21):2947-8. Epub 2007/09/12.

26. Kumar S, Stecher G, Li M, Knyaz C, Tamura K. MEGA X: Molecular Evolutionary Genetics Analysis across Computing Platforms. Mol Biol Evol. Jun 1 2018;35(6):1547-9. Epub 2018/05/04.

27. Nguyen NTT, Vincens P, Roest Crollius H, Louis A. Genomicus 2018: karyotype evolutionary trees and on-the-fly synteny computing. Nucleic Acids Res. Jan 4 2018;46(D1):D816-D22. Epub 2017/11/01.

28. Hsu YH, Chen WY, Chan CH, Wu CH, Sun ZJ, Chang MS. Anti-IL-20 monoclonal antibody inhibits the differentiation of osteoclasts and protects against osteoporotic bone loss. J Exp Med. Aug 29 2011;208(9):1849-61. Epub 2011/08/17.

29. Andreuzzi E, Fejza A, Capuano A, Poletto E, Pivetta E, Doliana R, et al. Deregulated expression of Elastin Microfibril Interfacer 2 (EMILIN2) in gastric cancer affects tumor growth and angiogenesis. Matrix Biology Plus. 2020/05/01/ 2020;6-7:100029.

30. Kim H, Yoo S, Zhou R, Xu A, Bernitz JM, Yuan Y, et al. Oncogenic role of SFRP2 in p53-mutant osteosarcoma development via autocrine and paracrine mechanism. Proceedings of the National Academy of Sciences. 2018;115(47):E11128.

31. Zhang Z, Deb A, Zhang Z, Pachori A, He W, Guo J, et al. Secreted frizzled related protein 2 protects cells from apoptosis by blocking the effect of canonical Wnt3a. J Mol Cell Cardiol. 2009;46(3):370-7. Epub 2008/12/09.

32. Saunders A, Webb LMC, Janas ML, Hutchings A, Pascall J, Carter C, et al. Putative GTPase GIMAP1 is critical for the development of mature B and T lymphocytes. Blood. 2010;115(16):3249-57.

33. Notenboom V, Hibbert RG, van Rossum-Fikkert SE, Olsen JV, Mann M, Sixma TK. Functional characterization of Rad18 domains for Rad6, ubiquitin, DNA binding and PCNA modification. Nucleic Acids Res. 2007;35(17):5819-30. Epub 2007/08/24.

34. Araújo WL, Trofimova L, Mkrtchyan G, Steinhauser D, Krall L, Graf A, et al. On the role of the mitochondrial 2-oxoglutarate dehydrogenase complex in amino acid metabolism. Amino Acids. Feb 2013;44(2):683-700. Epub 2012/09/18.

35. Bunik VI, Mkrtchyan G, Grabarska A, Oppermann H, Daloso D, Araujo WL, et al. Inhibition of mitochondrial 2-oxoglutarate dehydrogenase impairs viability of cancer cells in a cell-specific metabolism-dependent manner. Oncotarget. 2016;7(18):26400-21.

36. Horwood NJ, Elliott J, Martin TJ, Gillespie MT. IL-12 alone and in synergy with IL-18 inhibits osteoclast formation in vitro. J Immunol. Apr 15 2001;166(8):4915-21. Epub 2001/04/06.

37. Schnoor M, Cullen P, Lorkowski J, Stolle K, Robenek H, Troyer D, et al. Production of type VI collagen by human macrophages: a new dimension in macrophage functional heterogeneity. J Immunol. Apr 15 2008;180(8):5707-19. Epub 2008/04/09.

38. Nakamura I, Pilkington MF, Lakkakorpi PT, Lipfert L, Sims SM, Dixon SJ, et al. Role of alpha(v)beta(3) integrin in osteoclast migration and formation of the sealing zone. J Cell Sci. Nov 1999;112 ( Pt 22):3985-93. Epub 1999/11/05.

39. Helfrich MH, Nesbitt SA, Lakkakorpi PT, Barnes MJ, Bodary SC, Shankar G, et al. Beta 1 integrins and osteoclast function: involvement in collagen recognition and bone resorption. Bone. Oct 1996;19(4):317-28. Epub 1996/10/01.

40. Singh IS, He JR, Calderwood S, Hasday JD. A high affinity HSF-1 binding site in the 5'-untranslated region of the murine tumor necrosis factor-alpha gene is a transcriptional repressor. J Biol Chem. Feb 15 2002;277(7):4981-8. Epub 2001/12/06.

41. Marino MW, Dunn A, Grail D, Inglese M, Noguchi Y, Richards E, et al. Characterization of tumor necrosis factor-deficient mice. Proc Natl Acad Sci U S A. 1997;94(15):8093-8.

42. Lleras-Forero L, Winkler C, Schulte-Merker S. Zebrafish and medaka as models for biomedical research of bone diseases. Dev Biol. Jul 17 2019. Epub 2019/07/22.

43. Nakazawa KR, Walter BA, Laudier DM, Krishnamoorthy D, Mosley GE, Spiller KL, et al. Accumulation and localization of macrophage phenotypes with human intervertebral disc degeneration. Spine J. Feb 2018;18(2):343-56. Epub 2017/10/17.

44. Takahashi N, Yamana H, Yoshiki S, Roodman GD, Mundy GR, Jones SJ, et al. Osteoclast-like cell formation and its regulation by osteotropic hormones in mouse bone marrow cultures. Endocrinology. Apr 1988;122(4):1373-82. Epub 1988/04/01.

45. Phan QT, Tan WH, Liu R, Sundaram S, Buettner A, Kneitz S, et al. Cxcl9l and Cxcr3.2 regulate recruitment of osteoclast progenitors to bone matrix in a medaka osteoporosis model. Proceedings of the National Academy of Sciences. 2020:202006093.

46. Tirone M, Giovenzana A, Vallone A, Zordan P, Sormani M, Nicolosi PA, et al. Severe Heterotopic Ossification in the Skeletal Muscle and Endothelial Cells Recruitment to Chondrogenesis Are Enhanced by Monocyte/Macrophage Depletion. Front Immunol. 2019;10:1640. Epub 2019/08/10.

47. Laplace-Builhe B, Nguyen-Chi M, Travnickova J, Luz-Crawford P, Tejedor G, Kissa K, et al. A2.2 Pro-inflammatory macrophages mediated TNF-alpha signalling is required for caudal fin regenerationin zebrafish larvae. Annals of the Rheumatic Diseases. 2015;74(Suppl 1):A16-A.

48. Burnett SH, Kershen EJ, Zhang J, Zeng L, Straley SC, Kaplan AM, et al. Conditional macrophage ablation in transgenic mice expressing a Fas-based suicide gene. J Leukoc Biol. Apr 2004;75(4):612-23. Epub 2004/01/17.

49. Pridans C, Raper A, Davis GM, Alves J, Sauter KA, Lefevre L, et al. Pleiotropic Impacts of Macrophage and Microglial Deficiency on Development in Rats with Targeted Mutation of the Csf1r Locus. J Immunol. Nov 1 2018;201(9):2683-99. Epub 2018/09/27.

50. Rojo R, Raper A, Ozdemir DD, Lefevre L, Grabert K, Wollscheid-Lengeling E, et al. Deletion of a Csf1r enhancer selectively impacts CSF1R expression and development of tissue macrophage populations. Nat Commun. Jul 19 2019;10(1):3215. Epub 2019/07/22.

51. Lee WY, Kang MI, Oh KW, Oh ES, Baek KH, Lee KW, et al. Relationship between circulating cytokine levels and thyroid function following bone marrow transplantation. Bone Marrow Transplantation. 2004/01/01 2004;33(1):93-8.

52. Hase H, Ando T, Eldeiry L, Brebene A, Peng Y, Liu L, et al. TNFα mediates the skeletal effects of thyroid-stimulating hormone. Proceedings of the National Academy of Sciences. 2006;103(34):12849-54.

53. Sun L, Zhu LL, Lu P, Yuen T, Li J, Ma R, et al. Genetic confirmation for a central role for TNFalpha in the direct action of thyroid stimulating hormone on the skeleton. Proc Natl Acad Sci U S A. Jun 11 2013;110(24):9891-6. Epub 2013/05/30.

54. Kimble RB, Bain S, Pacifici R. The functional block of TNF but not of IL-6 prevents bone loss in ovariectomized mice. J Bone Miner Res. Jun 1997;12(6):935-41. Epub 1997/06/01.

55. Zhu S, He H, Gao C, Luo G, Xie Y, Wang H, et al. Ovariectomy-induced bone loss in TNFα and IL6 gene knockout mice is regulated by different mechanisms. 2018;60(3):185.

56. Glantschnig H, Fisher JE, Wesolowski G, Rodan GA, Reszka AA. M-CSF, TNFalpha and RANK ligand promote osteoclast survival by signaling through mTOR/S6 kinase. Cell Death Differ. Oct 2003;10(10):1165-77. Epub 2003/09/23.

57. Chi H, Barry SP, Roth RJ, Wu JJ, Jones EA, Bennett AM, et al. Dynamic regulation of pro- and anti-inflammatory cytokines by MAPK phosphatase 1 (MKP-1) in innate immune responses. Proceedings of the National Academy of Sciences. 2006;103(7):2274-9.

58. Zwerina J, Redlich K, Polzer K, Joosten L, Krönke G, Distler J, et al. TNF-induced structural joint damage is mediated by IL-1. Proceedings of the National Academy of Sciences of the United States of America. 2007;104(28):11742-7. Epub 2007/07/03.

59. Brandes M, Legler DF, Spoerri B, Schaerli P, Moser B. Activation-dependent modulation of B lymphocyte migration to chemokines. Int Immunol. Sep 2000;12(9):1285-92. Epub 2000/09/01.

60. Bech R, Jalilian B, Agger R, Iversen L, Erlandsen M, Otkjaer K, et al. Interleukin 20 regulates dendritic cell migration and expression of co-stimulatory molecules. Mol Cell Ther. 2016;4:1. Epub 2016/01/29.

61. Kokkonen H, Soderstrom I, Rocklov J, Hallmans G, Lejon K, Rantapaa Dahlqvist S. Up-regulation of cytokines and chemokines predates the onset of rheumatoid arthritis. Arthritis Rheum. Feb 2010;62(2):383-91. Epub 2010/01/30.

62. Hustmyer FG, Walker E, Yu XP, Girasole G, Sakagami Y, Peacock M, et al. Cytokine production and surface antigen expression by peripheral blood mononuclear cells in postmenopausal osteoporosis. J Bone Miner Res. Jan 1993;8(1):51-9. Epub 1993/01/01.

63. Kragstrup TW, Andersen T, Heftdal LD, Hvid M, Gerwien J, Sivakumar P, et al. The IL-20 Cytokine Family in Rheumatoid Arthritis and Spondyloarthritis. Front Immunol. 2018;9:2226-.

64. Izu Y, Ezura Y, Koch M, Birk DE, Noda M. Collagens VI and XII form complexes mediating osteoblast interactions during osteogenesis. Cell Tissue Res. 2016;364(3):623-35. Epub 2016/01/12.

65. Kobayashi-Sun J, Yamamori S, Kondo M, Kuroda J, Ikegame M, Suzuki N, et al. Uptake of osteoblast-derived extracellular vesicles promotes the differentiation of osteoclasts in the zebrafish scale. Commun Biol. Apr 23 2020;3(1):190. Epub 2020/04/25.

66. Ek-Rylander B, Flores M, Wendel M, Heinegard D, Andersson G. Dephosphorylation of osteopontin and bone sialoprotein by osteoclastic tartrate-resistant acid phosphatase. Modulation of osteoclast adhesion in vitro. J Biol Chem. May 27 1994;269(21):14853-6. Epub 1994/05/27.

67. Kameda Y, Takahata M, Komatsu M, Mikuni S, Hatakeyama S, Shimizu T, et al. Siglec-15 regulates osteoclast differentiation by modulating RANKL-induced phosphatidylinositol 3-kinase/Akt and Erk pathways in association with signaling Adaptor DAP12. J Bone Miner Res. Dec 2013;28(12):2463-75. Epub 2013/05/17.

68. Kim K, Lee SH, Ha Kim J, Choi Y, Kim N. NFATc1 induces osteoclast fusion via up-regulation of Atp6v0d2 and the dendritic cell-specific transmembrane protein (DC-STAMP). Mol Endocrinol. Jan 2008;22(1):176-85. Epub 2007/09/22.

69. Winslow MM, Pan M, Starbuck M, Gallo EM, Deng L, Karsenty G, et al. Calcineurin/NFAT signaling in osteoblasts regulates bone mass. Dev Cell. Jun 2006;10(6):771-82. Epub 2006/06/03.

70. Yu X, Huang Y, Collin-Osdoby P, Osdoby P. Stromal cell-derived factor-1 (SDF-1) recruits osteoclast precursors by inducing chemotaxis, matrix metalloproteinase-9 (MMP-9) activity, and collagen transmigration. J Bone Miner Res. Aug 2003;18(8):1404-18. Epub 2003/08/22.

71. Erkhembaatar M, Gu DR, Lee SH, Yang YM, Park S, Muallem S, et al. Lysosomal Ca(2+) Signaling is Essential for Osteoclastogenesis and Bone Remodeling. J Bone Miner Res. Feb 2017;32(2):385-96. Epub 2016/09/03.

72. Zhang T, Kastrenopoulou A, Larrouture Q, Athanasou NA, Knowles HJ. Angiopoietin-like 4 promotes osteosarcoma cell proliferation and migration and stimulates osteoclastogenesis. BMC Cancer. May 8 2018;18(1):536. Epub 2018/05/10.

73. Swales C, Athanasou NA, Knowles HJ. Angiopoietin-like 4 is over-expressed in rheumatoid arthritis patients: association with pathological bone resorption. PLoS One. 2014;9(10):e109524. Epub 2014/10/08.

74. Takeda Y, Tachibana I, Miyado K, Kobayashi M, Miyazaki T, Funakoshi T, et al. Tetraspanins CD9 and CD81 function to prevent the fusion of mononuclear phagocytes. J Cell Biol. Jun 9 2003;161(5):945-56. Epub 2003/06/11.

75. Paloneva J, Mandelin J, Kiialainen A, Bohling T, Prudlo J, Hakola P, et al. DAP12/TREM2 deficiency results in impaired osteoclast differentiation and osteoporotic features. J Exp Med. Aug 18 2003;198(4):669-75. Epub 2003/08/20.

76. Kwak HB, Lee SW, Jin HM, Ha H, Lee SH, Takeshita S, et al. Monokine induced by interferon-gamma is induced by receptor activator of nuclear factor kappa B ligand and is involved in osteoclast adhesion and migration. Blood. Apr 1 2005;105(7):2963-9. Epub 2004/12/09.

77. Bossard MJ, Tomaszek TA, Thompson SK, Amegadzie BY, Hanning CR, Jones C, et al. Proteolytic activity of human osteoclast cathepsin K. Expression, purification, activation, and substrate identification. J Biol Chem. May 24 1996;271(21):12517-24. Epub 1996/05/24.

78. Chang EJ, Ha J, Oerlemans F, Lee YJ, Lee SW, Ryu J, et al. Brain-type creatine kinase has a crucial role in osteoclast-mediated bone resorption. Nat Med. Sep 2008;14(9):966-72. Epub 2008/08/30.

79. Marino N, Marshall JC, Collins JW, Zhou M, Qian Y, Veenstra T, et al. Nm23-h1 binds to gelsolin and inactivates its actin-severing capacity to promote tumor cell motility and metastasis. Cancer Res. Oct 1 2013;73(19):5949-62. Epub 2013/08/14.

80. Yu X, Huang Y, Collin-Osdoby P, Osdoby P. CCR1 chemokines promote the chemotactic recruitment, RANKL development, and motility of osteoclasts and are induced by inflammatory cytokines in osteoblasts. J Bone Miner Res. Dec 2004;19(12):2065-77. Epub 2004/11/13.

81. Ohishi M, Matsumura Y, Aki D, Mashima R, Taniguchi K, Kobayashi T, et al. Suppressors of cytokine signaling-1 and -3 regulate osteoclastogenesis in the presence of inflammatory cytokines. J Immunol. Mar 1 2005;174(5):3024-31. Epub 2005/02/25.

82. Yogo K, Mizutamari M, Mishima K, Takenouchi H, Ishida-Kitagawa N, Sasaki T, et al. Src homology 2 (SH2)-containing 5'-inositol phosphatase localizes to podosomes, and the SH2 domain is implicated in the attenuation of bone resorption in osteoclasts. Endocrinology. Jul 2006;147(7):3307-17. Epub 2006/04/08.

83. Hellwig S, Miduturu CV, Kanda S, Zhang J, Filippakopoulos P, Salah E, et al. Small-molecule inhibitors of the c-Fes protein-tyrosine kinase. Chem Biol. Apr 20 2012;19(4):529-40. Epub 2012/04/24.

84. Maruyama K, Uematsu S, Kondo T, Takeuchi O, Martino MM, Kawasaki T, et al. Strawberry notch homologue 2 regulates osteoclast fusion by enhancing the expression of DC-STAMP. J Exp Med. Sep 23 2013;210(10):1947-60. Epub 2013/08/28.

85. Zhao X, Cui P, Hu G, Wang C, Jiang L, Zhao J, et al. PIP5k1beta controls bone homeostasis through modulating both osteoclast and osteoblast differentiation. J Mol Cell Biol. Apr 16 2019. Epub 2019/04/16.

86. Oh J, Kim JY, Kim HS, Oh JC, Cheon YH, Park J, et al. Progranulin and a five transmembrane domain-containing receptor-like gene are the key components in receptor activator of nuclear factor kappaB (RANK)-dependent formation of multinucleated osteoclasts. J Biol Chem. Jan 23 2015;290(4):2042-52. Epub 2014/11/20.

87. Ohmae S, Noma N, Toyomoto M, Shinohara M, Takeiri M, Fuji H, et al. Actin-binding protein coronin 1A controls osteoclastic bone resorption by regulating lysosomal secretion of cathepsin K. Sci Rep. Mar 16 2017;7:41710. Epub 2017/03/17.

88. Moon YJ, Yun C-Y, Choi H, Kim JR, Park B-H, Cho E-S. Osterix regulates corticalization for longitudinal bone growth via integrin β3 expression. Experimental & Molecular Medicine. 2018/07/18 2018;50(7):80.

89. Smeriglio P, Dhulipala L, Lai JH, Goodman SB, Dragoo JL, Smith RL, et al. Collagen VI Enhances Cartilage Tissue Generation by Stimulating Chondrocyte Proliferation. Tissue Engineering Part A. 2015/02/01 2014;21(3-4):840-9.

90. Rice R, Rice DP, Olsen BR, Thesleff I. Progression of calvarial bone development requires Foxc1 regulation of Msx2 and Alx4. Dev Biol. Oct 1 2003;262(1):75-87. Epub 2003/09/27.

91. Jin L, Cao Y, Yu G, Wang J, Lin X, Ge L, et al. SFRP2 enhances the osteogenic differentiation of apical papilla stem cells by antagonizing the canonical WNT pathway. Cell Mol Biol Lett. 2017;22:14-.

92. Kimura H, Kwan KM, Zhang Z, Deng JM, Darnay BG, Behringer RR, et al. Cthrc1 is a positive regulator of osteoblastic bone formation. PLoS One. Sep 9 2008;3(9):e3174. Epub 2008/09/10.

93. Kwon HS, Johnson TV, Tomarev SI. Myocilin stimulates osteogenic differentiation of mesenchymal stem cells through mitogen-activated protein kinase signaling. The Journal of biological chemistry. 2013;288(23):16882-94. Epub 2013/04/29.

94. Izu Y, Sun M, Zwolanek D, Veit G, Williams V, Cha B, et al. Type XII collagen regulates osteoblast polarity and communication during bone formation. The Journal of cell biology. 2011;193(6):1115-30.

95. Tuli R, Tuli S, Nandi S, Huang X, Manner PA, Hozack WJ, et al. Transforming growth factor-beta-mediated chondrogenesis of human mesenchymal progenitor cells involves N-cadherin and mitogen-activated protein kinase and Wnt signaling cross-talk. J Biol Chem. Oct 17 2003;278(42):41227-36. Epub 2003/08/02.

96. Maumus M, Manferdini C, Toupet K, Chuchana P, Casteilla L, Gachet M, et al. Thrombospondin-1 Partly Mediates the Cartilage Protective Effect of Adipose-Derived Mesenchymal Stem Cells in Osteoarthritis. Frontiers in immunology. 2017;8:1638-.

97. Zhang Y, Sheu T-j, Hoak D, Shen J, Hilton MJ, Zuscik MJ, et al. CCN1 Regulates Chondrocyte Maturation and Cartilage Development. Journal of bone and mineral research : the official journal of the American Society for Bone and Mineral Research. 2016;31(3):549-59.

98. Lindsey RC, Xing W, Pourteymoor S, Godwin C, Gow A, Mohan S. Novel Role for Claudin-11 in the Regulation of Osteoblasts via Modulation of ADAM10-Mediated Notch Signaling. Journal of Bone and Mineral Research. 2019;34(10):1910-22.

**Table 1:** **Selected regulated medaka genes with orthologs involved in mammalian osteoporosis and bone loss.**

| **Gene** | **Description** | **Reads**  **Mφ** | **Reads**  **OC** | **Log2 FC** | **p-adj** | **Known function in mammalian bone** | **Ref** |
| --- | --- | --- | --- | --- | --- | --- | --- |
| *trap* | acid phosphatase 5a, tartrate resistant | 1766 | 62181 | 5.1 | 0.000 | Modulation of osteoclast adhesion and resorption | ^(66)^ |
| *siglec15* | sialic acid binding Ig like lectin 15 | 131 | 3833 | 4.9 | 0.000 | Osteoclast differentiation | ^(67)^ |
| *nfatc1* | nuclear factor of activated T cells 1 | 139 | 3218 | 4.5 | 0.001 | Osteoclast fusion | ^(68,69)^ |
| *a-sdf1a/cxcl12* | stromal cell-derived factor 1a | 61 | 1233 | 4.3 | 0.001 | Osteoclast precursor recruitment | ^(70)^ |
| *mcoln3a* | mucolipin 3a | 72 | 1276 | 4.2 | 0.050 | Triggering Ca^2+^ release and influx during osteoclastogenesis and bone remodeling | ^(71)^ |
| *angptl7* | angiopoietin-like 7 | 176 | 3074 | 4.1 | 0.000 | Osteoclast stimulation. | ^(72,73)^ |
| *tspan5* | tetraspanin 5 | 137 | 1213 | 3.2 | 0.043 | Control of osteoclast fusion | ^(74)^ |
| *dap12/ tyrobp* | dap12/TYRO protein tyrosine kinase binding protein | 642 | 4642 | 2.9 | 0.000 | Osteoclast differentiation and function | ^(75)^ |
| *LOC101158504* | C-X-C motif chemokine 9 | 520 | 2681 | 2.3 | 0.01 | Produced by osteoblasts and osteoclasts; induces osteoclast migration and adhesion. | ^(76)^ |
| *ctsk* | cathepsin K | 53392 | 164567 | 1.6 | 0.000 | Protease for osteoclast resorptive activity | ^(77)^ |
| *ckba* | creatine kinase B | 4260 | 10737 | 1.3 | 0.025 | Osteoclast resorption; crucial for actin ring formation and bone resorption. | ^(78)^ |
| *gsna* | gelsolin a | 7076 | 16649 | 1.2 | 0.016 | Assembly/disassembly of actin filaments in osteoclast podosomes; cell migration | ^(79)^ |
| *LOC101163603* | C-C chemokine receptor type 1 | 341 | 0 | inf | 0.020 | Osteoclast recruitment, cell motility | ^(80)^ |
| *socs1b* | suppressor of cytokine signaling 1b | 348 | 1 | -9.4 | 0.020 | Osteoclast formation by cytokine modulation | ^(81)^ |
| *sh2dp1* | SH2 domain-containing protein 1B | 511 | 3 | -7.4 | 0.010 | Control of osteoclast resorptive activity and fusion | ^(82)^ |
| *fes* | FES proto-oncogene, tyrosine kinase | 823 | 20 | -5.4 | 0.000 | Osteoclast differentiation | ^(83)^ |
| *LOC105355996* | interleukin-20 receptor subunit alpha | 3043 | 205 | -3.9 | 0.000 | Osteoclast differentiation | ^(28)^ |
| *sbno2* | strawberry notch homolog 2 | 1559 | 188 | -3.1 | 0.020 | Osteoclast fusion | ^(84)^ |
| *pip5k1ba* | phosphatidylinositol-4-phosphate 5-kinase, type I, beta a | 2591 | 427 | -2.6 | 0.000 | Modulation of osteoblast and osteoclast differentiation | ^(85)^ |
| *grna* | granulin a | 17770 | 7107 | -1.3 | 0.010 | Osteoclast formation | ^(86)^ |
| *coro1a* | coronin, actin binding protein, 1A | 29530 | 13890 | -1.1 | 0.020 | Regulation of lysosomal secretion of cathepsin K | ^(87)^ |

**Table 2:** **Upregulation of osteoblast/chondrocyte promoting genes in medaka osteoclasts.**

| **Gene** | **Description** | **Reads**  **Mφ** | **Reads**  **OC** | **Log2**  **FC** | **P-adj** | **Protein localization** | **Role in bone/cartilage formation** | **Ref** |
| --- | --- | --- | --- | --- | --- | --- | --- | --- |
| *itgb3* | integrin subunit beta 3 | 0 | 3016 | inf* | 0.000 | Transmembrane | Corticalization for bone growth | ^(88)^ |
| *col6a2* | collagen alpha-2(VI) chain | 0 | 880 | inf | 0.000 | Secreted | Chondrocyte proliferation and cartilage generation | ^(89)^ |
| *foxc1* | forkhead box C1-A | 0 | 362 | inf | 0.007 | Nuclear | Osteoprogenitor proliferation; osteogenesis | ^(90)^ |
| *sfrp2* | secreted frizzled related protein 2 | 3 | 854 | 8 | 0.000 | Secreted | Osteogenic differentiation | ^(91)^ |
| *cthrc1* | collagen triple helix repeat containing 1 | 90 | 6684 | 6.21 | 0.000 | Secreted | Osteoblast proliferation, bone cell coupling | ^(92)^ |
| *myoc* | myocilin | 61 | 1070 | 4.14 | 0.009 | Secreted | Osteoblast differentiation; bone remodeling | ^(93)^ |
| *col12a1* | collagen alpha-1(XII) chain | 83 | 1038 | 3.64 | 0.027 | Secreted | Osteoblast polarity, bone matrix | ^(94)^ |
| *tgfb1* | transforming growth factor beta 1 | 367 | 3291 | 3.16 | 0.000 | Secreted | Mesenchymal progenitor differentiation; chondrogenesis | ^(95)^ |
| *thbs1* | thrombospondin 1 | 426 | 2689 | 2.66 | 0.003 | Secreted | Mesenchymal progenitor differentiation; chondrogenesis | ^(96)^ |
| *ccn1* | cellular communication network factor 1 | 2434 | 14444 | 2.57 | 0.000 | Secreted | Chondrocyte maturation | ^(97)^ |
| *cldn11* | claudin-11 | 440 | 2538 | 2.53 | 0.006 | Transmembrane | Osteoblast differentiation via Notch | ^(98)^ |

***infinite**

**FIGURE LEGENDS**

**Figure 1.** **Generation of the *mpeg1:mCherry-F* reporter line.**

**A**. A farnesylated mCherry reporter is driven by a 2.1 kb *mpeg1* promoter and flanked by *I-SceI* sites. **B**. At ten days post fertilization (dpf), *mCherry-F* labelled macrophages are distributed throughout the body except for the notochord and spinal cord. **C-F**. In-situ hybridizations of 10 dpf embryos with a *mpeg1* antisense probe on the transverse section of the anterior region (blue dotted line in B) (C), and a longitudinal section around the AGM-CHT area (F). D shows high magnification of liver tissue boxed in C, whole-mount fin region (E) arrows point to Mpeg1 positive signals, patterns are similar to the mCherry reporter signals. **G.** In-situ hybridizations with a *mpeg1* sense probe on a control whole-mount fin. NA: neural arch; NC: notochord; AGM: aorta gonad mesonephros; CHT: caudal hematopoietic tissue; CF: caudal fin; H: heart. Scale bar: 1 mm (in B); 200 μm (in C); 100 μm (in E-F-G).

**Figure 2.** **Macrophage recruitment and differentiation into osteoclasts in the medaka vertebral column.**

**A**. Experimental scheme. *rankl:HSE:cfp/mpeg1:mCherry-F/ctsk:GFP* transgenic medaka embryos were heat-shocked to induce Rankl expression and subjected to confocal time-lapse imaging. **B-D**. Still images extracted from movies recorded during the indicated time frame (hours) post heat-shock (hphs). Macrophages are recruited towards the vertebral column as early as 2 hphs and begin to differentiate into *ctsk* osteoclasts around 18 hphs, starting at the anterior region of the vertebral column. At 40 hphs, most of the recruited macrophages have differentiated into osteoclasts while later recruited macrophages are located around the vertebral column to perform phagocytosis of dying cells. Blue dotted box in **A** shows imaged area shown in **B-D**; white dotted boxes in **B** depict vertebral bodies. Mφ: macrophage; OC: osteoclast. Time: hh:mm:ss. Scale bar: 50 μm.

**Figure 3. Macrophage depletion prevents osteoclast formation.**

**A.** Experimental scheme. *rankl:HSE:cfp/mpeg1:mCherry-F/ctsk:GFP* transgenic embryos are injected with Lipo-Clodronate or Lipo-PBS at 9 dpf. At 10 dpf, embryos with good macrophage depletion are selected based on the quantity and morphology of mCherry signals, and then subjected to heat induction. **B**. Lipo-Clodronate injection causes macrophage death at 1 day after injection (round cells pointed by cyan arrowheads), while Lipo-PBS injected embryos show normal macrophage morphology and distribution (white arrowheads). **C**. Lipo-PBS injected control embryos show normal macrophage recruitment and osteoclast differentiation at 1 dphs (white arrowheads). In macrophage-ablated embryos, osteoclast formation along the trunk is notably inhibited (cyan arrowheads). **D**. At 2 dphs, abundant osteoclasts have formed in Lipo-PBS injected embryos (white arrowheads), while only single osteoclasts are seen in the anterior region of Lipo-Clo injected embryos (white arrows). Alizarin Red staining of mineralized bone matrix at 3 dphs reveals normal bone development in both Lipo-PBS and Lipo-Clodronate treated embryos in the absence of Rankl induction. Upon Rankl induction, control larvae have severe lesions in the mineralized matrix (black arrows), while Lipo-Clodronate injected embryos show minor or no defects (blue arrows). Insets show individual vertebral bodies at higher magnification. **E-F**. Quantification of cell numbers shows efficient depletion of macrophages and inhibition of osteoclast formation in Lipo-Clodronate treated embryos. N_Lipo-PBS larvae_ = 5-14, N_Lipo-Clo larvae_ = 6-19, from three independent experiments. Mφ: macrophage; OC: osteoclast. Scale bar: 200 μm.

**Figure 4.** **Transcriptome profiles of medaka macrophages and osteoclasts.**

**A**. Venn diagram showing the total number of genes expressed in macrophages and osteoclasts at 1 dphs, with the overlapping region showing commonly expressed genes. **B**. KEGG pathway enrichment analysis of differentially expressed genes (DEGs) showing significantly enriched categories including phagosome, focal adhesion, cytokine-cytokine receptor interaction, and extracellular matrix-receptor interaction. **C**. Volcano plot showing up- and downregulated genes in osteoclasts compared to macrophages with adjusted P-values less than 0.05 (p-adj< 0.05). Insignificantly regulated genes are shown as blue dots. **D**. List of genes with the highest regulation indicated by log_2_ fold change; all genes have adjusted P-values < 0.05.

**Figure 5. Macrophage recruitment and differentiation are impaired in *tnfa* mutants.**

**A.** Wild-type siblings, heterozygous and homozygous *tnfa* mutants with *rankl:HSE:cfp*/*mpeg1:mCherry-F*/*ctsk:GFP* transgenic background were heat shocked at 9 dpf for 2 hours. Embryos without *rankl:HSE:cfp* transgene were used as controls (-RANKL). Blue box on bright field image depicts area imaged. White boxes encircle individual vertebrae. In -RANKL embryos, the distribution of macrophages in *tnfa* mutants is not different from that in wild-type siblings (*tnfa^+/+^*). Upon Rankl induction (+RANKL), there are less macrophages recruited in *tnfa^-/-^* embryos compared to the heterozygous or wild-type siblings. Also, numbers of total macrophages and ectopic osteoclasts in homozygous mutants are significantly less than in wild-type embryos. **B-D.** Quantification of recruited macrophages, total macrophages, and ectopic osteoclasts, respectively. Error bars indicate mean numbers ± s.d., ** p<0.01, ns, not significant, unpaired, two-tailed Student’s t-test. **E.** Calcein staining shows normal mineralization in *tnfa*^+/-^ and *tnfa*^-/-^ mutants and wildtype siblings in the absence of Rankl induction. After Rankl induction, *tnfa*^-/-^ mutants exhibit less bone resorption with minor lesions (cyan arrowheads), compared to the severe defects in heterozygous and wild-type embryos (yellow arrowheads). Scale bars: 100 μm (in A), 1 mm (in E, low magnification), 200 μm (in E, high magnification).

**Figure 6. Chemical inhibition of *tnfa* impedes osteoclast formation.**

**A.** Heat-induced Rankl negative *mpeg1:mCherry-F/ctsk:GFP* transgenic embryos treated with DMSO or PTX showed normal macrophage density and distribution. **B.** *rankl:HSE:cfp/mpeg1:mCherry-F/ctsk:GFP* transgenic embryos were heat-shocked for Rankl induction and immediately treated with PTX or DMSO. Upon Rankl induction, macrophage recruitment was not significantly different in DMSO- and PTX-treated larvae (white arrowheads), but osteoclast formation was strongly impaired in PTX-treated embryos at 1 and 2 dphs (green arrowheads). **C-D**. Quantification showed that the total number of macrophages at 1 dphs was slightly higher in DMSO control than in PTX treated embryos, but macrophage recruitment was not significantly different in both samples. **E**. Quantification of ectopic osteoclasts shows significantly less osteoclast formation in PTX treated embryos at 1 and 2 dphs. Quantifications in **C, D, E** show mean number of macrophages and osteoclasts ± SD, Student’s t-test (two-tailed, unpaired), 7≤N­_DMSO_Rankl-_≤9, 22≤N­_DMSO_Rankl+_≤24, 12≤N­_PTX_Rankl-_≤16, 27≤N­_PTX_Rankl+_≤28, *p˂0.05, ***p˂0.001, ns: non significant, data from three independent experiments. Mφ: macrophage; OC: osteoclast. Scale bar: 200 μm (in **B**), 1 mm (in **A**).

**SUPPLEMENTARY TABLE 1**

| **Primers for amplification of *mpeg1* promoter** | |
| --- | --- |
| *mpeg1.2F* | ATAGCGAGCGGATCCAGAACAGTTTCTGGCAGAAGTTAGTC |
| *mpeg1.2R* | AATATGCGGCCGCTGAATTCATCCATGTTCCGCAGGTTGTC |
| **Guide RNA sequences targeting *tnfa*** | |
| gRNA1 | CCCTCTGTTTAGGAGGCGCCCGG |
| gRNA2 | TTGTAAATGGATGGCCGCCTTGG |
| gRNA3 | CAAACCGGTGCACAGGTCGCCGG |
| **Primers for genotyping *tnfa* mutants** | |
| Forward 1 | GGCGCAGCATCCAGACGACAAA |
| Forward 2 | ACAGGATCCAGCGCTTCTCAA |
| Reverse | CACACCAAAGAACGTGCTGCC |
| **qPCR primers** | |
| *tnfa* Forward | GTCCTACAGAGCTGCCAGAAAC |
| *tnfa* Reverse | CCCACTTCAGCACAGAGTTTTCC |
| *col6a1* Forward | AATCTCACAGCCAAGATCTGCC |
| *col6a1* Reverse | TGGCTGAGCTGTCCATCATG |
| *col6a2* Forward | ATGTCCCGATCTTCCATGCAAATC |
| *col6a2* Reverse | ATCCTCTCAGAACCATCCAGCAG |
| *col6a3* Forward | AGACGGAGCAGGTGGAGATTC |
| *col6a3* Reverse | ACCATTTCAGGGCGAACTCTG |
| *col12a1b* Forward | TCAGCAGGAAGAAACGGGTTC |
| *col12a1b* Reverse | TGAAAGTCCTCGTTCACCCTTCTC |
| *itgb3* Forward | TGGTTACCATCCACGACAGG |
| *itgb3* Reverse | TTGATGTTGCTCCTTTGTAAAGCGG |
| *itga2.2* Forward | TGGAAACCTCTGACCCAAATCTGC |
| *itga2.2* Reverse | AACGTCAGCTGTTTCTCCAGG |
| *itgav* Forward | AGTCCAAAGATGCAGAGAACAACTTG |
| *itgav* Reverse | TCTTCTCAAGACGACCCACCTG |
| *mmp9* Forward | CAGACCTGGCAGAGAGCTATCTAA |
| *mmp9* Reverse | GTCCAGTTGTCCTGTCTCGTCT |
| *nfatc1* Forward | TACAGGGCTGATGGATATGCCAC |
| *nfatc1* Reverse | TGTAAGGCCCTGAGGTACACG |
| *ctsk* Forward | AGACATCAATCACGCCGTGC |
| *ctsk* Reverse | TCAGGACGTAGCCCTTGTTG |
| *trap* Forward | AGAGGAGGAGCTGAGTTGAGC |
| *trap* Reverse | TGGAGAGAAAAGGGACTGCCATC |
| *ccr1* Forward | TGGAACGCTTCGCATGTAAACAAAG |
| *ccr1* Reverse | AATCCGTTGTGGTGCTAGCG |
| *socs1b* Forward | AACTCTCCCATGCTCCCATCG |
| *socs1b* Reverse | TTGTGGGGCCATCTTCACTTTG |
| *sh2dp1* Forward | ATCCGAGACAGCGAGACCATC |
| *sh2dp1* Reverse | TGTAGTTTCCTGAGTGGGTCTGG |
| *fes* Forward | TGACGCCCTACACAAGCATG |
| *fes* Reverse | AGCTGCTCATCATCCTGGAAATTTC |
| *il22ra2b* Forward | TGTGCAACTATGTGAAGGGTGG |
| *il22ra2b* Reverse | AGATTCTTATCCGGGTAGTGCTGG |
| *il22ra2a* Forward | AAGCCATGAGAAGTGCATCACC |
| *il22ra2a* Reverse | TCGTCGAGGTAGCTCAGATGG |
| *sbno2* Forward | ACCTTCCTGACACCTGGAAATCC |
| *sbno2* Reverse | TACTGTACGCCTCTTCCCAGG |
| *pip5k1ba* Forward | ACTGACACAGACTCACCCACAG |
| *pip5k1ba* Reverse | TGGGGGTTCACATGTTCAGAGG |
| *b-actin* Forward | GCCAACAGGGAGAAGATGAC |
| *b-actin* Reverse | CATCACCAGAGTCCATGACG |

**SUPPLEMENTARY FIGURES**

**Figure S1.** **Macrophages become less motile after differentiation into osteoclasts.**

**A**. Cell tracking showed that most macrophages remained in the AGM of -Rankl embryos. On the contrary, many macrophages were directionally recruited towards bone matrix of the vertebral column upon Rankl induction. The recruited cells clustered mainly at neural arches and vertebral bodies but not in the intervertebral discs. Macrophage dynamics was determined at 4 hphs. **B**. At 1-2 dphs, macrophages that differentiated into osteoclasts (*mpeg+/ctsk+*) have significantly reduced motility and are confined to bone matrix. Macrophages that were recruited later stayed undifferentiated and remained highly motile. As the cells were more dynamic, they had angular shapes with lower sphericity. In contrast, mature osteoclasts became more static with rounded shapes (high sphericity) after fusion. Macrophages dynamics was determined at 34 hphs. Student’s t-test was used to determine the significance of difference between groups, error bars show standard deviation. AGM: aorta gonad mesonephros; V: vertebral column; iv: intervertebral disc; vb: vertebral body; na: neural arch; Mφ: macrophage; OC: osteoclast. Scale bar: 100 μm.

**Figure S2.** **Macrophages clear apoptotic osteoclasts.**

**A**. Still images from a time-lapse movie recorded at 30 hphs. Circles show that apoptotic osteoclasts (green cells) at the neural arch are engulfed and digested by conventional phagocytosing macrophages. Time:hh:mm. **B**. Immunostaining of cleaved-Caspase-3 on cryosections revealed apoptosis (white arrowheads) of osteoclasts in Rankl-induced embryo. Scale bar: 20 μm (in **A**), 40 μm (in **B**).

**Figure S3.** **Quantification of macrophage and osteoclast numbers.**

**A**. *rankl:HSE:cfp/mpeg1:mCherry-F/ctsk:GFP* and *mpeg1:mCherry-F/ctsk:GFP* transgenic embryos were heat-shocked and imaged repeatedly at 1, 2 and 3 dphs. In the absence of ectopic Rankl expression, macrophages were distributed at different positions along the body, predominantly in the AGM (white arrowheads). There were no ectopic osteoclast in the trunk (magenta arrowheads). Upon Rankl induction, macrophages were directionally recruited towards the vertebral column, particularly to the neural arches and the vertebral bodies (cyan arrowheads). Osteoclasts were later formed by differentiation of recruited macrophages along the trunk (yellow arrowheads). The ectopic osteoclasts started to vanish at 3 dphs while new macrophages continued to be recruited. **B**. Quantification showed mean numbers of recruited macrophages, total macrophages and ectopic osteoclasts ± SD; Student’s t-test (two-tailed, unpaired), **p˂0.01, ****p˂0.0001, ns: non-significant, 10≤N­_Larvae_≤19, data from three independent experiments. Mφ: macrophage; OC: osteoclast. Scale bar: 1 mm.

**Figure S4. Gene Ontology analysis of up- and downregulated genes in osteoclast compared to macrophage.**

**A, B.** Functional groups in down- and upregulated genes, respectively. Listed genes were used as input for GO terms analysis using ShinyGO. Upregulated genes involved in osteoblast differentiation and bone remodeling are labelled in blue, and genes related to adhesion were underlined (B).

**Figure S5. qPCR validation of gene expression in FAC-sorted macrophages and osteoclasts.**

Gene expression was determined at 1 dphs. **A**. Among downregulated genes, *il22ra2a, il22ra2b* and *pip5k1ba* showed trends of downregulation in osteoclasts compared to macrophages, consistent with RNAseq data. In contrast, *cx3cr1* and *fes* show upregulation, while *socs1b, sh2dp1* and *sbno2* were not regulated. **B**. Among upregulated genes, genes encoding collagen VI, collagen XII and integrin subunit beta 3 and their binding subunits were significantly higher expressed in osteoclasts, consistent with RNAseq data. Similarly, expression of osteoclast makers *nfatc1, trap* and *ctsk* were also upregulated. Error bars indicate mean fold change ± SD, *p<0.05, **p<0.01, ***p<0.001, ns: non-significant, Student’s t-test, data were obtained from three biological replicates with three technical repeats each and β-actin was used as loading control.

**Figure S6. Phylogeny analysis of CC chemokine receptors.**

A molecular phylogeny was computed for all so far identified medaka Ccr receptors as well as human CCRs 1 – 9, human CX3R and known orthologs from the clawed frog *Xenopus tropicalis*, spotted gar *Lepisosteus oculatus* and zebrafish *Danio rerio*. In the resulting tree, the medaka receptor ENSORLP00000035998 (red arrow) identified in this study is nested together with the human CX3C chemokine receptor (blue arrow). This orthology assignment is confirmed by conserved synteny with *ccr8* and *entpd3* in medaka and human, and also in spotted gar and zebrafish. We therefore refer to the encoding gene as medaka *cx3cr-like*. Interestingly, this gene underwent independent lineage specific local gene duplications in zebrafish and medaka, while the other chemokine receptors have no such paralogs.

**Figure S7. Phylogeny analysis of interleukin receptors.**

A molecular phylogeny was computed for proteins annotated as Il22ra2 and Il20ra in ENSEMBL (ENSORLP00000023762, ENSORLP00000033651; red arrows), respectively. This showed that both proteins cluster together with human IL22RA2 (blue arrow) and thus likely represent the product of a fish-specific gene duplication. We therefore refer to the encoding genes as *il22ra2a* and *il22ra2b*. Conserved synteny with *lfngr1* was shown for *il22ra2a* that is present in human, zebrafish, spotted gar and medaka genomes. No synteny relation was detected for *il22ra2b* outside Perciformes, thus this gene represents a specific local gene duplication in this lineage.

**Figure S8. *tnfa* expression and mutant establishment in medaka**.

**A.** *tnfa* expression was analyzed by qPCR at 1, 2 and 3 dphs. Non-heat shocked embryos were used as control. In embryos without Rankl transgene (-Rankl), *tnfa* was downregulated after heat shock. In contrast, *tnfa* was upregulated in Rankl-induced embryos (+Rankl) at 1 dphs, and significantly reduced at 2 and 3 dphs. Error bars indicate mean fold change ± SD, *0.01<p<0.05, **p<0.01, ***p<0.001, Student’s t-test, data from three sets of biological samples. **B.** Design of guide RNAs for CRISPR/Cas9 to generate *tnfa* mutants. Three primers were used for genotyping. FP2 was used to differentiate between homozygous and heterozygous mutants. **C.** Representative gel image showing PCR-based genotyping. **D.** 1010 bp deletion in *tnfa* leads to a predicted truncated protein.

**Figure S9. Bone protection in PTX-treated embryos during Rankl induction.**

Embryos were stained with Alizarin Red at 3 dphs. Both DMSO control and PTX-treated larvae showed normal bone development in the absence of ectopic Rankl (blue arrowheads). Upon Rankl induction, control embryos exhibited severe bone loss in arches and vertebral bodies (black arrowheads). PTX-treated embryos, on the other hand, showed only few mild lesions in arches (small black arrowheads) in otherwise normally mineralized centra (blue arrowheads). Scale bar: 500 μm.

**SUPPLEMENTARY MOVIES**

**Movie S1. Macrophage behavior without Rankl overexpression.**

The 9 dpf *mpeg1:mCherry-F/ctsk:GFP* transgenic embryo was incubated at 39^0^C for 2 hours and subjected to confocal imaging at 2 hphs. Tracks showed mpeg1*-*positive macrophages actively patrolled around the body. Most macrophages were found in the AGM while only a few cells located proximal to the arches and vertebral bodies. AGM: aorta gonad mesonephros; VC: vertebral column; time: hh:mm:ss. Scale bar: 50 μm.

**Movie S2. Macrophage recruitment upon Rankl induction.**

Transgenic embryo *rankl:HSE:cfp/mpeg1:mCherry-F/ctsk:GFP* was induced for Rankl overexpression by heat-shock at 9 dpf and subjected to confocal imaging at 2 hphs. Mpeg1*-*positive macrophages were massively recruited toward the vertebral column. The cells dynamically localized themselves particularly at the ossified matrix, neural arches, and vertebral bodies, but not at the intervertebral disc region. Boxes outline the vertebral bodies. AGM: aorta gonad mesonephros; V: vertebral body; na: neural arch; time: hh:mm:ss.

**Movie S3. Macrophage differentiation and clearance of apoptotic osteoclasts.**

Rankl-induced *rankl:HSE:cfp/mpeg1:mCherry-F/ctsk:GFP* embryo was subjected for confocal time-lapse at 30 hphs. Arrowheads showed recruited macrophages along the trunk gradually differentiated into ctsk-positive cells. Box highlighted the phagocytosis of apoptotic osteoclasts by macrophages. AGM: aorta gonad mesonephros; na: neural arch; time: hh:mm. Scale bar: 50 μm.

**Movie S4. Fusion of differentiating osteoclasts.**

A Rankl-induced *rankl:HSE:cfp/mpeg1:mCherry-F/ctsk:GFP* embryo was subjected to confocal time-lapse at 54 hphs. During terminal differentiation, osteoclasts fused together into larger cells, both at the arches and the centra (blue boxes). White box showed an apoptosis event. AGM: aorta gonad mesonephros; time: hh:mm:ss. Scale bar: 50 μm.

**Movie S5. Normal osteoclast formation in Lipo-PBS injected embryo.**

A transgenic *rankl:HSE:cfp/mpeg1:mCherry-F/ctsk:GFP* embryo was injected with Lipo-PBS, induced for Rankl and then subjected to confocal imaging at 1 dphs. Osteoclasts formed normally from the recruited macrophages (white arrowheads). AGM: aorta gonad mesonephros; VC: vertebral column; time: hh:mm. Scale bar: 50 μm.

**Movie S6. Absence of osteoclast formation in macrophage-depleted embryo.**

A macrophage-ablated transgenic *rankl:HSE:cfp/mpeg1:mCherry-F/ctsk:GFP* embryo was induced for Rankl and subjected to confocal imaging at 1 dphs. Osteoclast formation was impaired after macrophages were ablated. AGM: aorta gonad mesonephros; VC: vertebral column; time: hh:mm. Scale bar: 50 μm.

**Movie S7.** **Multiplication of recruited macrophages and dynamic macrophage interaction with bone cells.**

A transgenic *rankl:HSE:cfp/col10a1:GFP/mpeg1:mCherry-F* embryo at 9 dpf was Rankl-induced at 39^o^C for 2 hours. Time-lapse movie was started at 6 hphs. Arrows show macrophage multiplication upon recruitment to the vertebral column. White box highlights the dynamic interaction of macrophages with osteoblast precursors (*col10a1* cells). Time: hh:mm:ss. Scale bar: 50 μm.
